# Evaluation of the Reproductive and Developmental Risks of Caffeine

**DOI:** 10.1002/bdrb.20288

**Published:** 2011-04

**Authors:** Robert L Brent, Mildred S Christian, Robert M Diener

**Affiliations:** 1 Thomas Jefferson University, Alfred I. duPont Hospital for ChildrenWilmington, Delaware; 2 Argus International, Inc.Horsham, Pennsylvania; 3 185 Aster CourtWhite House Station, New Jersey

**Keywords:** caffeine, spontaneous abortion, congenital malformations, growth retardation, toxicokinetics, biological plausibility

## Abstract

A risk analysis of in utero caffeine exposure is presented utilizing epidemiological studies and animal studies dealing with congenital malformation, pregnancy loss, and weight reduction. These effects are of interest to teratologists, because animal studies are useful in their evaluation. Many of the epidemiology studies did not evaluate the impact of the “pregnancy signal,” which identifies healthy pregnancies and permits investigators to identify subjects with low pregnancy risks. The spontaneous abortion epidemiology studies were inconsistent and the majority did not consider the confounding introduced by not considering the pregnancy signal. The animal studies do not support the concept that caffeine is an abortafacient for the wide range of human caffeine exposures. Almost all the congenital malformation epidemiology studies were negative. Animal pharmacokinetic studies indicate that the teratogenic plasma level of caffeine has to reach or exceed 60 µg/ml, which is not attainable from ingesting large amounts of caffeine in foods and beverages. No epidemiological study described the “caffeine teratogenic syndrome.” Six of the 17 recent epidemiology studies dealing with the risk of caffeine and fetal weight reduction were negative. Seven of the positive studies had growth reductions that were clinically insignificant and none of the studies cited the animal literature. Analysis of caffeine's reproductive toxicity considers reproducibility and plausibility of clinical, epidemiological, and animal data. Moderate or even high amounts of beverages and foods containing caffeine do not increase the risks of congenital malformations, miscarriage or growth retardation. Pharmacokinetic studies markedly improve the ability to perform the risk analyses. *Birth Defects Res (Part B)* 92:152–187, 2011. © 2011 Wiley-Liss, Inc.

## INTRODUCTION AND GOALSOF THIS REVIEW

We (Brent and Christian) received a request from the Caffeine Committee of the International Life Science Institute (ILSI) in 2008 to update our 2001 review since many publications dealing with the effects of caffeine had been published (Christian and Brent, 2001). A current literature review of human epidemiology studies, animal studies, and caffeine toxicology studies was performed using the Medline and Toxline data bases, articles in the author's files, and publications containing important relevant information published earlier than 2001.

### Goals of This Review

One of the reasons that epidemiologists have focused so much attention on the effects of caffeine is that caffeine is the most widely used CNS stimulant in the world. At doses achieved in normal human consumption, the main effect mediated by caffeine is interaction with the adenosine receptor, as well as with adrenergic, cholinergic, GABA, and serotonin receptors (Shi et al., 1993; Leon, 2005a, b).

We recognize that well-planned epidemiology studies are the most useful for performing accurate human risk assessment. When epidemiological studies are inconsistent, animal studies that utilize exposures that occur in humans can provide additional information that is necessary to perform a risk analysis. Animal studies are most useful if plasma and tissue blood levels of caffeine and/or caffeine metabolites are measured and can be compared with human exposures. We planned to use the same protocol for estimating the human risks of developmental and reproductive problems that were utilized in the 2001 caffeine review (Christian and Brent, 2001) (Table 1). The data reviewed in this manuscript are divided into three sections: Epidemiology studies, Animal and in vitro toxicology studies, and Pharmacokinetic studies.

**Table 1:** Evaluating the Allegation of Teratogenicity

*Epidemiological Studies*: Controlled epidemiological studies consistently demonstrate an increased incidence of a particular spectrum of embryonic and/or fetal effects in exposed human populations
*Secular Trend Data*: Secular trends demonstrate a positive relationship between the changing exposures to a common environmental agent in human populations and the incidence of a particular embryonic and/or fetal effect
*Animal Developmental Toxicity Studies*: An animal model can be developed, which mimics the human developmental effect at clinically comparable exposures. Since mimicry may not occur in all animal species, animal models are more likely to be developed once there is good evidence for the embryotoxic effects reported in the human. Developmental toxicity studies in animals are indicative of a potential hazard in general rather than the potential for a specific adverse effect on the fetus when there are no human data on which to base the animal experiments
*Dose–Response Relationship*: Developmental toxicity in the human increases with dose (exposure) and the developmental toxicity in animal occurs at a dose that is pharmacokinetically (quantitatively) equivalent to the human exposure
*Biological Plausibility*: The mechanisms of developmental toxicity are understood and the effects are biologically plausible
(a) Mechanisms
(b) Receptor agonistic or antagonistic studies
(c) Enzyme suppression
(d) Nature of the malformations
(e) Teratology principles

Modified from Brent (1986b).

## EPIDEMIOLOGY STUDIES

We have reviewed human epidemiology publications that deal with

1. Pregnancy loss (miscarriage and spontaneous abortion [SAs])
2. Congenital malformations CMs, and
3. Fetal growth retardation (IUGR, SGA).

Although some of the epidemiological studies have examined more than one developmental effect, many of the studies have focused on one developmental endpoint. Two of the important studies cited and discussed in our 2001 caffeine review (Christian and Brent, 2001) were performed by Klebanoff et al. (1998, 1999). The reason for their importance is that the exposure to caffeine was determined pharmacokinetically by measuring serum caffeine and paraxanthine concentrations.

### Etiology of SA (Miscarriage and Pregnancy Loss)

Concern about the risk of SA from exposure to caffeine was one of the reasons for preparing this review. Many of the epidemiological studies fail to assess the factors that can alter the accuracy of epidemiological studies dealing with SA.

#### Causes of SA

SAs, frequently referred to as miscarriages by the public, are common occurrences during pregnancy. According to the World Health Organization, 15% (with a large standard deviation) of women who know that they are clinically pregnant spontaneously abort. Research studies indicate that a higher percentage of embryos are spontaneously aborted before the first-missed menstrual period before the mothers know that they are pregnant (Tables 3 and 4). The lay population and the news media are under the impression that many SAs are due to exposures to some type of toxic agent during the woman's pregnancy. This is an erroneous conclusion since most early SAs are due to chromosome abnormalities that are determined before conception because of chromosome aberrations that are inherited, occur during the development of the sperm, or the mother's ova (eggs). Some maternal diseases can also be responsible (Tables 3–5). Fifty to 60% of the early spontaneously aborted fetuses have chromosomal abnormalities (Bernirschke, 1974; Boue et al., 1975; Simpson, 1980).It has been estimated that up to 30–40% of all fertilized ova in the human are lost within the first three weeks of development (Hertig, 1967). This means that SAs are a common event and are due to many causes (Table 3). SAs can result from inherited or acquired chromosomal abnormalities, inherited diseases, medically or environmentally produced blighted (malformed) embryos, maternal illness, lupus anticoagulant factor (WHO, 1970; Stein et al., 1975; Kline and Stein, 1985; Beckman and Brent, 1986; Abenhaim and Lert, 1991). A more complete list of the causes of SAs is in Table 3.

**Table 3:** Etiology of Abortion

1.	Chromosomal abnormalities: pre-conceptional or periconceptional etiology
2.	Embryos and fetuses with severe congenital malformations or growth retardation
3.	Endometriosis
4.	Lupus anticoagulant (antiphospholipid antibodies) and other immunological problems related to reproduction
5.	Cervicitis; bacterial or viral infection (Kriel et al., 1970; Mead, 1989)
6.	Uterine abnormalities: subserosal myoma or hematoma, infantile uterus, bifid uterus, IUD, etc. (8–10% of recurrent aborters)
7.	Some teratogens, especially those with cytotoxic properties and endocrine disrupters (RU 486)
8.	Maternal diabetes, alcoholism, hypothyroidism, illicit drug abuse, maternal phenylketonuria, hemorrhagic diatheses, and many other chronic and acute maternal diseases
9.	Luteal phase hormonal deficiency
10.	Trauma, IUDs, lightening and other rare miscellaneous events
11.	Hypersecretion of LH
12.	Hyperandrogenemia
13.	Hyperprolactinemia
14.	Autoimmune thyroid disease
15.	Thrombophilic abnormalities other than antiphospholipid antibody
16.	Vitamin B 12 deficiency
17.	Elevated glutathione levels
18.	Dietary factors; decreased with fruits and vegetable, increased with diet rich in fats
19.	Twenty-seven percent of women with habitual abortion had a mutation G1691A in Factor V gene (Leiden mutation) of mutation C677T in the methylenetetrahydrofolate reductase gene. The Leiden mutation may play a considerable role for women having primary recurrent abortions
20.	Fourteen percent of women with unexplained recurrent abortion show highly skewed X-chromosome inactivation, which shows that they are carriers of X-linked lethal traits
21.	IgG auto anti-laminin antibodies and recurrent abortion
22.	HLA-G genotype and recurrent abortion
23.	TH 1 type response associated with recurrent abortion (cytokines)

**Table 4:** Etiology of Human Congenital Malformations Observed During the First Year of Life^a^

Suspected cause	Percent of total
*Unknown*	65 to 75
Polygenic	
Multi factorial (gene-environment interactions)	
Spontaneous errors of development	
Synergistic interactions of teratogens	
*Genetic*	10 to 25
Autosomal and sex-linked genetic disease	
New mutations	
Cytogenetic (chromosomal abnormalities)	
*Environmenta*	10
Maternal conditions: Alcoholism; diabetes; endocrinopathies; phenylketonuria; smoking and nicotine; starvation; nutritional, hyperthermia	4
Infectious agents: Rubella, toxoplasmosis, syphilis, herpes, cytomegalic inclusion disease, varicella, Venezuelan equine encephalitis, parvo virus B19	3
Mechanical problems (deformations): Amniotic band constrictions; umbilical cord constraint; disparity in uterine size and uterine contents	1 to 2
Chemicals, prescription drugs, high dose ionizing radiation	2 to 3

a Adapted from Brent (1976, 1985, 1999, 2004, 2008) and Brent and Holmes (1988).

**Table 5:** Principles of Teratology

1.	Exposure to teratogens follows a toxicological dose–response curve. There is a threshold below which no teratogenic effect will be observed, and as the dose of the teratogen is increased, both the severity and frequency of reproductive effects will increase
2.	The embryonic stage of exposure is critical in determining what deleterious effects will be produced and whether any of these effects can be produced by a known teratogen. Some teratogenic effects have a broad, and others, a very narrow period of vulnerability. The most sensitive stage for the induction of mental retardation from ionizing radiation is from the 8th to 15th week of pregnancy, a lengthy period. Thalidomide's period of vulnerability is approximately two weeks
3.	Even the most potent teratogenic agent cannot produce every malformation
4.	Most teratogens have a confined group of congenital malformations that result after exposure during a critical period of embryonic development. This confined group of malformations is referred to as the syndrome that describes the agent's teratogenic effects
5.	While a group of malformations may suggest the possibility of certain teratogens, they cannot definitively confirm the causal agent because some teratogenic syndromes mimic genetic syndromes. On the other hand, the presence of certain malformations can eliminate the possibility that a particular teratogenic agent was responsible because those malformations have not been demonstrated to be part of the syndrome or because the production of that malformation is not biologically plausible for that particular alleged teratogen

Epidemiological investigations dealing with the causes of SAs must deal with formidable problems:

1. A majority of SAs that occur early in pregnancy are due to chromosomal abnormalities that are unrelated to environmental exposures during pregnancy (Tables 2^4^ and 5).
2. The risk of abortion changes with each day of pregnancy, so that it is essential to properly match controls, to eliminate the selection of two populations with different background SA rates (Table 2).
3. Attempts to control for the hidden incidence of medical abortions have only limited success (Susser, 1983; Olsen, 1984). “The existence of high rates of medically induced abortion in the population may distort currently employed measures of the rate of SAs” (Susser, 1983). Susser indicated that women not infrequently would report medically induced abortions as SAs (“The Susser effect”).
4. Reduction of coffee consumption and aversion to other odors and tastes is one of the earliest responses of the “pregnancy signal” that occurs in healthy pregnancies, during the pregnancy stages when chorionic gonadotropin (HCG) is at a high level. The “pregnancy signal” tends to separate the healthy pregnancies from the less healthy ones. Ignoring the importance of the pregnancy symptoms can seriously undermine the accuracy of SA and other reproductive toxicity studies.

**Table 2:** Estimated Pregnancy Loss in 100 Pregnancies Versus Time From Conception

Time from conception	Percent survival to term^a^	Percent loss during interval^a^
*Preimplantation*		
0–6 days	25	54.55
*Postimplantation*		
7–13 days	55	24.66
14–20 days	73	8.18
3–5 weeks	79.5	7.56
6–9 week	90	6.52
10–13 week	92	4.42
14–17 week	96.26	1.33
18–21 week	97.56	0.85
22–25 week	98.39	0.31
26–29 week	98.69	0.30
30–33 week	98.98	0.30
34–37 week	99.26	0.34
38 week^b^	99.32	0.68

The etiology of these abortions is manifold and is listed in Table 3 (Kajii, 1980).

a Data from Kline et al. (1980). An estimated 50 to 70% of all human conceptions are lost in the first 30 weeks of gestation and 78% are lost before term.

b Modified from Schardein (2000).

### SA Articles That Will Be Discussed

Cnattinglus et al. (2000), Zusterzeel et al. (2000) Signorello et al. (2001), Wen et al. (2001), Klonoff-Cohen et al. (2002), Giannelli et al. (2003), Rasch (2003), Tolstrup et al. (2003), Khoury et al. (2004), Sata et al. (2005), George et al. (2006), Karypidis et al. (2006), Maconochie et al. (2007), Savitz (2008), Weng et al. (2008), Greenwood et al. (2010).

In the Cnattinglus et al. (2000) and the Maconochie et al. (2007) studies there were no increased risks in the groups exposed to more than 500 mg/day of caffeine.

The Cnattinglus et al. (2000) publication was one of the few caffeine studies to consider the fetal karyotype. These authors also obtained information concerning nausea and vomiting symptoms; however, these data were insufficient to evaluate the pregnancy signal. In some of the studies there was no control for the presence or absence of the “pregnancy signal.” The authors attempted to control for many potential confounding factors, but the task is monumental and unending. While they measured continine levels to evaluate smoking exposure, the authors never measured the metabolic products of caffeine to determine the actual exposure to caffeine. These studies were sophisticated and time consuming; however, they provided conflicting answers to the question of whether caffeine ingestion represents a risk for SA.

Giannelli et al. (2003) studied the effect of caffeine consumption and nausea on the risk of miscarriage (SA). Cases were women in their first pregnancy who were interviewed about 3 weeks after their pregnancy loss on average, whereas controls were interviewed at the first prenatal care visit which typically occurred at a more advanced gestational age than the SAs. Thus, the burden of recalling caffeine exposure was not equivalent for cases and controls, which represents a defect in the study design. The fact that this was not a prospective study and the cases were interviewed earlier in pregnancy than the controls may account for the results. Daily consumption of >300 mg of caffeine per day resulted in and increased risk of SA (odds ratio, OR = 1.9 [1.0–3.6]). The OR was 2.2 in group consuming >500 mg per day. A much higher proportion of controls (no SA) reported nausea and vomiting during their pregnancy. There were other confounding factors that were not evaluated that prevented the study to definitively conclude that caffeine was causally related to the occurrence of SAs.

George et al. (2006) performed a case–control study of 108 women with SAs who had two or more SAs. Controls were obtained from a population of over 500 women who had two successful pregnancies and their last pregnancy was successful. The 108 women had two or more consecutive miscarriages (cases) and agreed to participate. Mean caffeine consumption ≥300 mg/day was associated with a 2.7-fold increased odds of repeated miscarriage (95% CI = 1.1–6.2) in nonsmokers, but not in smokers. After adjustment for many confounding factors, the odds of repeated miscarriage was no longer significantly increased in heavy caffeine users (≥300 mg/day OR = 1.8, 95% CI = 0.8–3.9). Lack of control for the pregnancy signal could have provided another explanation for the association between caffeine consumption of ≥300 mg/day and odds of repeated SA in nonsmokers. Studies have observed that smokers are less likely to experience nausea and vomiting during pregnancy than nonsmokers (Weigel and Weigel, 1989; Louik et al., 2006). Although the investigators had access to the nausea and vomiting data it was not utilized to determine the importance of the pregnancy signal. Selecting a small population of repeated aborters to study the risk of abortion from caffeine exposure during pregnancy complicates the planning and interpretation of these studies (Tables 3–5).

Greenwood et al. (2010) studied caffeine exposure during pregnancy, late miscarriage, and stillbirth. According to the authors, “there are no large well-conducted effectiveness studies”. The study population included 2643 pregnant women, aged 18 to 45 years of age who were admitted to the study between 8 and 12 weeks gestational age. The pregnancies were monitored for late SAs and stillbirth. Total caffeine intake was estimated from all possible sources in the first trimester and throughout pregnancy. The adjusted data revealed a strong association between caffeine intake in the first trimester and subsequent late miscarriage between 12 and 24 weeks and stillbirth after 24 weeks. The cases ingested an average of 145 mg of caffeine per day, while the controls averaged 103 mg per day. All the OR were increased for the cases, and none of the increased OR's were statistically significant. The authors support the conclusion that caffeine intake should be limited during pregnancy. Unfortunately, the investigators did not adjust the data for the pregnancy signal. The investigators provided no mechanism for caffeine exposure in the first trimester to produce a pregnancy loss many weeks later or even in the third trimester.

Karypidis et al. (2006) performed a case–control study comparing the risks of SA associated with CYP1B1 polymorphisms and a possible interaction of these polymorphisms with caffeine consumption. CYP1B1 is an enzyme that is known to take part in the metabolism of many steroid hormones as well caffeine. Caffeine consumption was assessed and categorized in mg/day as 0 to 99, 100 to 299, 300 to 499, and ≥500. Nausea was recorded by week of gestation and scored as never (0), sometimes but not daily (1), daily but not all day (2), and daily all day (3). Vomiting was recorded by week of gestation as never (0), sometimes but not daily (1), and daily (2). Mean weekly scores were calculated for each symptom. Smoking status was determined based on plasma continine levels, with smokers defined as those with levels >15 ng/ml. Overall, there was a significant interaction between homozygosity for Val and caffeine intake, such that compared to women who were homozygous for Leu and who consumed <100 mg of caffeine per day, the odds of miscarriage was significantly elevated only in women homozygous for Val and who consumed either 100 to 299 mg caffeine per day (OR = 2.36 [95% CI = 1.39–4.98]) or >500 mg/day (OR = 3.61; 95% CI = 1.36–9.61); for genotype strata Leu/Leu and Val/Leu, no significant associations were observed between increasing levels of caffeine consumption and the increased risk of miscarriage. No significant interaction was observed between caffeine ingestion and smoking. The many confounding issues that were evaluated in the analyses limited the ability to detect associations.

Khoury et al. (2004) conducted a cohort study within a prospective cohort of women with type 1 diabetes who were pregnant or planning a pregnancy. A total of 191 pregnancies were observed between 1978 and 1985. This is a small sample size for a SA study. Consumption of one or more cups of caffeinated beverages per day during the first trimester of pregnancy was reported by 54% of the women. Clinically recognized SAs ≤20 weeks were identified in 12%. Compared to no caffeine intake, the OR were 3.8 (95% CI = 0.8–16.9) for first trimester consumption of 1 to 2 cups of caffeinated beverages per day and 5.5 (95% CI = 1.2–22.0) for ≥3 cups per day. The difficulties with this study are that the investigators did not control for the pregnancy signal and their methodology for calculating caffeine consumption was imprecise.

Klonoff-Cohen et al. (2002) evaluated the risk of miscarriage in 221 couples undergoing in vitro fertilization (IVF) and gamete intra-Fallopian transfer (GIFT). There were no observed associations for miscarriage with first trimester caffeine use. The fact that there was an increased miscarriage risk for preconception exposure to caffeine makes little sense because caffeine has minimal mutagenic potential and is unlikely to result in an increase in chromosome aberrations resulting in pregnancy loss. Assisted Reproductive Technology (ART) patients are seeking these programs because they already have reproductive problems. The failure rate in these programs has a wide standard deviation. With this small population the task to determine the contribution of caffeine to the incidence of SA is very difficult.

Maconochie et al. (2007) studied risk factors for first trimester miscarriage (SA). The investigators determined that pregnant women who experienced nausea were strongly associated with a reduced odds for a miscarriage (OR = 0.3; 95% CI = 0.25–0.36) for mild or moderate nausea and (OR = 0.07; 95% CI 0.04–0.14) for severe nausea, defined as frequent vomiting. When nausea was controlled for exposures of >500 mg/day, with OR of 1.14 (95% CI = 0.79–1.66). The authors concluded that if you did not control for nausea and vomiting in the pregnant population, the studies that demonstrate a positive association of caffeine ingestion with SA may not be valid. While there are many problems in the design of this study, the investigators did demonstrate that caffeine exposure was not associated with the increased risk of SAs if the data were adjusted for the confounding effect of the Pregnancy Signal.

In the Rasch (2003) studies, smoking, alcohol consumption, and caffeine ingestion were evaluated as risk factors for SA. Unfortunately, the investigator made no attempt to control for the pregnancy signal. An important and interesting concern is the possibility of evaluating fetal exposure following fetal demise. Since fetal demise may occur weeks before a SA is recognized, caffeine consumption may return to typical intake levels as pregnancy symptoms abate, artificially inflating estimates of caffeine use during the time period that may not be relevant. This was a large study of 303 women with documented SAs and 1168 controls. Almost half the women reported heavy caffeine consumption. SAs were increased in the group exposed to >375 mg of caffeine per day (OR = 2.2 [1.5–3.2]). Without controlling for nausea and vomiting symptoms it is not possible to verify a causal relationship to the caffeine exposure.

Sata et al. (2005) studied caffeine intake, CYP1A2 polymorphism, and the risk of recurrent pregnancy loss in a case–control study that reported no overall association between caffeine intake ≥300 mg/day and recurrent pregnancy loss (OR = 1.82; 95% CI = 0.72–4.58). The concept of the investigators was that polymorphism of CYP1A2 could result in populations with the ability to rapidly metabolize caffeine and therefore be able to tolerate higher exposures of caffeine. Unfortunately, the results were not decisive. No associations were observed among women with other CYP1A2 genotypes (CC + CA) (OR = 1.03 [95% CI = 0.29–3.70] for ≥300 mg/day compared to 0–99 mg/day). There were many limitations to the study including small sample size; the pregnancy signal was not evaluated and no associations were observed between caffeine intake and recurrent pregnancy loss when analyses were conducted without regard for CYPIA2 polymorphisms. While the concept that formed the basis of this study makes biological sense, the results do not definitively support the hypotheses for an interactive effect of heavy caffeine use and CYP1A2 genotype on recurrent pregnancy losses.

Savitz (2008) evaluated caffeine consumption and the risk of SA (≤20 weeks of gestation) occurring in a cohort of 2407 pregnant women. Daily caffeine consumption was determined before pregnancy, four weeks after the last menstrual period and at the time of the interview. There was an association of an increased risk of SA obtained from the caffeine data that were provided after the SA had occurred. However, there was no increased risk of SA utilizing the caffeine consumption data that were obtained before the SA had occurred. The obvious recall bias undermined the positive results and the investigators concluded that the study showed no association between coffee consumption and total caffeine intake before or during pregnancy and risk of SA up to 20 weeks of gestation. Low level of caffeine exposure in study population restricted ability to evaluate intake >300 mg/day. The results of this study do not support an association between SA and caffeine intake before or during pregnancy at the caffeine exposures that were studied.

Tolstrup et al. (2003) performed a nested case–control study of SA within the first 28 weeks of pregnancy in a large cohort of young, nonpregnant women sampled from the Copenhagen population. The daily caffeine consumption was divided into the following groups: <75 (the reference group), 75 to 300, 301 to 500, 501 to 900, and >900 mg per day. There were 303 SAs that were ascertained. This information was obtained in a follow-up interview or from the hospital record. The validity of the study would have been improved if all the SAs had been confirmed from the hospital record and the study population had been assessed for the pregnancy signal. Only the 900 mg/day estimated exposure was statistically associated with “abortion” in their study (OR = 1.7 [1.0–3.0]). There was no increased risk in the 300 and 500 mg per day groups. This study was unusual in that pregnancy losses up to 28-week gestation were labeled as SA

Wen et al. (2001) studied the association of maternal caffeine consumption with SA. This study was a prospective cohort study of SAs in the first trimester of pregnancy. Caffeine ingestion was evaluated by periodically utilizing a food intake questionnaire. The preliminary results indicated that the risk of SA was elevated with exposures between 100 to 300 mg/day (OR = 2.0; 1.0–4.1) and greater than 300 mg/day. The risk of SA was 4.11 times higher in women who did not report nausea during the first trimester compared to those who did (29.6 vs. 7.2%). There was no statistically increased risk in the groups that ingested less than 300 mg/day and a very high RR of 5.4 in the group that ingested >300 mg/day. There was incomplete evaluation of confounding factors. Small number of SAs in all categories of caffeine consumption limited the ability to detect associations. There was lack of control for the pregnancy signal although the questionnaire did request information concerning the presence of nausea.

Weng et al. (2008) performed a prospective cohort study with data that had been utilized for several SA studies, so it is not clear whether the initial planning and collection of data had the intention to study the SA risk of caffeine exposure during pregnancy. One thousand and sixty-three (1063) women consented to be part of the study and completed the in-person interview soon after confirmation of pregnancy (median gestational age at interview was 10 weeks). Cox proportional hazards models were used to compare rates of miscarriage by caffeine exposure status, adjusted for maternal age, race, education, family income, marital status, previous miscarriage, nausea and vomiting since last menstrual period, smoking status, alcohol drinking, Jacuzzi use, and exposure to magnetic fields during pregnancy. The risk of SA was increased in the exposure group of >200 mg/day. It is interesting that these authors have already reported that magnetic fields increase the risk of miscarriage using the same population of pregnant patients (Li et al., 2002). In some instances the authors also determined that a miscarriage had occurred if the only source of the information was from the mother. In other words, they may not have medical documentation that a miscarriage had occurred. Exposures of >200 mg/day of caffeine had OR of 2.23 (95% CI = 1.34–3.69). However, when the subjects were identified as having the pregnancy signal and were in the group exposed to >200 mg/day, the risk of SA was not increased.

Signorello et al. (2001) studied the effect of caffeine consumption and nausea on the risk of miscarriage (SA). This study was conducted utilizing the same case–control study population reported in Cnattingius et al. (2000). One hundred one (101) chromosomally normal SAs that occurred between 6 and 12 weeks of gestation were compared to the 953 controls that were matched by week of gestation and area of residence from the 562 control cases. With the goal of evaluating the variability in caffeine metabolism as a risk factor for SAs, the authors estimated the activity levels of two enzymes, cytochrome P4501A2 (CYP1A2) and *N*-actyltransferase 2 (NAT2) given both are involved in the metabolism or detoxification of many drugs including caffeine. This was a well planned and work intensive study to determine whether pregnant mothers with the ability to rapidly metabolize caffeine would have a lower risk for SA at all caffeine exposure levels. Using blood samples collected at the time of the SAs for cases and at the time of the interview for controls, polymorphisms of the NAT2 gene and CYP1A2 phenotypes were determined. It is not clear why the authors did not use the blood samples to determine the metabolic products of caffeine metabolism rather than the complicated indirect CYP1A2 analysis. The investigators reported that the women with high CYP1A2 activity had an increased risk for SAs in the 100 to 299 mg/day and the ≥300 mg/day groups, but no increase in SA risks among the subjects with low CYP1A2 activity. The results were not in the anticipated direction given that the authors' hypothesis was that caffeine would be more strongly associated with SA among slow metabolizers due to slower caffeine clearance. While these studies were sophisticated and time consuming, they have provided conflicting answers to the question of whether caffeine ingestion represents a risk for SA.

Zusterzeel et al. (2000) performed a case–control study of recurrent early pregnancy loss that evaluated associations with polymorphisms in glutathione *S*-transferase (GST) and cytochrome P450 genes. The authors postulated that genetic polymorphisms in these genes may reflect impaired drug metabolism, resulting in an increased susceptibility to adverse outcomes from exposures to caffeine. The case included pregnant women who had at least two unexplained consecutive SAs occurring at <17 weeks of gestation. Coffee consumption was reported by the authors in the following categories, 1 to 5, 5 to –10, and >10 cups of coffee per day. The data show no observed associations between daily coffee intake and recurrent pregnancy loss for 1 to 5 cups and for >5 cups compared to noncoffee drinkers. Although the GSTP1b-1b polymorphism appeared to be more common among women with recurrent early pregnancy loss, the limited data presented in this paper offer no evidence to implicate a specific role for coffee intake via direct or interactive effects with GST polymorphisms.

#### Summary of caffeine exposure and the risk of SA (miscarriage)

Since 2000, 17 epidemiological studies have been published dealing with the risk of SA from exposure to caffeine. Ten were case–control studies and the number of cases ranged from 58 to 953. There were six prospective cohort studies. One study was a nested control study. Only one of the studies measured the serum levels of caffeine or its metabolites to determine the actual caffeine exposure. With regard to the exposure that was evaluated, namely number of caffeine-containing beverages for various time periods, there was no increased risk of miscarriage in the majority of studies in women who drank three cups of coffee or less per day. However, there were a few studies with increased risks for miscarriage in the lowest exposure groups.

The most serious criticism of the studies dealing with SA is that 11 of the 17 studies failed to evaluate the importance of the Pregnancy Signal (Cnattinglus et al., 2000; Wen et al., 2001; Klonoff-Cohen et al., 2002; Giannelli et al., 2003; Rasch, 2003; Tolstrup et al., 2003; Khoury et al., 2004; Sata et al., 2005; George et al., 2006; Weng et al., 2008; Greenwood et al., 2010).

Evaluating the subjects in an epidemiology study with regard to the pregnancy signal allows the investigators to identify subjects with high and low reproductive risks (Weigel and Weigel, 1989; Lawson et al., 2004; Louik et al., 2006; Boylan et al., 2008).

Positive associations of maternal coffee drinking or caffeine ingestion during pregnancy and the increased risk of SAs have been reported in epidemiological studies or reviews (Christian and Brent, 2001; Leviton and Cowan, 2002; Signorello and McLaughlin, 2004; Infante-Rivard, 2007; CARE Study Group, 2008; Weng et al., 2008). Other reviews have not found such associations, and many of the associations observed may be attributable to confounding effects of maternal cigarette smoking or nutritional factors (Christian and Brent, 2001; Leviton and Cowan, 2002; Signorello and McLaughlin, 2004; Bech et al., 2007; Maconochie et al., 2007; Savitz, 2008).

The epidemiological studies evaluating the risk of SAs from caffeine exposure have been inconsistent. Reports of maternal consumption of caffeine at the level of <300 mg/day has been associated with an increased risk for SAs. Other studies have reported that exposures of 500 to 900 mg/day are not associated with and increased risk of SAs. Which result is correct? Unfortunately, none of the epidemiology studies cited the nonhuman mammalian studies dealing with caffeine exposure and SA. The animal studies reveal that the wide range of human exposures when utilized in animal reproductive studies do not result in increased pregnancy loss in mammalian reproductive studies. (See later sections.)

### Congenital Malformations

The principles of teratology can be useful for planning epidemiology studies as well as interpreting the results (Table 5).

It is important to be cognizant of the fact that drugs and chemicals account for only a small percent of environmentally produced congenital malformations and that almost all teratogens produce a constellation of effects that is identified with the teratogen (Tables 4 and 5). This should indicate to physicians, epidemiologists, and scientists that determining whether a drug or chemical is responsible for increasing the risk for CMs is not a simple task. Statistical associations do not necessarily indicate causal associations! (Nelson and Forfar, 1971; Fedrick, 1974; Heinonen et al., 1977; Borlee et al., 1978; Linn et al., 1982; Rosenberg et al., 1982; Kurppa et al., 1983; James and Paull, 1985; Pieters, 1985; Olsen et al., 1991; Natsume et al., 2000; Torfs and Christianson, 2000; Browne, 2006; Bille et al., 2007; Browne et al., 2007; Mongraw-Chaffin et al., 2008).

Malformations were not more frequent than expected in these caffeine epidemiology studies performed before the year 2000 (Nelson and Forfar, 1971; Heinonen et al., 1977; Linn et al., 1982; Rosenberg et al., 1982; Kurppa et al., 1983; Olsen et al., 1991). In two other case–control studies, significant associations were observed with the consumption of caffeinated beverages during pregnancy among mothers of 464 anencephalic infants and 190 children with various malformations (Fedrick, 1974; Borlee et al., 1978).

In studies performed in 2000 and thereafter there were 11 epidemiological publications (Natsume et al., 2000; Torfs and Christianson, 2000; Browne, 2006; Bille et al., 2007; Browne et al., 2007; Mongraw-Chaffin et al., 2008; Collier et al., 2009; Johansen et al., 2009; Miller et al., 2009; Schmidt et al., 2009).

Bille et al. (2007) reported the association between oral clefts and first trimester maternal lifestyle factors utilizing the Danish record population that includes 100,000 pregnancies. There were 192 mothers in this cohort that gave birth to a child with an oral cleft. The investigators reported that first trimester smoking was associated with an increased risk of clefting, OR = 1.5 (95% CI = 1.05–2.14). Evaluation of the risks of coffee, tea, and alcohol found OR >1.0; however, the data were not statistically significant. The authors reported an association of drinking five or more cups of tea per day early in pregnancy among the mothers of 58 children with cleft palate only, OR = 2.9, 95% CI (1.1–5.6) for infants with isolated only cleft palate. No significant association was found with maternal coffee or cola drinking in this study among the mothers of children with cleft palate, and no associations were found among the mothers of 134 infants with cleft lip with or without cleft palate and consumption of any caffeinated beverage. Bille et al. concluded, “There is no solid evidence to support caffeine as a risk factor in humans for oral clefts” (Rosenberg et al., 1982; Levitan and Cowan, 2002; Nawrot et al., 2003). The authors also conducted sub-analyses restricted to nonsyndromic cases, which may be etiologically distinct from oral clefts that occur as part of a syndrome. In fact, this may be the incorrect approach because most teratogens produce syndromes and genetic abnormalities are an important contributor to the occurrence of isolated cleft lip and cleft palate.

Browne (2006) performed a systematic review of epidemiological studies published before 2006 and concluded that there is no evidence that maternal caffeine consumption during pregnancy increases the risk of congenital anomalies in infants.

Browne et al. (2007) reported no consistent association with maternal caffeine consumption early in pregnancy in a case–control study of 4,196 infants and 3,957 controls with various types of cardiac malformations utilizing the data from the National Birth Defects Prevention Study Program. In fact, in the analysis of the atrial septal defect incidence associated with coffee intake, the OR = 0.46 (CI = 0.28–0.75) indicated that there was a lower risk associated with caffeine exposure. The investigators concluded that the results indicated that caffeine is unlikely to be causally related to the occurrence of congenital heart malformations.

Collier et al. (2009) reported a significant association with maternal intake of 200 mg of caffeine per day or more, among the mothers of 175 infants with cleft lip with or without cleft palate and other congenital anomalies (OR = 1.7; 95% CI = 1.0–2.9). For mothers who consumed 10 to 99 mg of caffeine per day, there was also a significant association with maternal intake of 100 mg of caffeine per day or more among the mothers of 657 infants with isolated cleft palate only (OR = 1.2; 95% CI = 1.0–1.6). The lack of correction for multiple comparisons and lack of a dose effect with these associations makes a causal relationship less likely. Selecting isolated clefting malformations as potentially being produced by in utero exposure to caffeine is problematic. This malformation, which has an important genetic contribution and is frequently an isolated malformation, is unlikely to result from exposure to a teratogenic agent, since known causes of cleft palate from teratogens are syndromic (anticonvulsants, alcohol, aminopterin, and retinoids).

Johansen et al. (2009) reported an association with maternal caffeine consumption (for all beverages) during the first three months of pregnancy in a Norwegian case–control study of 573 children with isolated cleft lip with or without cleft palate (OR = 1.47; 95% CI = 1.05–2.07). There were 763 randomly selected controls. For mothers who consumed >0 but <3 cups of coffee per day; OR = 1.39 (95% CI = 1.01–1.92). For mothers who consumed three or more cups of caffeine containing beverages per day the OR = 1.59 (95% CI = 1.05–3.59). There was no association of coffee consumption early in pregnancy among the offspring with cleft palate whose mother drank >3 cups day in this study, OR = 0.96, CI (0.55–1.67). There was a negative (i.e., a protective) association with maternal tea drinking among mothers of the children with isolated cleft lip with or without cleft palate (OD = 0.72; 95% CI = 0.30–0.94) for mothers who consumed three or more cups of tea per day, and no association with maternal cola consumption or with estimated daily caffeine consumption from all sources in either group. The author's conclusion was, “There was little evidence of an association between caffeine and clefts when all sources of caffeine were considered.”

Miller et al. (2009) studied “Maternal exposure to tobacco smoke, alcohol, and caffeine, and the risk of anorectal atresia.” The data utilized in this study are from the National Birth Defects Prevention Study (NBDPS). There were 464 infants with the diagnosis of anorectal atresia and 4,940 controls. There were three exposure categories: 10 to 99, 100 to 299, and >300 mg/kg/day. The OR for all three exposure groups were 1.4, 1.3, and 1.5 respectively and all three ORs were significant. There was no increasing risk with increasing exposure. The observed association of isolated anorectal atresia with caffeine is unlikely to be causally related to caffeine exposure (Table 5).

Mongraw-Chaffin et al. (2008) conducted a nested case–control study of cryptorchidism among children born to mothers enrolled in the Collaborative Perinatal Project between 1959 and 1967. The diagnosis had to persist beyond two years of age in order to be included in the study. The investigators found an association with maternal consumption of the equivalent of three or more cups of coffee per day (OD = 1.43, 95% CI = 1.06–1.93). Selecting isolated cryptorchidism as a malformation that may be produced by in utero exposure to caffeine is problematic. This malformation, which has an important genetic contribution and is frequently an isolated malformation, is unlikely to result from exposure to caffeine.

Natsume et al. (2000) performed a case–control study of cleft lip and palate that included 306 cases of cleft lip, cleft palate, or both matched to 306 controls. The protocol of this report was lacking in detail. The investigators described the caffeine exposure in cups per week, which is inadequate. Although the analyses did not indicate that there was an increased risk of cleft lip and palate this study will not be included in the final analysis.

Slickers et al. (2008) studied maternal caffeine consumption and the risk of bilateral renal agenesis and renal hypoplasia. The data utilized in this study are from the National Birth Defects Prevention Study (NBDPS). Renal agenesis and hypoplasia has many etiologies, including genetic causes. The results were inconclusive, in that there was not a statistical increased risk with caffeine exposure. However, there were only 75 renal malformations in this case–control study, which makes any definitive interpretation problematic.

Schmidt et al. (2009) studied maternal caffeine consumption and the risk of neural tube defects (NTDs). The data utilized in this study are from the National Birth Defects Prevention Study (NBDPS). Total average daily caffeine dietary consumption was obtained during the year before pregnancy occurred for 768 mothers with children with NTDs and 4,143 control mothers and infants without NTDs. Positive associations were observed between caffeine consumption and spina bifida (OR = 1.4; 95% CI = 1.1–1.9). Interestingly, caffeinated tea consumption had a protective association (OR = 0.7, CI = 0.6–0.9). While most of the OR were greater than one, few were statistically significant. Furthermore, the mothers with the highest intake of caffeine (200–299 mg/day, >300 mg/day) did not have a statistically significant increased OR for NTDs. The discussion section of this publication is extensive and has numerous hypotheses as to why the findings indicate that caffeine causes NTDs. No mention is made of evaluating the “pregnancy signal” and its role in separating the at-risk from the low risk population. Since the vast majority of teratogenic drugs produce a teratogenic syndrome and not isolated malformations such as NTDs, their findings are not supported by one of the basic teratology principles (Table 5). The authors did not review the animal literature, which indicates that caffeine does not cause isolated NTDs. The first sentence in this publication states, “Animal studies demonstrate teratogenic effects of caffeine and human studies are inconclusive.” The first report of the teratogenicity of caffeine was published in 1960 and the dose administered was 250 mg/kg (Nishimura and Nakai, 1960). Animal studies result in teratogenesis (Christian and Brent, 2001), if the exposures are far above any possible human exposure from caffeine consumption and that epidemiological literature demonstrates that caffeine is unlikely to be a human teratogen from human dietary exposures. The authors do not report the folic acid levels in their patient populations and therefore cannot discuss the important nutritional data with regard to the role of nutrition as an etiological factor in the patients with NTDs in their study.

Torfs and Christianson (2000) examined some of the environmental risks for the occurrence of Down syndrome. The study was a population-based case–control study that identified 997 Down syndrome cases from the California Birth Defects Monitoring Program and 1,007 live born nonmalformed controls from the general population. Six months after delivery, the mothers were asked about their consumption of coffee, tea, and soft drink “around the time of conception.” Since Down syndrome is a chromosome abnormality due to the presence of an extra chromosome 21 during the maturation of the sperm or egg, caffeine, exposures during embryonic development cannot produce this abnormality. Preconception exposures to caffeine would be very unlikely to affect the maternal ova because caffeine is not considered to be mutagenic. A protective association between heavy coffee intake (≥ four or more cups per day) and Down syndrome was observed among nonsmokers (OR = 0.48; 95% CI = 0.28–0.82) but not smokers (OR = 1.64; 95% CI = 0.80–3.36). This study is of interest, but does not contribute to the evaluation of whether caffeine has a teratogenic effect. One of the several hypotheses generated by the investigators was that caffeine may have caused SAs of Down syndrome embryos, thus decreasing the incidence of Down syndrome in the high caffeine exposure group.

#### Summary of the risk of congenital malformations from dietary exposure to caffeine

It is very unlikely that the usual or even high exposures of dietary caffeine increases the risk of birth defects for pregnant mothers exposed to caffeine. Not one investigator has published the constellation of developmental abnormalities that constitutes the “caffeine teratogenic syndrome” in humans (Table 5). None of the epidemiologists have carefully examined the animal teratology or animal toxicokinetic literature to determine the magnitude of exposure necessary to produce congenital malformations. Schmidt et al. (2009) cited the original publication indicating that caffeine was teratogenic in the mouse (Nishimura and Nakai, 1960). These investigators administered 250 mg/kg i.p. to pregnant mice that resulted in vascular disruptive malformations at exposures that are never reached in humans from even high exposures of dietary caffeine.

### Fetal Weight Reduction (Small for Gestational Age [SGA])

Before the year 2000, several studies were reported that indicated that caffeine exposure during pregnancy was associated with fetal growth retardation (Mau and Netter, 1974; Martin and Bracken, 1987; Fenster et al., 1991; Peackock et al., 1991; Vlajinac et al., 1997). Other investigators have indicated that smoking may be an important confounder in caffeine fetal growth studies (Beaulac-Baillargeon and Desroisiers, 1987). Studies have also reported that the results did not indicate that caffeine exposure during pregnancy reduced fetal growth (Linn et al., 1982; Cook et al., 1996; Committee on Toxicity, 2001).

During the years from 2000 to 2010, 17 articles were published evaluating the risk of maternal caffeine exposure and fetal weight reduction (Grosso et al., 2006, 2006; Clausson et al., 2002; Klebanoff et al., 2002; Balat et al., 2003; Bracken et al., 2003; Ørskou et al., 2003; Vik et al., 2003; Parazzini et al., 2005; Santos et al., 2005; Tsubouchi et al., 2006; Bech et al., 2007; Diego et al., 2007; Infante-Rivard, 2007; Care Study Group, 2008; Xue et al., 2008; Bakker et al., 2010).

Bakker et al. (2010) examined the associations of maternal caffeine intake, based on coffee and tea consumption, with fetal linear growth and fetal weight measurements in each trimester of pregnancy and the risk of adverse birth outcomes. There were 7,346 pregnant women participating in a population-based prospective cohort study from early pregnancy onward in the Netherlands (2001–2005). Caffeine intake in the first, second, and third trimesters was on the basis of coffee and tea consumption and was assessed by questionnaires. Fetal linear growth measurements were repeatedly measured by ultrasound. Information about birth outcomes was obtained from hospital records. The investigators observed no consistent associations of caffeine intake with fetal head circumference or estimated fetal weight in any trimester. Higher caffeine intake was associated with smaller first-trimester crown-rump length, second-and third-trimester femur length, and birth length (*p* for trend <0.05). Offspring of mothers who consumed >6 caffeine units/day (540 mg) tended to have increased risks of small-for-gestational-age infants at birth. The authors concluded that caffeine intake of >6 units/day during pregnancy is associated with impaired fetal length. Caffeine exposure might preferentially adversely affect fetal skeletal growth and that further studies are needed. The actual data are more important than the conclusions. In the small group of 133 women who had the equivalent of 6 cups of coffee per day, the femur was smaller by 0.5 mm in the third trimester and the crown rump length (CRL) was reduced by 4.54 mm in the first trimester. There was no effect on birth weight, head circumference, or prematurity incidence. The CRL at term is not available in the publication. No mention is made of controlling for the pregnancy signal. The average birth weight was over 3,400 g. The only positive group for shortening of length (in millimeters) was the women who ingested 6 or more (units, 540 mg) cups of coffee per day. It was not determined whether the reduced length was recoverable or of any clinical significance. This study is more important because of the negative findings, rather than the minimal positive findings. Caffeine exposure at every exposure had no effect on birth weight. There was no increase in SGA babies in this study.

Balat et al. (2003) recruited a group of smokers (*n* = 60) and nonsmokers (*n* = 63) who delivered at full-term (37–41 weeks) to evaluate the effect of caffeine intake on newborn and placental characteristics. The investigators obtained the caffeine intake based on the average number of cups of coffee and tea consumed per day. Based on the intake, the mothers were divided into two groups: <300 mg/day or >300 mg/day, assuming 107 mg of caffeine for each cup of coffee and 34 mg for each cup of tea. This study did not attempt to control for other important confounders such as maternal age, alcohol use, and gestational age at birth. The difference in birth weight between the >300 mg/day versus the <300 mg/day group was 128 g. The results do not definitively suggest that the caffeine was responsible for this very small, clinically insignificant weight difference.

Bech et al. (2007) randomly assigned women (*n* = 1,197) to caffeinated or decaffeinated instant coffee during the last half of pregnancy. The pregnancies were followed to evaluate differences in gestational age and mean birth weight. Participants were provided unlimited amounts of coffee, either caffeinated or decaffeinated as assigned. They were also free to consume other sources of coffee and caffeinated beverages. It would appear that this study should have been altered once the investigators realized that there were no real exposed and control groups. The possible interaction between smoking and caffeine consumption on birth weight is unconvincing without presentation of the results by compliance or, preferably, by actual caffeine consumption. The investigators reported a 263 g weight reduction in the newborns from exposure to caffeine, which is clinically significant.

Bracken et al. (2003): This prospective cohort study included 2291 pregnant women ≤24 gestational weeks from clinics and obstetric practices. Caffeine exposures were evaluated as urinary caffeine and self reporting of caffeine ingestion during early and late pregnancy. The rates of IUGR (8.4%), low birth weight (4.7%), and preterm birth (7.0%) were lower in this cohort than in the general US population for the year 2000 (10, 6, and 11.6%, respectively) (León et al., 2002). There is minimal evidence in this publication to indicate that caffeine use during early or late pregnancy is related to low birth weight.

Care Study Group (2008) performed a prospective longitudinal observational study to examine the association of maternal caffeine intake with “fetal growth restriction.” During the 8th to 12th week of pregnancy 2635 women were recruited for the study. Assessments of (1) caffeine and (2) smoking and tobacco exposure were performed by self-reporting and by measuring caffeine and cotinine in the saliva. This was a large and ambitious project. There were four categories of exposure (<100, 100–199, 200–299, >300 mg of caffeine per day). The adjusted OR were calculated for the 12 groups that were evaluated. There were four groups that were not significant. The remaining OR's were significant with five of the OR's having a CI lower than 1.0. Placing this data into clinical perspective, the average difference in birth weight between the caffeine exposed and the controls in the 12 groups ranged between 21 and 89 g. Those differences in birth weight are the equivalent to less than one to three ounces, and are clinically insignificant. The authors describe these findings as associations; however, their clinical recommendations infer that the caffeine has a causal relationship and not just an association. Their recommendation is, “Sensible advice would be to reduce caffeine intake before conception and throughout pregnancy.” More appropriate advice would be to also stop smoking, limit alcohol consumption, limit vigorous exercise, limit calorie restriction, nutritional fads, and recreational drugs. This was a very large and comprehensive study; however, the investigators ignored the evaluation of the pregnancy signal for collating pregnancies into high risk and low risk categories for reproductive and developmental problems, which is a serious deficiency.

Clausson et al. (2002): A population of patients that had been part of a SA study (Cnattingius et al., 2000) was followed to evaluate the effects of caffeine use on the birth weight of the newborns. Caffeine intake was obtained for the first six weeks of gestation, second trimester, and for a portion of the third trimester. There were four exposure categories, 0 to 99, 100 to 299, 300 to 499, or ≥500 mg/day. There were no differences in birth weights in the five different, caffeine intake categories.

Diego et al. (2007): Birth weights were obtained from the medical records of 452 participants who were recruited between 20 and 28 weeks of gestation. The data pertaining to the caffeine exposure are unclear and there was no attempt to evaluate confounding factors. The exposures ranged between zero and six caffeine-containing drinks per day. The exposure assessment was problematic and therefore the weak association with weight reduction is of little value.

Grosso et al. (2001): There were over two thousand pregnant participants in this cohort study of IUGR. Cord blood samples were obtained for the analysis of caffeine metabolites from 1,606 participants. Pregnancy symptoms were not evaluated. The investigators measured cord serum caffeine, paraxanthine, theophylline, and theobromine concentrations as indicators of the amount of caffeine or its metabolites entering fetal circulation after crossing the placenta. There was no association between IUGR and caffeine intake during the first (OR = 0.91; 95% CI = 0.44–1.90) or seventh month of pregnancy (OR = 1.00; 95% CI = 0.37–2.70).

Grosso et al. (2006): The pregnancy signal was not included in their evaluation. Women in the lower caffeine exposure group delivered newborns with a reduced risk of IUGR. Women with the highest concentrations of paraxanthine had an increased risk of IUGR (OR = 3.3; 95% CI = 1.2–9.2). Fast metabolizers of caffeine and caffeine metabolic products were associated with an increased risk for IUGR (OR = 1.21; 95% CI = 1.07–1.37). The fast metabolizers should have the lowest caffeine levels and therefore, the lowest risk. This result is the opposite of what one would have predicted, which confuses the attempt to clarify the relationship of caffeine exposure and the risk of fetal weight reduction.

Infante-Rivard (2007): There were 451 cases and 451 controls born after the 24th week of gestation with no CMs in this SGA (10th percentile of less) case–control study that examined the association of caffeine exposure and the risk of fetal weight reduction. There were two exposure groups (<300 mg/day vs. ≥300 mg/day). Maternal and newborn blood samples were obtained for CYP1A2 and CYP2E1 polymorphisms genotyping. Growth retardation was not affected by the polymorphism in the mother or child. There was a very small reduction in birth weight associated with an increasing caffeine exposure in both the first and third trimester. For every 100 mg of caffeine consumed, the birth weight was reduced by 31 and 38 g for every 100 mg of caffeine consumed during the second and third trimester, respectively. These are very small reductions in newborn weights and of no clinical significance whether or not this is a causal association.

Klebanoff et al. (2002): This study measured paraxanthine, the major metabolite of caffeine, in third trimester serum samples banked for 2,515 women participating in the Collaborative Perinatal Project (CPP) between 1959 and 1966. Controls were selected from the CPP population (Klebanoff et al., 1999). SGA was defined as birth weight <10th percentile. The risk of delivering a SGA infant increased with rising serum paraxanthine concentrations, but only among smokers. Increased risk among smokers was modest (OR≈2.0 and lower) and only present for categories of paraxanthine concentrations exceeding 715 ng/ml. Apparently, there were no associations with serum caffeine concentrations. The pregnancy symptoms were not included in the evaluation, which detracts from the validity of the final analysis.

Ørskou et al. (2003): This study determined risk factors for high birth weight (>4,000 g). In a large prospective cohort study, pregnant women were selected from a cohort of over 24,000 pregnant Danish women who were interviewed at approximately 16 weeks of gestation for the average daily consumption of cups of coffee, tea, cola, and cocoa, that was converted to total caffeine intake (mg/day). The women who consumed more than 200 mg/day of caffeine were associated with a decreased risk of giving birth to a high birth weight infant (>4,000 g). This study is not directly related to the concern regarding the risk of caffeine producing newborns with SGA.

Parazzini et al. (2005) selected 555 women delivering singleton, small for gestational age (SGA <10th percentile) babies and 1,966 controls who delivered healthy, term singletons for a case–control study. Caffeine consumption was listed as the number of cups per day before pregnancy and during each trimester. The pregnancy signal data were collected on 50% of the cases and 66% of the controls. The authors observed no associations between SGA and intake of three or more cups of coffee per day during pregnancy or >4 cups of coffee per day before becoming pregnant.

Santos et al. (2005): This retrospective cohort study of 5,189 singletons was evaluated for SGA from exposure to a caffeinated beverage consumed in South America called maté. All the mothers were interviewed within the 24 hr following delivery. The investigators estimated that the daily maté consumption was equivalent to a daily average caffeine intake of 300 mg (Santos et al., 1998). The investigators controlled for eight confounding factors and concluded that mate (300 mg/day) does not increase the risk of having a SGA newborn.

Tsubouchi et al. (2006): This study is a physiological study of 10 pregnant women designed to measure whether caffeine affects maternal and fetal blood flow velocity using Doppler sonography. The pregnant women were given one cup of coffee (100 mg of caffeine) before determining maternal and fetal blood flow in the third trimester. The caffeine had no effect on blood flow in the uterine artery, fetal middle cerebral artery, or umbilical artery. This study only indicates that if fetal growth retardation is caused by exposure to caffeine, the mechanism is not via altering the blood supply to the fetus.

Vik et al. (2003): Caucasian pregnant women who were described as high risk based on their pregnancy histories were selected before the 20th week of gestation to participate in a caffeine exposure study. The high risk category included the following criteria:

1. Low birth weight,
2. Smoking,
3. Prepregnancy weight <50 kg,
4. Previous perinatal death and being chronically ill.

A complete dietary analysis was performed for each woman during various stages of pregnancy for 858 women. The mean caffeine intake at the 17th week was 232 and 205 mg/day at 33 weeks. For a 60 kg woman the caffeine exposure is 3.9 and 3.4 mg/kg, respectively. No association was observed between “high” caffeine intake at 17 weeks and giving birth to a SGA infant (OR = 1.1; 95% CI = 0.6–2.1), but high consumption at 33 weeks was associated with an increased OR for SGA (OR = 1.6; 95% CI = 1.0–2.5). Yet the findings in this study indicate that high caffeine intake did not result in an increased risk of newborns with SGA with caffeine exposures at midgestation (17 weeks). The 33-week group exposed to high exposures of caffeine did have a statistically increased risk for infants with SGA.

Xue et al. (2008): The Mothers of subjects in the Nurses' Health Study (*n* = 34,063) were sent questionnaires to collect pregnancy and newborn data that occurred many years in the past. The mothers answered a questionnaire pertaining to events that occurred 40 to 60 years ago with regard to caffeine ingestion. This is an exceptionally long period of time to expect an accurate recall of the mother's caffeine ingestion. It is a serious deficiency in this study. There were five categories of caffeine consumption corresponding to never, <1, 1 to 2, 3 to 4, and ≥5 cups per day. The authors report that birth weight was negatively associated with coffee consumption during pregnancy decreasing by 15, 34, and 54 g for consumption of 1 to 2, 3 to, 4 and ≥5 cups of coffee per day during pregnancy. These weight reductions are clinically insignificant since it is one percent or less of the weight of a newborn baby. Pregnancy symptoms were not considered as confounders.

#### Summary of the growth retardation studies

The growth retardation studies were not consistent. In six of the studies the results were negative for an association of growth retardation due to exposures to caffeine. Seven of the studies were equivocal demonstrating a risk for growth retardation with increasing exposures to caffeine but with the inability to determine the role of confounding factors. Four of the studies did not evaluate the pregnancy signal. Two of the studies were not devoted to the caffeine exposure and the risk of fetal growth retardation. In some of the positive studies, the magnitude of the growth retardation was clinically insignificant. None of the epidemiology studies examined the growth retardation studies in animals that indicated pharmacokinetically that exposures had to be significantly above even the highest caffeine exposures to which pregnant women would be exposed to produce fetal growth retardation.

## ANIMAL REPRODUCTIVE, DEVELOPMENTAL AND IN VITRO STUDIES DEALING WITH EXPOSURES TO CAFFEINE

### Reproductive and Developmental Toxicology

Review of the animal studies has revealed some interesting as well as unexpected findings. None of the results of the oral administration of caffeine indicated that caffeine increased the risk of embryonic death. While a few manuscripts reported research conducted in consideration of US (FDA) or international (ICH) guidelines, most are conducted using inappropriate routes of exposure (only a few are relevant to normal human exposure). Most oral studies were conducted at toxic levels, that is, those in excess of the 30 mg/kg/day NOEL in rodents, and only a few of the studies are relevant to normal human exposures (in general, it was not possible to extrapolate nonclinical bolus oral exposures to human exposures). The results of the review of all the papers are outlined in Supplemental Table 1, and Tables 6–9. Not all the mg/kg/day dosages are available (in some cases these can only be estimated, because of the route used). There is also inadequate information regarding concentrations, consumption, and animal body weights. Supplemental Table 1 represents a summary of the recent animal toxicology literature pertaining to caffeine.

**Table 6:** Summary of Supplemental Publications Reviewed by Species and Route of Administration

Publication	Species	Route	Dose	Investigated effect
Fenster et al. (1998)	Humans	PO daily consumption	> or <150 mg/day	Rate of caffeine metabolism and risk of spontaneous abortion
Klebanoff et al. (2002)	Humans	PO daily consumption	–	Effect of 3d trimester maternal serum concentration of paraxanthine on birth weight
du Preez et al. (1999)	Humans	IV	4.0 to 7.7 mg/kg	The clearance rate and volume of distribution of theophylline in apneic premature neonates
Mazkereth et al. (1997)	Humans	IV	6 mg/kg	Effect of aminophylline dosage on urinary output in premature infants.
Maza et al. (2001)	Rats	IV	6 mg/kg	Effect of hepatic regeneration after partial hepatectomy on theophylline pharmacokinetics
Jorritsma et al. (2000)	Rats	IP	10 mg/kg	Induction of P4501A with caffeine in therapeutic model of hyperbilirubinemia in Gunn rats
Pelissier-Alicot et al. (2002)	Rats	SC	25 mg/kg	Effect of administration (AM vs. PM) of caffeine on daily rhythms of heart rate, body temperature, and locomotor activity
Schrader et al. (1999)	Rats	Analytical method	–	Development of reverse-phase HPLC method for analyzing caffeine + all eight metabolites simultaneously from rat urine
Buters et al. (1996)	Mice	IP	2 mg/kg	Confirmed involvement of CYP1A2 in PK and metabolism of caffeine in CYP1A2^−/−^ and CYP1A2^+/+^ mice
Derkenne et al. (2005)	Mice	IP	8 mg/kg	Replaced mouse Cyp1a2 (− / −) with human CYP1A2 gene to restore metabolism of caffeine and change it to human profile
Kolarovic et al. (1999)	Mice	IP	20 mg/kg	Use of caffeine as a biomarker for the estimation of xenobiotic biotransformation and possible hepatotoxicity
Labedzki et al. (2002)	Mice/human microsomes	In vitro comparison	−	In vitro comparison of murine and human CYP1A2-mediated metabolism of caffeine and quinolones
Janus and Antoszek (2000)	Cattle	IV	5 mg/kg	Effect of gender and age on the pharmacokinetics of caffeine in Holstein cattle
Janus et al. (2001)	Cattle (calves)	IV	5 mg/kg	Effect of 4-day starvation or water deprivation on the pharmacokinetics of caffeine in calves
Peck et al. (1997)	Horses	IV	2.5 mg/kg	Compared the pharmacokinetic disposition of caffeine and its metabolites in horses and donkeys
Todi et al. (1999)	Horses	IV	2 g or less	Detection of caffeine in serum and urine after doses of caffeine or theophylline in race horses
Wasfi et al. (2000)	Camels	IV	2.35 mg/kg	The pharmacokinetics, metabolism, and urinary detection time of caffeine was characterized in camels
Fort et al. (1998)	Frog (*Xenopus*)	In vitro	−	The developmental toxicities of caffeine and 13 metabolites were investigated in the FETAX Teratogenesis Assay

PO, oral; IV, intravenous; IP, intraperitoneal; SC, subcutaneous.

**Table 7:** Summary of Publications Reviewed by Species and Route of Administration

Publication	Species	Type	Route	Gestation	Potential effect investigated	NOEL (noted only when identified)
Asadifar et al. (2005)	Rats	Sprague–Dawley	Diet	Postnatal	Effect of Cu deficiency on heart	
Hongu and Sachan (2000)	Rats	Sprague–Dawley	Diet	28 days from 7 week	Body weight changes	Postnatal study only
Burdan et al. (2002)	Rats	Wistar	Diet	GDs 8 to 14	Caffeine + OTC propyphenazone and paracetamol effect on fetal development	
Burdan et al. (2004)	Rats	Wistar	Diet	GDs 8 to 14	Caffeine + OTC propyphenazone effect on fetal development	
Nomura et al. (2004)	Rats	Not identified	Diet	In vitro	Effects on gene expressions	
da Silva et al. (2005)	Rats	Not identified	Drinking water	Not identified	Effect of maternal caffeine intake on hyperlocomotion in pups	
Bodineau et al. (2003)	Rats	Sprague–Dawley	Drinking water	Throughout gestation	Respiratory control in newborn	≥30 mg/kg/day
Aden et al. (2000)	Rats	Wistar	Drinking water	GD 2-postnatal	Adenosine receptors	
da Silva et al. (2008)	Rats	Wistar	Drinking water	Mating, gestation, part of lactation	Postnatal development	
Iglesias et al. (2006)	Rats	Wistar	Drinking water	GDs 2-end of gestation	Heart receptors—maternal and fetal	
León et al. (2002)	Rats	Wistar	Drinking water	GDs 2-end of gestation	Brain—adenosine A1 receptor in dams and fetuses	
León et al. (2005a)	Rats	Wistar	Drinking water	GDs 2-end of gestation	Maternal and fetal brain receptors	
León et al. (2005b)	Rats	Wistar	Drinking water	GDs 2-end of gestation	Brain–adenosine A1 receptor in dams and fetuses	
Saadani-Makki et al. (2004)	Rats	Sprague–Dawley	Drinking water	Beginning not specified through parturition	Involvement of adenosinergic A1 systems on respiratory perturbations	
Gaytan et al. (2006)	Rats	Sprague–Dawley	Gavage	PND 2 to 6	Adenosine A1 receptor system	
Burdan et al. (2000)	Rats	Wistar	Gavage	GDs 8 to 14	Skeletal ossification	
Burdan et al. (2001)	Rats	Wistar	Gavage	GDs 8 to 14	Caffeine + acetaminophen and isopropylanipyrine on pregnant liver	
Burdan et al. (2003)	Rats	Wistar	Gavage	GDs 8 to 14	Determine effect of paracetamol and caffeine administered together	
Evereklioglu et al. (2003)	Rats	Wistar	Intraperitoneal injection	GDs 9 to 21	Effects on neonatal rat cornea	
Evereklioglu et al. (2004)	Rats	Wistar	Intraperitoneal injection	GDs 9 to 20	Establish model for study of cataract development in rats	
Boyer et al. (2003)	Rats	Wistar-CRL	Intraperitoneal injection	Postnatal	Behavior-caffeine enhancing effect?	
Pollard et al. (2001)	Rats	Wistar CF	In vitro	Postnatal	Comparison of outcome of in vitro and in vivo studies.	
Chorostowska-Wynimko et al. (2004)	Mice	Balb/c	Diet	During pregnancy and lactation	Fetal development and postnatal status of immune system	
Björklund et al. (2007)	Mice	A1RKO; A2aRKO; WT	Drinking water	GD 7 PND 7	Adenosine receptors	Doses exceeded 30 mg/kg/day
Albina et al. (2002)	Mice	Swiss	Gavage	GDs 0 to 18	Stress and caffeine	≥30 mg/kg/day
Colomina et al. (2001)	Mice	Swiss	Gavage	GD 9	Stress and caffeine/aspirin	
Bahi et al. (2001)	Mice	Swiss	Intraperitoneal injection	PND 0; PNDs 1 to 3	Effect on lesions of periventricular white matter	Not appropriate for human extrapolation
Desfrere et al. (2007)	Mice	Not identified	Intraperitoneal injection	PNDs 3 to 10; PNDs 4 to 10	Effect on developing mouse brain	
Lutz and Beck (2000)	Mice	C57BL/6JBK	Intraperitoneal injection	GD 9	Interaction between caffeine and Cd sulfate	
Sahir et al. (2000)	Mice	Swiss	Intraperitoneal injection	GDs 8 to 10	Brain development and early encephalization	
Sahir et al. (2001)	Mice	Swiss	Intraperitoneal injection	GDs 8.5 to 10.5	Gene modulation in postimplantation mouse embryos	
Momoi et al. (2008)	Mice	CD-1	Subcutaneous injection	GDs 9.5 to 18.5	Fetal cardiovascular function affected by maternal exposure	
López and Alvariño (2000)	Rabbits	NZW—semen	In vitro		Effect of caffeine on semen	
Clyman and Roman (2007)	Sheep	–	In vitro		Effects on preterm sheep ductus arteriosus	
Tomimatsu et al. (2007)	Sheep	–	Intravenous administration		Brain-effect on fetal cerebral oxygenation	
Momozawa and Fukada (2003)	Bovine	–	In vitro		Effect of caffeine on bovine IVF	
Tatham et al. (2003)	Buffalo-Cattle	–	In vitro		Effect of caffeine on buffalo sperm to overcome male infertility	
*Miscellaneous*						
Buttar and Jones (2003)	N/A	N/A	N/A	N/A	Symposium on herbal remedies	
Gilbert-Barness (2000)	N/A	N/A	N/A	N/A	Letter to the editor	
Keller et al. (2007)	N/A	N/A	N/A	N/A	Overview—cardiovascular development	

N/A, not applicable; GD, gestation day; PND, postnatal day.

**Table 8:** Comparing the Pharmacokinetics and Toxicokinetics of Caffeine in Humans and Animals

Method of administration	Exposure	Plasma caffeine level	Teratogenic effect
1 to 2 cups of coffee/day in humans; 1 to 2 mg/kg	100 to 200 mg of caffeine	1 to 3 µg/ml peak level	Not teratogenic
3–5 cups of coffee/day in humans; 3 to 5 mg/kg	500 to 600 mg of caffeine	5 to 6 µg/ml peak level	Not teratogenic
10 cups of coffee per day over a 10-hr period	<1,000 to 1,200 mg of coffee	Speculation; <10 µg/ml peak level	Minimal data; unlikely to be teratogenic
Caffeine in the drinking water in the rat	80 mg/kg/day	5.7 ± 2.3 mg/kg/day	Not teratogenic
Caffeine in the drinking water in the rat	205 mg/kg/day	Peak ?	Not teratogenic
Caffeine by once a day gavage in the rat	80 mg/kg/day	Peak >60 µg/ml	Teratogenic
Caffeine in the drinking water in the rat	330 mg/kg/day	Peak >60 µg/ml	Teratogenic
Caffeine in drinking water in the rat	80 mg/kg/day	0.10 to 5.74 µg/ml	Not teratogenic
Caffeine bolus of 25 mg, 24 hr later in nonpregnant rat	25 mg/kg	2 µmol/l 0.4 µg/ml	A pharmacokinetic study
Caffeine bolus of 25 mg, 24 hr later in 20-day pregnant rat	25 mg/kg	20 µmol/l 4 µg/ml	A pharmacokinetic study
Human exposure during pregnancy of a mother who drank 9 to 24 cups of coffee/day (Khanna and Somani, 1984; Bodineau et al., 2003)	900 to 2,400 mg/day 9 mg/kg to 24 mg/kg/day	80 µg/ml at birth, estimated 40.3 µg/ml at the 12th postpartum day. Maternal serum level on the 10th postpartum day, 18.4 µg/ml	No teratogennesis, growth retardation. Liveborn who is doing well and was weight-appropriate for the gestational age
Food and Drug Administration recommendation (1980)	Limit caffeine to <400 mg/day (6.7 mg/kg/day for a 60-kg human)	Peak blood level will be very low	No data; Very unlikely to be teratogenic

**Table 9:** Mechanisms of Action of Environmental Teratogens

1.	Cytotoxicity or mitotic delay beyond the recuperative capacity of the embryo or fetus (ionizing radiation, chemotherapeutic agents, alcohol)
2.	Inhibition of cell migration, differentiation, and cell communication
3.	Interference with histogenesis by processes such as cell depletion, necrosis, calcification, or scarring
4.	Biologic and pharmacological receptor-mediated developmental effects (i.e., etretinate, isotretinoin, retinol, sex steroids, streptomycin, and thalidomide)
5.	Metabolic inhibition (i.e., warfarin, anticonvulsants, and nutritional deficiencies)
6.	Physical constraint, vascular disruption, inflammatory lesions, and amniotic band syndrome
7.	Interference with nutritional support of the embryo by decreasing maternal food intake or affecting yolk sac or chorioplacental function or transport

Extrapolating the results of caffeine animal toxicology studies for human risk assessment:

1. Parental (i.v, i.p., and s.c.) administration in animal study makes it difficult to perform human risk assessment. Even once a day oral intubation presents difficulties in utilizing the animal toxicology results for human risk assessment.
2. Without human serum and animal serum levels of caffeine and its metabolites, risk assessment is problematic.
3. Most animal teratology studies exposed the animals to caffeine at the appropriate stages for comparing risks in the animal model with potential risks in the human.
4. Most human exposures were measured in cups of coffee per day. However, it is difficult to define a cup (1 cup = 8 fluid ounces); coffee makers measure in 5-ounce serving cups. A 10 cup coffee maker = 50 ounces (http://www.Starbucks.com), which by standard measure = 80 ounces, a discrepancy of 30 ounces or a 27.5% difference in intake. “Cup” was never defined in the publications reviewed.
5. Few studies reference International Regulatory Guidelines for pharmaceutical development (e.g., ICH, EG, or FDA guidelines). Very few studies were performed in compliance with current regulatory guidelines. Most studies cited various animal use guidelines (specified animal treatment/handling guidelines).

A previous review (Christian and Brent, 2001) of the developmental toxicology of caffeine in animals and humans identified a No Effect Level (NOEL) of approximately 30 mg/kg/day in rodents, the reproductive NOEL to be approximately 80 to 120 mg/kg/day and the teratogenic NOEL as 80 to 100 mg/kg/day based on the following studies (Knoche and Konig, 1964; Palm et al., 1978; Aeschbbacher et al., 1980; Nolen, 1981; Nagasawa and Sakurai, 1986; Pollard et al., 1987; Purves and Sullivan, 1993). The 2001 publication essentially addressed the question of human teratogenicity of caffeine. The publication cautioned that although pregnant women who do not smoke or drink alcohol and who consume moderate amounts of caffeine (<5–6 mg/kg/day spread throughout the day) do not have an increase in any reproductive risks, individuals who consume large amounts of caffeine are at greater risk of being a smoker and of drinking alcoholic beverages to excess. Such an individual may have an increased risk of reproductive problems for other associated issues that have not yet been recognized as important reproductive and developmental toxic agents or behaviors.

If mammalian animal studies are to be utilized to estimate human risks, the oral route is the only appropriate route for evaluating human risks from exposure to caffeine in caffeinated beverages or naturally containing caffeinated drinks, food, or medication. The majority of animal caffeine studies did not use the oral route. However, analyses of all animal studies were performed regardless of the caffeine formulations, vehicles, route of administration, doses, or stages of pregnancy when exposure occurred. All recent animal toxicology publications were reviewed for relevance. Only those that included treatment during pregnancy, or the early postnatal period in rats, when the brain is similar in development to that of human fetuses, are included in this review. These publications are included by species and publication date in Supplemental Table 1, and Tables 6 and 7.

Unfortunately, the better designed and more comprehensive animal studies were performed before 2000. Palm et al. (1978) exposed Sprague–Dawley female rats before pregnancy and throughout pregnancy to 12.5, 25, or 50% brewed coffee in their drinking water, which was equivalent to 9, 19, or 38 mg/kg/day of caffeine. Even at the highest exposure there was no difference in the number of resorptions, litter size, fetal weight of sex ratio, or the offspring when compared to the control litters. On the 38th post partum day the animals that had been allowed to litter were comparable to the controls with regard to litter size, viable young, birth weight, and pup weight at 38 days. These results were in agreement with other investigators (Aeschbbacher et al., 1980; Nagasawa and Sakurai, 1986). Even relatively high exposures of caffeine or coffee in the water supply had minimal effects on birth weight, pup weight, and perinatal mortality in other studies (Knoche and Konig, 1964; Nolen, 1981; Pollard et al., 1987). Exposures of caffeine up to 60 mg/kg/day in rats and 74 mg/kg /day in mice did not alter the number of resorptions, conceptions, litter size, or births (Aeschbbacher et al., 1980; Nagasawa and Sakurai, 1986; Pollard et al., 1987).

The FDA commissioned an “in-house” study using pregnant Osborne–Mendel rats that were administered caffeine by gavage from 0 to 19 days of pregnancy with 0, 6, 12, 40, 80, or 125 mg/kg of caffeine with each intubation (Collins et al., 1981). The highest dose was maternally toxic as evidenced by the fact that 6 of the 50 pregnant rats in the 125mg/kg group died. CMs were increased in the two groups with the highest exposure. Ectrodactyly occurred in 28.5% of the fetuses in the 125 mg/kg group. The NOEL for CMs for caffeine was determined to be 40mg/kg/day. Many investigators had results that were similar to the FDA study (Bertrand et al., 1965; Leuschner and Schverdtfeger, 1969; Bertrand et al., 1970; Ikeda et al., 1982; Smith et al., 1987). It is important to emphasize that gavage or tube installation feeding will have a much lower teratogenic NOEL than when caffeine is placed in the water or food supply. In fact, in many studies investigators were unable to produce CMs by adding large amounts of caffeine to the water or food supply (Leuschner and Schverdtfeger, 1969; Gilbert and Pistey, 1973; Collins et al., 1983; Smith et al., 1987). Some investigators were able to produce malformations using caffeine in the water supply; however, it required an exposure of 330 mg/kg/day (Fuji and Nishimura, 1972).

Collins et al. (1981) demonstrated that a single oral gavage exposure of 80 mg/kg of caffeine was teratogenic, but 205 mg/kg/day in the water supply was not teratogenic. Stillbirths and miscarriages were observed with increased frequency among the offspring of macaque monkeys treated during pregnancy with caffeine in a dose equivalent to 5 to 7 or 12 to 17 cups of coffee per day (Gilbert et al., 1988). The cause for the stillbirths was not apparent at necropsy; no malformations were seen. Body weight of the male but not the female infants of treated monkeys were reduced (Gilbert and Rice, 1991).

An increased frequency of malformations, especially of the limbs and palate, has been observed among the offspring of rats or mice treated with caffeine during pregnancy in doses equivalent to human consumption of 40 or more cups of coffee daily (Purves and Sulliva, 1993; Nehlig and Debry, 1994). Fetal death, growth retardation, and skeletal variations are often seen in these animal experiments after maternal treatment with very high doses of caffeine during pregnancy. In one study an increased frequency of cleft palate was observed among the offspring of rats given the equivalent of 19 cups of coffee a day during pregnancy (Palm et al., 1978). An increased rate of cardiac defects was observed among the offspring of rats treated during pregnancy with the equivalent of 15 or more cups of coffee per day in another study (Matsuoka et al., 1987). Most investigations do not report an increased frequency of malformations among the offspring of rodents treated during pregnancy with caffeine in similar or somewhat greater doses (Purves and Sullivan, 1993; Nehlig and Debry, 1994; Christian and Brent, 2001). Doses and methods of caffeine administration that are teratogenic in animal studies generally cause maternal toxicity or death as well, and equivalent human doses would also be highly toxic or lethal.

Persistent behavioral and physiological alterations have been observed in some studies among the offspring of rats and mice treated during pregnancy with caffeine in doses equivalent to 10 to 60 cups of coffee a day (Nehlig and Debry, 1994). Behavioral alterations have also been observed among the offspring of monkeys born to mothers treated during pregnancy with caffeine in doses equivalent to 5 to 15 cups of coffee per day (Rice and Gilbert, 1990; Gilbert and Rice, 1994). The relevance of these observations to the risks in infants born to women who drink large amounts of caffeinated beverages during pregnancy is unknown.

High doses of caffeine influence the teratogenic activity of many other agents in animal studies (Nehlig and Debry, 1994; Sivak, 1994). Co-administration of caffeine often enhances the teratogenic action of other agents, but in some instances there is no interaction and in others, caffeine exhibits a protective effect. The relevance of these findings to humans is uncertain.

There are animal experiments that do assist in the evaluation of the human risks of caffeine exposure during pregnancy (Tables 8 and 9).

### Extrapolation of the Caffeine Animal Studies for Human Risk Assessment (Supplemental Table 1, and Tables 6–8)

Purves and Sullivan (1993) classified caffeine's teratogenic effect as a “peak blood level effect” and not an “area under the curve effect.” This is important because it emphasizes the importance of the method of administration in designing animal studies that are designed to evaluate the reproductive and developmental risks of caffeine in human populations. The peak exposure plasma level in animal models that is necessary to result in teratogenesis is equal to or >60 µg/ml (Elmazar et al., 1982; Ikeda et al., 1982; Smith et al., 1987; Sullivan et al., 1987) (Table 10).

**Table 10:** Magnitude of Congenital Malformation, Spontaneous Abortion and Growth Retardation Risks From Exposure to Caffeine During Pregnancy

Effect	Risk	Quality of the data
Congenital malformations	Unlikely	Good
Spontaneous abortion	Minimal	Fair to good
Fetal growth retardation	Unlikely	Fair to good

*Conclusion*: Consumption of caffeinated beverages during pregnancy is unlikely to increase the risk of congenital malformations and growth retardation; and poses a minimal risk for miscarriage, possibly at very, very high caffeine exposures. These risk estimates include the evaluation of inconsistent epidemiology studies and the utilization of animal (mammalian) reproductive studies.

The results of properly planned animal studies can be helpful in solving some of the dilemmas created by inconsistent findings in epidemiological studies. An animal study reported in 1960 first focused our attention to the potential developmental effects of caffeine. However, the exposure reported by Nishimura and Nakai (1960) was from intraperitoneal injections of 250 mg/kg in the mouse, an extremely high dose that would result in a blood plasma level that could never be attained from consuming caffeine containing products in food or beverages. More recent animal studies have demonstrated that depending upon the method of administration and species, the developmental NOEL in rodents is approximately 30 mg/kg/day; the teratogenic NOEL is 80 to 100 mg/kg/day, and the reproductive NOEL approximately 80 to 120 mg/kg/day (Nash and Persaud, 1988; Nolen, 1989; Stavric, 1992; Dlugosz and Bracken, 1992).

Purves and Sullivan (1993) agreed with the information previously cited by the FDA, since their conclusions are in basic agreement with the FDA position (1986). However, Purves and Sullivan (1993) evaluated the pharmacokinetics of caffeine more extensively, which is important to estimate the risk. The cited studies and comments convincingly demonstrate that the route of administration (bolus vs. administrating in drinking water or diet) and the timing of treatment during pregnancy (or development) are related to the serum blood levels attained in the specific species tested. As a result, this review indicates that such factors must be considered in any risk assessment process for caffeine, because under normal conditions of consumption, humans cannot attain serum blood levels comparable to those associated with the threshold for adverse effects from caffeine exposure in rats (Tables 8 and 9).

Although apparent differences exist because of the duration of administration, the study by Collins et al*.* (1983), in which caffeine was dissolved in drinking water, and the previous study described by Nolen (1981), in which caffeine was provided as brewed or instant coffee in drinking water, have remarkable similarities in the mode of caffeine administration (oral, drinking water) and the effects produced. Both these studies were conducted using adequate numbers of animals and well-defined protocols.

The relevance of the mode of exposure to resultant toxic effects was also confirmed by Smith et al. (1987). In this study Wistar rats were given 10 or 100 mg/kg/day of caffeine on p.c.ds. 6 to 20, either as bolus oral doses (once daily), or as four 2.5 or 25 mg/kg doses given at three-hour intervals. Maternal body weight and feed consumption were reduced in both groups given total doses of 100 mg/kg of caffeine and in the group given 25 mg/kg of caffeine four times/day. Developmental effects in these groups included dose-related decreases in fetal weight, placental weight, crown-rump length and skeletal ossification. Major abnormalities, principally ectrodactyly, occurred only in the group given the bolus 100 mg/kg dose, confirming the observations of Collins et al. (1983).

Colomina et al. (2001) exposed mice to caffeine (30 mg/kg) and aspirin (ASA). (250 mg/kg by gavage on the 9th post conception day.) There was no significant maternal or developmental toxicity in this group of animals and offspring. The studies also included stressful restraint. However, the exposure and the stress in the mouse studies cannot be utilized to determine human developmental risks, especially since the developmental results were minimal and the exposure equivalency in the human is unknown.

Evereklioghi et al. (2003, 2004) administered caffeine i.p. to Wistar rats on post conception days 9 to 21. There were four groups: 0, 25, 50, and 100 mg/kg/day. There was no maternal toxicity but there were seven fetal deaths in two dams in the 100 mg/day group. The investigators attributed the embryonic deaths to the i.p. injections of a high dose of caffeine. Histopathologic lens opacities were noted in the 100 mg/kg group. The investigators were unable to determine the human risk for cataracts from those studies.

Leon et al. (2002, 2005a, b) exposed Wistar rats to caffeine in drinking water from day 2 until delivery. The estimated exposure was 83.2 mg/kg/day. The authors hypothesized that caffeine and theophylline could have harmful effects on the developing fetal brain. Based on their findings they hypothesized that caffeine and theophylline may be associated with potentially harmful effects on the developing fetal brain.

Lutz and Beck (2000) administered 1.0, 2.5, and 5.0 mg/kg of cadmium subcutaneously on post conception days 9 to 12 in C57 BL/6 JBK mice. They were simultaneously administered zero or 50 mg/kg of caffeine subcutaneously. The teratogenic effects of cadmium were ameliorated by the caffeine administration. Litter size, fetal weight, fetal mortality, and dam weight were not affected by the co-treatment of caffeine.

Saadani-Makki et al. (2004) exposed pregnant Sprague–Dawley rats to 0.02% caffeine in their drinking water (postconception days of exposure not mentioned). The estimated caffeine consumption was 49.8 mg/kg/day. In utero exposure resulted in an increase in birth rate. There was also evidence for involvement of adrenergic A1 systems by the occurrence of respiratory perturbation in newborns. There was no discussion of human risk assessment of caffeine exposure based on these studies.

The critique of the animal studies may appear to negate their usefulness in estimating human reproductive and developmental risks. This conclusion may be due to the many animal studies utilizing parenteral or bolus administration of caffeine. The smaller percentage of animal studies that utilized the administration of caffeine in the food or drinking water has yielded important information summarized in Table 10. It indicates that the NOEL for teratogenesis necessitates a plasma level of caffeine >60 µg/ml. This is unattainable without pregnant women ingesting large quantities of caffeine. For example, 10 cups of coffee over a period of 8 to 10 hr (1,000 mg of caffeine) would never be able to reach a plasma level of 60 µg/ml.

## PHARMACOKINETICS

### Cross-Species Similarities in Metabolism

One reason that animal models are useful in the study of caffeine is that the pharmacokinetics of caffeine may be similar to humans in some animal species. In both animals and humans, oral administration of caffeine results in its rapid absorption, with peak plasma levels attained within 3 to 120 min (Perves and Sullivan, 1993). The absorption rates also increase with increased dosages in both humans and animals, and there is no significant first-pass effect, although absorption and the intestinal milieu do affect absorption, differing slightly in timing of distribution, but otherwise comparable in attained blood levels. Tomimatsu et al. (2007) described caffeine as hydrophobic and rapidly passing through all biological membranes, including the blood–brain and placental barriers in sheep. Absorption from the gastrointestinal tract is rapid in adult humans, with attainment of maximum caffeine concentrations within 15 to 60 min after oral ingestion (for dosages of 5 to 8 mg/kg, the plasma concentrations equaling 8 to 10 µg/ml) Supplemental Table 8. Once absorption occurs, caffeine is rapidly distributed in body water, equilibrating between blood and tissues, including the embryo/fetus, as well as the brain and testes. It is also rapidly distributed to the breast milk. Caffeine in human breast milk contains approximately 75% of the plasma level, depending upon the maternal dosage (3.2–8.6 µg/ml of caffeine is found in human breast milk and 0.7–7.0 µg/ml in rat milk). Consumption of caffeine in the milk results in only 1% of the maternal intake being consumed by human infants and 2% of the maternal intake consumed by rat pups (Perves and Sullivan, 1993).

Pregnancy alters the metabolism of caffeine, which, under normal conditions, is rapidly metabolically eliminated. Caffeine's retention is increased during pregnancy in humans, late human fetuses, and neonates, with a half-life varying from 80 to 100 hr. Presumably this increase in retention is the result of deficient P-450 enzymes in the fetus and neonate. Human metabolism of caffeine reaches adult parameters after approximately 7 months of age, but the half-life can be affected by inducing agents. For example, the half-life in smokers is approximately half of that in nonsmokers (Christian and Brent, 2001).

The characterization of the enzymatic process of caffeine metabolism was also explored by Buters et al. (1996), who investigated the involvement of CYP1A2 metabolizing enzymes in the pharmacokinetics and metabolism of caffeine using mice lacking its expression (CYP1A2^−/−^). The mice were intraperitoneally administered 2 mg/kg of caffeine, a dosage that was reported to be equivalent to that of a human drinking one cup of coffee. The half-life of caffeine elimination from blood was seven times longer, AUC was increased eight times, and clearance was consequently eight times longer in these animals than in wild-type mice. Other P450 enzymes were not affected and the clinical pathology evaluations of the liver and kidney were unaffected. These data indicate that the clearance (elimination) of caffeine in wild-type mice is primarily determined by CYP1A2. Because human and mouse CYP1A2 resemble each other in cDNA-derived amino acid sequence, these data also suggest that humans have a similar elimination pattern.

Derkenne et al. (2005) confirmed the conclusions of previous investigators that mouse or human CYP1A2 is the predominant enzyme for theophylline metabolism. Seven blood samples were taken at intervals from 5 to 400 min after IP injection of 8 mg/kg theophylline in mice. Replacing mouse CYP1A2 (− / −) with a functional human CYP1A2 gene restored the ability to metabolize theophylline, and the metabolism changed to a human profile. Comparing the hCYP1A1_A2 Cyp1a2 (− / −) and wild-type mice with published clinical studies revealed that theophylline clearance to be approximately 5 × and 12 ×, respectively, greater than that reported in humans, which is due to the well-known fact that mice clear drugs more rapidly than humans. Metabolism of caffeine varies remarkably among species and within the same species, and it is highly dependent on variables such as sex, age, and pregnancy status. In human newborns, the plasma half-life of caffeine is 4 days, while in young children and teenagers (6–13 years old), the plasma half-life is 2.3 hr. In adult humans, the half-life averages 2-6 hr in healthy nonsmokers, but it is prolonged in pregnant women to 10 to 20 hr. In rats, a half-life of 2.12 hr is reported for 8-week-old Sprague–Dawley male rats given one oral dosage of 4 mg/kg of caffeine. The major metabolite in humans is paraxanthine, or 1,7-dimethylxanthine. In rats, the major metabolite is 1, 3, 7-diaminouracil, or 6-amino-5-[*N*-formylmethylaminol]-1,3-dimethyluracil. Caffeine is demethylated in both rats and humans to three dimethylxanthenes (theophylline, theobromine, and paraxanthine), which suggests that rats are an appropriate model for use in risk assessment for humans.

The differences in caffeine and paraxanthine metabolism between human and murine CYP1A2 in liver microsomes were also explored by Labedzki et al. (2002). Results of the in vitro studies confirmed the important role of CYP1A2 in both murine and human metabolism of caffeine, despite formation of 1, 3, 7-trimethylurate as an in vitro “artifact” in both human and murine microsomal preparations. Both human and murine CYP1A2 enzymes have close similarities in the primary metabolic steps of caffeine. However, paraxanthine in vivo was not metabolized by murine CYP1A2 to a relevant extent, which is in contrast to the human situation. Also, results of this study confirmed the known reported inhibitory effects of the quinolones, norfloxacin, and pefloxacin on human CYP1A2, while in murine hepatic microsomes, quinolones did not exert an inhibition of caffeine 3-demethylation. The authors concluded that murine models are important for understanding the metabolism of xenobiotics in humans, but that extrapolation of data may be inaccurate in certain cases, such as in cases where compounds have low affinity ligands to CYP1A2. Therefore, interspecies comparison may be required before the use of mouse models to predict toxicity and/or pharmacological activity in humans. However, the metabolic patterns in rats are more closely related to the human.

### Effect of Caffeine on the Neonate

The capability to adequately metabolize xenobiotics are greatly reduced in neonatal or premature infants and animals due to an inadequately developed hepatic enzyme system, and often it is difficult to determine exact medicinal dosages during this age. In humans, intravenous theophylline is frequently administered to premature neonates during the first several days to reduce apnea, although there has been little emphasis in the literature on the pharmacokinetics in this segment of the population. Two clinical trials on this subject are presented below to describe some of the pharmacokinetic parameters.

The clearance rate (CL) and volume of distribution (V) of theophylline were studied by du Preez et al. (1999) in 105 apneic premature neonates (mean weight: 1.3 kg; age: 1.1 days) receiving intravenous loading dosages of 4 to 7.7 mg/kg aminophylline. Maintenance dosages ranged from 1.4 to 6 mg/kg/day in 2 to 4 divided doses. Data were analyzed using the nonlinear mixed effects model (NONMEM), and a one-compartment model with first-order elimination. The study differed from other cited premature neonatal references in that it was conducted in South Africa on all-black babies that had a 92% incidence of respiratory distress syndrome, and the described PK related only to the first few postnatal days. Low CL values were recorded (0.0084 and 0.056 l/hr/kg, respectively, for babies with and without oxygen support), while values of ≥0.012 l/hr/kg have been cited by other investigators. As a result of the low CL, long half-lives (54 and 76 hr, respectively, for babies with and without oxygen support) were reported. The calculated value for V was 0.63 l/kg. Variability in both CL and V were high, and it was concluded that theophylline PK is highly variable in neonates because physiologic parameters are changing rapidly and theophylline clearance and urinary metabolite patterns apparently do not reach stable adult values until 55 weeks postconception.

Urinary output was also evaluated in 19 premature infants aged 4.5 ± 4.0 days before and after a 20-min loading solution of aminophylline (6 mg/kg), which was followed by a maintenance therapy of 2 mg/kg every 12 hr (Mazkereth et al., 1997). The infants had a mean gestational age of 31.1 ± 2.8 weeks and a birth weight of 1481 ± 454 g. Marked diuresis occurred immediately after the loading dose, and the ratio of urinary output to water intake increased from 0.58 ± 0.36 to 1.19 ± 0.65. Fractional excretion of sodium and potassium increased, and urinary calcium and uric acid excretion was also enhanced. Tubular reabsorption of phosphorus was not affected. These effects were no longer evident after 24 hr, despite aminophylline maintenance therapy. The authors concluded that the aminophylline acted directly on tubular reabsorptive functions of the nephron. Neonatal patients afflicted with hyperbilirubinemia may also gain some benefit from a neonatal rat model that could be used to evaluate new therapeutic agents for this disease. Induction of cytochrome P450 1A (CYP1A) may be a valuable therapeutic modality for reducing the hyperbilirubinemia of infants with Crigler–Najjar syndrome type I (CNS-I), a severe form of congenital jaundice. To evaluate inducers of CYP1A, a novel assay was established by Jorritsma et al. (2000), based on the comparison of the type of urinary pattern of caffeine metabolites in rats when 10 mg/kg of 1-Me-14C-caffeine is injected intraperitoneally before and 48 hr after injection of a potential CYP1A inducer, such as 5,6-benzoflavone (BNF). The inducing effect of BNF on CYP1A activity was confirmed by the urinary pattern of caffeine metabolites in Wistar rats and was paralleled by a decrease in plasma bilirubin in male **jj** Gunn rats.

It is interesting to note that in conjunction with the above study, a selective and sensitive reverse-phase liquid chromatographic method was developed by Schrader et al. (1999) for the simultaneous analysis of [1-methyl-14C] caffeine and its eight major radiolabel-led metabolites in rat urine. Separation of the complex mixture of metabolites was achieved by gradient elution with a dual solvent system using an endcapped C18 reverse-phase column, which, in contrast to commonly used C18 reverse-phase columns, also allows the separation of the two isomers of 6-amino-5-(*N*-formylmethylamino)-1,3-dimethyluracil (1,3,7-DAU), a metabolite of quantitative importance predominantly occurring in rats.

### Impact of Various Factors on Altering the Pharmacokinetics of Caffeine

The effect of gender on the pharmacokinetics of caffeine (5 mg/kg, intravenously) was explored in 10 male and 10 female Holstein cattle during the ages 1, 2, 4, 6, 8, 12, and 18 months (Janus and Antoszek, 2000). The findings were compared to the results in other species, including humans. The volume of distribution (*V*) decreased significantly with age, as it does in pigs and humans; results were similar in males and females. A steady, significant decrease in mean residence time (MRT) also occurred in both sexes, although the MRT was significantly shorter in females after 8 months of age. Significant decreases over time also occur in dogs, pigs, and humans because caffeine clearance depends principally on intrinsic hepatic clearance. Total plasma clearance (Cl) of caffeine increased by nearly 100% between the first and 18th month of life (from 0.80 to 1.55 ml/min/kg in males; from 0.84 to 1.80 ml/min/kg in females). Similar changes occur in dogs and humans; the change is due to inadequate development of the hepatic microsomal enzyme system in the neonatal period. It was concluded that clear-cut sex differences in MRT and Cl occurred in cattle over eight months in age, the females being the more active metabolizers.

In a similar manner, Janus et al. (2001) investigated the effects of short-term (4 days) starvation or water deprivation on the pharmacokinetics of caffeine (5 mg/kg, intravenously) in three groups of ten 24- to 25-day-old Holstein calves. An automated enzyme-multiplied immunoassay technique was used to determine plasma caffeine concentration just before the administration of caffeine and four days later at the end of the deprivation period. Results from the caffeine study indicated that four days of starvation or water deprivation was associated with significant increases in MRT and Total Plasma Clearance (Clt) of 20 to 30%. *V* was slightly (not significantly) decreased. It was concluded that the results from this study were similar to the findings reported in sheep, horses, laboratory animals, and humans, and indicate that starvation and water deprivation lead to a general inhibition of the hepatic P450 enzyme system and may impair the elimination of drugs that undergo metabolism by these enzymes.

Pelissier-Alicot et al. (2002) investigated the effects of caffeine on the daily rhythms of heart rate, body temperature, locomotor activity, and caffeine pharmacokinetics (PK) in 10-week-old male Wistar rats in relation to time-of-day. The study was divided into three 7-day phases: a control period, a treatment period, and a recovery period. During the treatment period, 25 mg/kg of caffeine was administered subcutaneously to groups of rats (four rats/group) at 8:00 AM in the morning, and to other groups at 8:00 PM in the evening. Blood for PK parameters was drawn at periodic intervals of 0.25 to 24 hr postinjection on the 7th day of treatment. Telemetry was used in similarly treated rats to obtain pharmacodynamics data. Morning administration of caffeine suppressed locomotor activity and modified the diastolic–systolic amplitudes of heart rate and body temperature; evening administration did not alter locomotion, but altered the blood pressure elevations, amplitudes, and acrophases of the three rhythms, indicating a chronopharmacologic effect. PK data revealed that the area-under-the-curve (AUC) was significantly lower in rats medicated in the evening, compared to medication in the morning, due to an increase in total plasma clearance and volume of distribution. However, there was no significant time of administration-dependent difference in *C*_max_, *T*_max_, or half-life.

The influence of hepatic regeneration after partial hepatectomy (removal of median and lateral lobes) on theophylline (Th) pharmacokinetics in groups of five adult male Wistar rats was studied by Maza et al. (2001). At 12 and 24 hr and 3, 6, and 15 days after partial hepatectomy, Th was administered intravenously as a single dosage of 6 mg/kg, and plasma concentrations were determined at periodic intervals. Liver weights and clinical pathology parameters were also determined. Liver mass at the respective dates above were: 3.8, 5.0, 6.5, 7.1, and 9.4 g, compared to 12.1 g in nonhepatectomized rats. Liver function tests were increased significantly at 12 and 24 hr. Initial Th concentrations and volume at steady state varied during regeneration. The control elimination half-life of 4.30 ± 1.37 hr notably increased after hepatectomy (7.27 ± 1.38 hr), and then decreased with time to 5.17 ± 0.87 hr at 15 days. The increase in elimination half-life led to a decrease in mean residence time during the period of regeneration; however, the intrinsic clearance hardly varied.

### Appropriate Use of Animal Studies for Assessing Human Risk

Although many metabolic and kinetic factors appear similar in rats and humans, only clinical studies in humans and intact animal pharmacokinetic studies in animals can be used to extrapolate risks from animal species to humans. There are few or no data regarding blood levels attained or the comparability of dosages administered. One of the most important considerations regarding comparability of blood levels is that humans consume caffeine over a period of time, rather than as a bolus dosage, and certainly not from an intraperitoneal injection. Humans consuming a 1 to 2 mg/kg dosage of caffeine attain a blood concentration of 1 to 2 µg/ml; a 3 to 5 mg/kg intake of caffeine results in a 5 µg/ml serum concentration. Thus, a 1 mg/kg intake produces a 1 µg/ml blood concentration over the range humans are likely to consume, fitting first-order kinetics for human metabolism of caffeine. The kinetics in rats is dose-dependent and zero order, indicating a saturable process, particularly at high dosages (Christian and Brent, 2001) (Tables 8 and 9).

Many animal studies in the previous review (Christian and Brent, 2001) and in this current review were conducted using bolus gavage dosages, rather than exposure over a period of time as the result of administration in the drinking water or diet. Such differences in the route of exposure often confound interpretation of data and results in inappropriate identification of the NOEL (no observable effect level). Most comparisons are made on the basis of mg/kg dosages, rather than attained blood levels, that are generally considered more useful in cross-species extrapolation, but which are rarely identified in human studies. For example, pregnant rats that were administered caffeine by gavage or via the drinking water for the first 11 days of pregnancy and then administered an 80 mg/kg dosage of radiolabeled caffeine on days 12 to 15 of gestation had blood serum concentrations of caffeine that were much greater after gavage dosage (60–63 µg/ml) than after drinking water exposure (0.10–5.74 µg/ml). However, the drinking water levels were more variable because of the remarkable variability in timing and consumption of drinking water. The half-life of an 80 mg/kg dosage of caffeine in pregnant rats in this study was approximately 1.7 to 2.6 hr (Christian and Brent, 2001) (Tables 8 and 9). When two bolus gavage dosage of caffeine, 5 and 25 mg/kg, were administered to Wistar pregnant rats, apparent enzyme saturation resulted in nonlinear kinetics at the higher dosage only, resulting in an increased half-life and/or an increased distribution phase. However, mean peak plasma concentrations in nonpregnant and pregnant gestation day 20 rats and in the placenta, amniotic fluid, and fetal blood were linear at approximately equivalent times for both dosages. At 24 hr after the 25 mg/kg dosage, plasma concentrations of caffeine were 2 µmol/l (0.4 µg/ml) and 20 µmol/l (4 µg/ml) in nonpregnant and pregnant rats, respectively, and the half-life was significantly longer in pregnant (8.9 hr) than in nonpregnant (3.8 hr) rats at the 5 mg/kg dosage but increased at the 25 mg/kg/day dosage, indicating saturation (Christian and Brent, 2001) (Tables 8 and 9). When given intravenously to pregnant sheep, as described by Tomimatsu et al. (2007), maternal intravenous administration of 3.5 mg/kg of caffeine resulted in a maternal plasma caffeine concentration of 5 µg/ml and fetal caffeine concentrations in excess of 80% of maternal concentration. Other authors cited that the metabolism of caffeine differs between rats and humans, with the half-life much shorter in rats. Using a correction factor, Tanaka et al. (1983) demonstrated that a dosage of 70 mg/kg/day ingested by pregnant rats is equivalent to a dosage of approximately 30 mg/kg/day for humans. Thus, Bodineau et al. (2003) considered a 49 mg/kg/day dosage of caffeine in drinking water to pregnant rats to be in the moderate range for a human model although all other authors consider this a high exposure. Newborns exposed to caffeine in utero exhibited apnea postnatally.

In toto, these toxicokinetic experiments show that

1. Serum and/or plasma concentrations of caffeine are much higher in rats after gavage treatment than after sipping treatment or continuous intravenous infusion;
2. Pregnancy alters pharmacokinetics in both humans and rats, and
3. The changes may be dose-dependent and species-specific.

Yet, pharmacokinetic studies with caffeine can serve a very useful purpose, especially when it is used as a biomarker for the estimation of xenobiotic biotransformation and possible hepatotoxicity. An example of such an investigation was conducted in adult mice (BALB/c mice) by Kolarovic et al. (1999). The test article was enflurane, a fluorinated volatile anesthetic, administered by inhalation in either anesthetic or subanesthetic doses, with/without prior intraperitoneal injection of 1 g/kg ethanol. Two control groups were administered only ethanol or saline. Anesthetic exposure occurred for 6 hr/day for 5 days. On the 6th day, half the mice were injected intraperitoneally with 20 mg/kg caffeine and 8-hr urine samples were collected for caffeine metabolite assay; remaining mice were used to determine liver function and cytochrome P450 analysis. Liver function tests were all normal, but liver P450 levels were higher in the group treated with enflurane and ethanol, compared to other groups. Excretion of caffeine and its metabolites was different among the groups. Quantities of caffeine metabolites that are predominantly metabolized by CYP-4502E1 were higher in urine of enflurane-treated mice, while quantities of caffeine metabolites predominantly metabolized by CYP-4501A2 were significantly lower than in controls. Control values for the CYP-4501A2 enzymes were: 1,7-dimethyl uric acid (1,7-U) = 4.155 ± 1.956; 1,3,7-threemethyl uric acid (1,3,7-U) = 6.314 ± 2.992. It was concluded that use of caffeine as a biomarker is a highly sensitive test for estimating xenobiotic transformation and possible hepatotoxicity.

### Plasma Levels Versus Organ Exposure

Plasma levels are not always indicative of exposure of a specific organ. The disposition of caffeine and its metabolites were evaluated in brains from adult and fetal rats on p.c.d. 20 after a single maternal dosage of 5 or 25 mg/kg of caffeine. Fetal and adult caffeine AUC values did not differ between brain and plasma at either dosage. However, the three primary metabolites of caffeine in rats accumulated in the fetal brain at both dosages, resulting in a 3-fold increase in brain metabolite exposure compared with fetal circulatory levels (Christian and Brent, 2001).

### Caffeine Studies Relevant to Teratogenicity or SA (Pregnancy Loss)

Previous FDA (1980) conclusions and those described by Christian and Brent (2001) appear to provide sufficient precaution regarding consumption of caffeine, that is, that moderate consumption of caffeine (which was defined as ≤5–6 mg/kg/day) is unlikely to increase the risk of SA. These conclusions also appear to apply to the two additional human studies summarized below that were included in the present literature search conducted in 2008.

In a case–control study of 73 women with, and 141 women without SA, Fenster et al. (1998) determined the activity of the three principal caffeine-metabolizing enzymes (P4501A2, xanthine oxidase, and *N*-acetyltransferase) by measuring the levels of caffeine metabolites in urine. Caffeine was entered as a categorical variable in models with the following levels of caffeine consumption: no caffeine level; 1 to 150 mg/day (<2.5 mg/kg in a 60-kg woman); and >150 mg/day. Results established no association between caffeine consumption, caffeine metabolism, and risk of SA. However, due to small sample size, the study was not able to reliably estimate the risk for recurrent abortion in relation to caffeine consumption and the indices of enzyme activity.

Possible adverse effects of caffeine on pregnancy were also investigated by Klebanoff et al. (2002). They tested 2,515 women to determine whether third-trimester maternal serum concentration of paraxanthine, caffeine's primary metabolite, is associated with the delivery of a small-for-gestational age infant (birth weight of <10th percentile for gender gestational age and ethnicity), and whether the magnitude of the association is affected by smoking. The subjects were selected from women who enrolled in the Collaborative Perinatal Project at 12 sites in the U.S.A. The mean serum paraxanthine concentration was greater in women who gave birth to small-for-gestational age infants (754 ng/ml) than to “normally” grown infants (653 ng/ml, *p* = 0.02). However, the linear trend for increasing serum paraxanthine concentration to be associated with increasing risk of small-for-gestational age birth was confined to women who also smoked (*p* = 0.03). There was no association between paraxanthine and fetal growth in nonsmokers (*p* = 0.48).

The Frog Embryo Teratogenesis Assay—Xenopus (FETAX) was used to test the 13 metabolites, including theophylline, paraxanthine, and a synthetic methylxanthine analogue (Fort et al., 1998). Frog embryos were exposed to two concentrations of each test article, with or without a metabolic activation system. Assay results indicated that the fetotoxic potencies of each of the di- and monomethylxantine metabolites were similar to that of caffeine. None of the caffeine metabolites tested was found to be significantly more potent than caffeine itself in the FETAX assay.

### Modulation of Teratogenic Properties of Other Agents

It is well known that low dosages of caffeine can modulate the teratogenic effects of other agents in animal studies. As summarized in Lutz and Beck (2000), defects produced by ionizing radiation, chemical carcinogens, and pharmaceuticals, including anticonvulsants, all have been shown to be potentiated by nonteratogenic dosages of caffeine. In contrast, 5-azacytidine-induced digital defects in mice were suppressed by post-treatment with caffeine. Treatment with caffeine also reduced the teratogenicity of urethan, ethylnitrosourea, and 4-nitroquinoline-1-oxide. Although environmental exposures to Cadmium (Cd) are not considered to be a human teratogen, it has been shown to be teratogenic in rats, hamsters, and mice, with the predominant malformation being right-sided forelimb ectrodactyly in mice. This malformation has also been reported in mice after exposure to carbonic anhydrase inhibitors; acetazolamide, ethoxzolamide, and dichlorphenamide. The results of a study by Lutz and Beck (2000) provide evidence that a nonteratogenic dosage of caffeine (50 mg/kg, s.c.) can ameliorate Cd-induced forelimb ectrodactyly in this Cd-sensitive mouse strain (C57BL/6JBK mice) injected intraperitoneally with 0, 1.00, 2.50, or 5.0 mg/kg of Cd on post conception day 9 and examined on post conception day 18 for ectrodactyly and other gross morphological malformations.

### Caffeine Interaction with Stress

A series of manuscripts were produced by researchers at the University of Seville, Spain and the University of Picardie Jules Verne, France (Bodineau et al., 2003; Saadani-Makki et al., 2004; Gaytan et al., 2006) regarding the potential effects of caffeine and other xyanthines as the result of their binding with adenosine receptors and their potential effect on respiration. Again, these studies were conducted because caffeine is used therapeutically to normalize breathing in apnea-affected infants. The authors stated that premature infants may be exposed to relatively high serum concentration of caffeine (10–15 µg/ml) for up to 8 weeks of treatment. They referenced Shi et al. (1993) who demonstrated that chronic caffeine exposure alters the density of adenosine, adrenergic, cholinergic, GABA, and serotonin receptors and calcium channels in the mouse brain, resulting in a reduction in the fetal cerebral weight. They also indicate that sustained maternal caffeine intake induces harmful physiologic effects on human newborns, including respiratory perturbations, citing a case report (Khanna and Somani, 1984) of a woman reported to have consumed 24 cups of coffee per day during pregnancy, with a newborn who experienced apnea episodes attributed to methylxanthine withdrawal.

The first study by Bodineau et al. (2003) was conducted using the drinking water route (calculated consumed caffeine dosage = 49 ± 4 mg/kg/day). A subsequent study by the same group (Saadani-Makki et al., 2004) used tissues from the generated pups and evaluated brainstem–spinal cord preparations isolated from these newborn rats. In both studies, the authors noted an increase in pup weight, without any consideration for the mean number of pups per litter. Both these observations should be considered unrelated to caffeine [the increase in newborn weight (7.7 g) in the caffeine exposed group versus the control (6.7 g) was most probably the result of the fewer pups in the caffeine group (10.9 pups) versus the control (13.8 pups), a finding reflecting the relatively few litters evaluated (eight per group) and the normal variability in litter sizes]. No historical data were provided.

In the Bodineau et al. (2003) study, the consequences of in utero caffeine exposure on respiratory output in normoxic and hypoxic conditions and related changes of Fos (binding protein involved in transcription regulation) expression were evaluated. The study was conducted using brainstem–spinal cord preparations isolated from newborn rats. Sprague–Dawley rats (control and caffeine groups = 8/group) were given water or 0.02% caffeine in water, with intake evaluated daily, presumably from conception until parturition, because the caffeine was removed after parturition. The experiments were conducted on brainstem–spinal cord preparations isolated from 37 control and 35 caffeine group rats. The authors claimed to know the exact dosage consumed (50.4 ml/day control, 62.3 ml/day—caffeine) with the consumption of 49 ± 4 mg/kg/day, estimated according to drinking fluid intake. The body weight was increased and litter size of the newborn caffeine group rats was reduced, compared with the control group. However, based on the standard deviation of the caffeine group, it is probable that one litter was affected (data were not provided; and the authors did not identify statistical significance). A later study (Saadani-Makki et al., 2004), using tissues from the same animals, further evaluated involvement of the adenosinergic A1 systems in the occurrence of respiratory changes in newborns after in utero caffeine exposure and the importance of the rostral pons in adenosinergic A1 modulation in respiratory control. As before, exposure was during pregnancy, via maternal drinking water, and caffeine fluid intake was estimated at 49.8 mg/kg/day, based on drinking fluid intake, a toxic level. The authors concluded that their work brought evidence of the involvement of the adenosinergic A1 systems in the occurrence of apnea in newborn infants after in utero caffeine exposure.

Further studies by this group (Gaytan et al., 2006) evaluated postnatal exposure to caffeine on the pattern of adenosine A1 receptor distribution in respiration-related nuclei of the rat brainstem. They evaluated the ontogeny of the adenosine A1 receptor system in the brainstem of the newborn rat after postnatal treatment with caffeine. This study identified that the previously reported results, with the main difference between control and caffeine administered rats being the transient increase (on postnatal day 6 only) in the parabrachial and Kõlliker–Fuse nuclei, which are classically associated with the adenosine A1 receptor system. The authors concluded that the role of caffeine in decreasing the incidence of neonatal respiratory disturbances may be due to earlier than normal development of the adenosinergic system in the brain.

There was another group of publications originating in Spain regarding the potential interactions of caffeine and stress during pregnancy in mice (Colomina et al., 2001; Albina et al., 2002). In the manuscript by Colomina et al. (2001), a single oral dosage of caffeine or aspirin on p.c.d 9 was given to mice orally exposed to toxic levels of caffeine (30 mg/kg/day), aspirin (250 mg/kg), or a combination of caffeine and aspirin (30 and 250 mg/kg, respectively). Three additional groups were given the same doses and restrained for 14 hr. The pregnant mice were restrained 2 hr/day on p.c.ds 0 to 18 by placing them in methacrylate cylindrical holders and keeping them in a prone position with the paws immobilized with elastic adhesive tape, a procedure the authors previously reported to produce stress in pregnant mice (Colomina et al., 1995; Scialli et al., 1995; Colomina et al., 1999). Other mice were given toxic dosages of caffeine by gavage at 30, 60, and 120 mg/kg/day on GDs 0 to 18, and another group was administered the same dosages of caffeine immediately followed by restraint stress for 2 hr/day on the same days (Colomina et al., 1999). No caffeine levels were recorded. Although the authors do not identify maternal toxicity, it is noteworthy that the weekly intervals measured for body weights are inappropriate (drug treatments and restraint occurred on one day; the intervals are evaluated for three or four days). Maternal toxicity was evident, with reductions or frank weight losses in body weight and feed consumption measurements. Regarding caffeine, these effects were most severe for the three groups of interest (restraint, 30 mg/kg caffeine and combined 30 mg/kg of caffeine and 14 hr of restraint), on p.c.ds. 9 to 11. Of these three groups, the effects were most severe for the combined caffeine and stress group. The 30 mg/kg plus restraint group also had an increase in postimplantation loss, including dead fetuses and late resorptions. An increase in early resorptions was seen in the restraint alone group, but the group with both restraint and 30 mg/kg of caffeine were increased compared with the restraint alone group. As would be expected, there was an increase in reduced ossification in the restraint group alone, the 30 mg/kg caffeine alone, and the combined caffeine and stress group. There was no increase in malformations in any group. The authors considered there to be some clinical relevance for the data because real life involves multiple simultaneous exposure to many chemicals. However, the duration of oral exposure to aspirin and caffeine on gestational day 9 in this study is not analogous to the type of stress experienced by pregnant women who drink coffee and take aspirin. Interspecies differences and pharmacokinetics and bioavailability are both important consideration.

Albina et al. (2002) reported a study by Nehling and Debry (1994) in which daily consumption of caffeine ranged from 203 to 283 mg, or 2.7 to 4.0 mg/kg/day of caffeine in adults (equivalent to 3.38–4.72 mg/kg for a 60 kg person). Albina et al. (2002) also refer to the FDA 1980 recommendation that pregnant women limit caffeine consumption to less than 400 mg/day (6.7 mg/kg/day for a 60 kg human), based on animal studies (FDA, 1980). These authors report that 30 mg/kg/day of caffeine administered with maternal stress is an effect level (they did not report that stress alone was an effect level). Nonetheless, the authors recommended that women under notable stress during pregnancy should reduce caffeine ingestion to reasonable levels; for example, a dosage of 10 mg/kg/day. For 60-kg women, 10 mg/kg/day would be a daily ingestion of 600 mg, or four cups of strong coffee or eight cups of weak coffee.

### Interaction of Caffeine as a Pharmaceutical

A series of studies in rats was conducted by Burdan and his colleagues at the Experimental Teratology Unit of the Human Anatomy Department of the Medical University School in Lublin, Poland. The initial objective was to evaluate the effects of caffeine on skeletal development, when administered by gavage during gestation (Burdan et al., 2000). The later studies were designed to evaluate the effects of over-the-counter preparations of various mixtures of propyphenazone, caffeine, and paracetamol, with the purpose of determining liver toxicity (Burdan et al., 2001) and the prenatal risk of COX inhibitors administered with or without caffeine (Burdan, 2002, 2003, 2004). The studies were conducted in general conformance with evaluations performed for testing pharmaceuticals, but used fewer rats than are usually utilized in studies designed for regulatory use (generally 15 per group, rather than the recommended 16–20 litters), an abbreviated treatment period p.c.ds. 8 to 14, rather than the current usual interval, gestation days 7 to 17. As a result, the exposure period differs by one day from many studies published for regulatory use. Nevertheless, the manuscripts are well documented and easily interpreted. All the findings regarding caffeine's maternally and developmentally toxic dosages do not indicate new concerns, even in combination with the interacting medications.

Burdan et al. (2000) did not observe adverse maternal or developmental effects at caffeine dosages up to 70 mg/kg administered on p.c.ds. 8 to 14, which is unusual. The Burdan et al. (2001) study showed that caffeine is toxic to the liver only at dosages greater than those tested in this study (the highest dosage of caffeine tested was 70 mg/kg/day), and when given for a prolonged period. The dosages tested in this study were mixtures prepared in 5:3:1 ratio (acetaminophen, isopropylantipryine, and caffeine), with the caffeine dosages at 0.7, 7, and 70 mg/kg/day. Although the authors concluded that the administration of the mixture to nonpregnant rats at the maximum dosage tested in this study only slightly impaired liver function, hepatotoxic effects were observed in pregnant female rats at the high dosage. Thus, they also concluded that the pregnant rat's liver was more vulnerable than the nonpregnant rat's to the tested materials, although they cautioned that the studies were difficult to extrapolate to human exposure.

The next series of studies of combined drugs in over-the-counter products evaluated acetaminophen, isopropylantipyrine, and caffeine (Burdan, 2002). There were 29 control rats and 15 to 19 per group in those administered the caffeine mixture. Caffeine was given by gavage at 0.7, 7.0, or 70 mg/kg, in combination with the other drugs (acetaminophen:isopropylantipyrine:caffeine 5:3:1 ratio [A:I:C]). The authors concluded that this mixture of acetaminophen, isopropylantipyrine, and caffeine administered in a constant proportion of 5:3:1 for the entire second week of pregnancy was not teratogenic in rats but was maternally toxic at the mid and high dosages (35:21.4:7 and 350:214:70 mg/kg, respectively), and was embryotoxic only at the high dosage (350:214:70 mg/kg, respectively).

Burdan (2003) then administered dosages of 3.5:0.7, 35.0:7.0, and 350:70 mg/kg/day of paracetamol:caffeine, respectively, on p.c.ds. 8 to 14. All dosages were maternally toxic, producing reduced maternal weight gain and liver weight. The mid and high dosages also reduced kidney weights, observations that were attributable to paracetamol. At the maternally toxic mid and high dosages, reduced fetal body weight/growth and placental weight occurred, previously described reversible effects of gavage dosages of caffeine, but there was no increase in fetal malformations.

Finally, Burdan (2004) administered maternal dosages of 2.1:0.7, 21:7, or 210:70 mg/kg prophyphenazone:caffeine on p.c.ds. 8 to 14. The only evidence of maternal toxicity was decreased liver weight at the high dosage. Fetal body weight was reduced in groups given the middle (21:7 mg/kg prophyphenazone:caffeine) and high (210:70 mg/kg prophyphenazone:caffeine) dosages of the propyphenazone:caffeine mixtures and the middle dosage of the propyphenazone:paracetamol mixture. The effects on fetal body weight were not dose-dependent, possibly because of the increase in resorption that also occurred at the high dosage. These results are similar to the previously described parallel studies in which all three compounds were given separately or in a mixture. Dose-dependent liver injury was seen in dams given propyphenazone and caffeine and the mixture with all three ingredients, with a hepatotoxic effect and decrease in maternal body weight in the middle and high dosage groups. The authors concluded that co-administration of propyphenazone and caffeine or propyphenazone and paracetamol caused growth retardation but no teratogenic effects and that the results supported the prenatal safety of low dosages of caffeine.

### In Vitro Study on Placental Gene Expression

Nomura et al. (2004) studied whether caffeine alters gene expressions in human cytotrophoblast-like cell line, Be Wo, using cDNA microarray technology. Tissues were obtained from pregnant rats fed a 20% protein diet or the same 20% protein diet supplemented with caffeine 2 mg/100 g body weight (20 mg/kg) from day 1 (fertilization) until day 20 of gestation, when the placentas were removed by Caesarean-section. Placental blood flow decrease has the potential to lead to intrauterine growth retardation. The present findings demonstrated that caffeine caused a decreased level of Bcl-2 expression in a human trophoblast cell line and placentas removed from caffeine-administered pregnant rats. The exposure of 20 mg/kg is very high and it would be problematic to apply these findings to human pregnancies.

### Caffeine Studies Regarding Adenosine Receptor Interaction and Adenosine Effects

As noted in some of the studies previously discussed, caffeine interacts with the adenosine receptor, and it is the most widely known adenosine receptor antagonist. The biochemical mechanism underlying the effects of caffeine is the blockade of adenosine receptors, which is an antagonist for adenosine modulation. Although adenosine receptor interaction wth caffeine may not result in teratogenicity, caffeine may affect neuronal growth and neuron interconnections during gestation and the neonatal period. It would be important to determine the NOAEL for deleterious effects on neuronal growth and neuron synapse formation.

Caffeine modulation of adenosine receptor and ontogeny was tested in the following studies. Snyder (1984) provided an extensive review of adenosine as a potential mediator of the behavioral effects of xanthines, approximately 20 years after it was identified that phosphodiesterase was an enzyme that degraded cyclic AMP (Sutherland and Rall, 1958; Butcher and Sutherland, 1962; Salmi et al., 2007). According to Iglesias et al. (2006), adenosine, a nucleoside, is widely distributed in the peripheral and central nervous systems and acts through G-protein coupled receptors. Four types of receptors have been identified: A1, A2A, A2B, and A3. A1 and A3 receptors inhibit adenylyl cyclase activity through Gi protein. A2A and A2B receptors act by stimulating adenylyl cyclase activity through Gs protein. A1 and A2A receptors have a greater affinity with adenosine and are blockaded by caffeine. Adenosine, working through the A1 receptors, inhibits glutamate release, thus acting as a neuromodulator and neuroprotector. Snyder (1984) also noted that phosphodiesterase was inhibited by the xanthines, including caffeine and theophylline, and that via this mechanism, xanthines could elevate cyclic AMP levels. However, to substantially inhibit phosphodiesterase, millimolar concentrations of caffeine were required, approximately 100 times the levels of caffeine found in the human brain after ingestion of typical dosages in humans. In addition, it was noted that some inhibitors of phosphodiesterase were 100 to 1,000 times more potent than caffeine but without behavioral effects.

Adenosine has many effects, including dilation of blood vessels, especially in the coronary and cerebral circulation, inhibition of platelet aggregation, and inhibition of hormone-induced lipolysis. It also has a variety of actions on central neurons, usually inhibiting spontaneous neuronal firing (Phillis and Wu, 1981; Stone, 1981). Adenosine inhibition of the release of excitatory neurotransmitters is the predominant presynaptic activity, although postsynaptic effects are also present. Many studies were conducted testing the hypothesis that in utero exposure altered adenosine receptors and their activities, including postnatal functional activity in the brain and heart. All the studies appear to have been performed at dosages that either were toxic, were reversible in effect, or not sufficiently well documented for use in human risk assessment.

The first biochemical analysis of adenosine receptor activity was by Sattin and Rall (1970) who demonstrated that adenosine can increase the accumulation of cyclic AMP in brain slices without conversion of adenosine to cyclic AMP, an action on extracellular receptors. The effects of adenosine on the enzyme adenylate cyclase, which synthesizes cyclic AMP, revealed two distinct subtypes of adenosine receptors, designated A1 and A2 (van Calker et al., 1979; Burnstock and Brown, 1981; Londos et al., 1981). Depending upon the system, adenosine increases or decreases adenylate cyclase activity, with the enhancing actions occurring at micromolar concentrations via A2 receptors. Nanomolar concentrations of adenosine cause the A1 receptors to inhibit adenylate cyclase activity. Marked sterospecific effects of phenylisopropyladenosine (PIA) occurs at the A1 receptors. L-PIA is remarkably more potent than D-PIA, although the two isomers are relatively similar in effect at the A2 receptors. Most xanthines have similar potencies blocking both A1 and A2 receptors.

Direct binding studies have demonstrated that in all species studied, adenosine receptors labeled with [3H]DPX, a xanthine derivative, binding showed that nanomolar potency was present for adenosine derivatives and sterospecificity for PIA isomers. However, binding studies identified heterogeneity of adenosine receptors beyond the A1 and A2 distinction. Another xanthine derivative (DPX) was about 250 times more potent in competing for [3H]CHA sites in calf than in guinea pig and human brain. As summarized by von Borstel and Wurtman (1984), considerable evidence has been accumulated that competitive antagonism at cell surface adenosine receptors may be the most important molecular action for methylxanthines, including caffeine. Administration to animals can produce sedation, bradycardia, hypotension, hypothermia, and attenuation of the response of the heart, vascular, and adipose tissue to sympathetic stimulation and are generally opposite to those produced by caffeine or theophylline alone. Methylxanthines competitively antagonize these and other adenosine actions at concentrations similar to those found in plasma after consumption of one to three cups of coffee (5–30 µM) (Rall, 1980).

A series of new manuscripts identified in this review describe studies designed to evaluate the effect of caffeine on adenosine receptor ontogeny. One group of investigators (Adén et al., 2000) identified that administration of caffeine at dosages resembling those consumed by humans does not significantly influence the development of receptors known or believed to be affected by caffeine. The results, described below, in contrast to other publications, indicate that caffeine can modify adenosine receptors and/or behavior. However, it is unclear what dosages were used or what postnatal blood levels of caffeine were attained. Adén et al. (2000) reported that maternal caffeine intake has minor effects on the adenosine receptor ontogeny in the rat brain. Caffeine was provided in the drinking water given to pregnant rats, beginning on p.c.d. 2 and continuing throughout gestation and postnatal life of the offspring. Although the authors noted that only a low dosage of caffeine was administered, estimated to be up to 3 cups of coffee/day, or what a woman might drink during pregnancy, it must be noted that mg/kg/day consumed dosages vary throughout gestation and lactation. This is further confounded by the pup's consumption of the maternal drinking water, which contained caffeine. They reported that low-dosage caffeine-exposure during gestation and postnatal life had minor effects on the development of adenosine A1 and A21 receptors and GABAA receptors in the rat brain.

Other studies were often designed to evaluate whether caffeine affected excitotoxic brain lesions in mice, because it is often given to human pre-term newborns. Bahi et al. (2001) examined the effects of caffeine on neonatal excitotoxic lesions of the periventricular white matter. This study was designed to mimic caffeine exposure of human preterm infants in neonatal intensive care units. Most of this study is inappropriate for inclusion in this review because it addresses postnatal evaluations, rather than in utero exposure. It has been included because it had two sets of experiments, one performed postnatally and the other with in utero exposure, unfortunately by the intraperitoneal route (5 mg/kg caffeine citrate administered IP to 3 pregnant dams on p.c.ds. 8–18 and another group injected IP with 12.5 mg/kg caffeine on p.c.ds. 8–11). Although no mechanism was shown, it appeared that caffeine had a neuroprotective effect in mice.

An interaction study in knock-out mice was performed by Björklund et al. (2007) to investigate whether the response of the adenosine receptor system to a low perinatal exposure to methylmercury (MeHg) would be altered by caffeine treatment or eliminated by genetic modification (A1R and A2AR knock-out mice). Pregnant mice were administered 1 µM MeHg and/or 0.3 g/l caffeine (>30 mg/kg) in the drinking water. The consequences of MeHg toxicity during gestation and lactation were reduced by adenosine A1 and A2a receptor inactivation, either by genetic deletion or treatment with their antagonist, caffeine. This work also showed a protective effect of a high caffeine dosage of (>30 mg/kg/day).

In a 2008 study, da Silva et al. evaluated maternal caffeine intake to determine whether it affected acetylcholinesterase in the hippocampus of neonatal rats. The control group was given tap water, and the caffeine group given 1.0 g/l caffeine diluted in tap water. Experiments were performed using 30 male and 30 female pups at 7, 14, and 21 days of age. Caffeine did not change the age-dependent increase of acetylcholinesterase activity or the age-dependent decrease of acetylcholinesterase expression. However, it resulted in a 42% increase in acetylcholinesterase activity, without changing the level of acetylcholinesterase mRNA transcripts in 21-day-old rats. These results further demonstrate the ability of maternal caffeine intake to interfere with cholinergic neurotransmission during brain development.

A series of studies conducted by investigators in Spain considered the effects of down regulation of Adenosine A1 receptors and other receptors in the brain and heart that are affected by caffeine (León et al., 2002, 2005a, b; Iglesias et al., 2006). They reported caffeine intake as 83.2 mg/kg/day (administered at 1 g/l in the drinking water from p.c.ds. 2 throughout pregnancy [sperm = gestation day 1]). The reported estimated dosage appears to be correct, because a 250 g rat would consume at least 20 ml/day of drinking water, although this value is somewhat low for a pregnant rat. These investigators considered this dosage equivalent to approximately 80 to 180 mg caffeine in a cup of coffee, or consumption of one cup of coffee by a pregnant woman. This calculation appears inappropriate because 180 mg consumed by a 60-kg human would be equivalent to only 3 mg/kg/day, much lower than the 83.2 mg/kg/day dosage consumed by the rats.

In the León et al. (2002) publication, it was reported that caffeine consumption during gestation caused down-regulation of adenosine A1 receptors in both the maternal and fetal brain. The later publications noted that it also inhibited A1 receptor function in the maternal rat brain and down regulation of metabotropic glutamate receptors in the brain from both mothers and fetuses (Leon et al., 2005a, b). The results of this study, evaluating isolated rat heart membranes, immunodetection of mGluR1, indicate down-regulation of different components of the mGlur I/PLC pathway in the maternal and fetal heart, and loss of receptor responsiveness in fetuses that can alter the physiological function of the heart, especially in fetal tissue mGluRs.

Iglesias et al. (2006) demonstrated that chronic intake of caffeine during gestation in rats down regulates metabotropic glutamate receptors in maternal and fetal rat heart. While most of the studies involve the interaction of caffeine with adenosine receptors (Sutherland and Rall, 1958; Butcher and Sutherland, 1962; Snyder, 1984; Iglesia et al., 2006) caffeine also interacts with adrenergic, cholinergic, GABA, and serotonin receptors as well as calcium channels (Shi et al., 1993).

### Cardiovascular Effects

Keller et al. (2007) provided an excellent review of cardiovascular development in which maternal exposure to hypoxic and bioactive chemicals, for example, caffeine, can rapidly impact embryonic/fetal cardiovascular function, growth, and outcome. No specific description of caffeine exposure in animals or humans was provided.

A study by Asadifar et al. (2005), while not relevant to toxicity produced as the result of in utero exposure of pregnant rats to caffeine, addresses the interaction of combined effects of caffeine and malnutrition on Cu content in the neonatal rat heart.The results of this study, in which neonates were administered a normal diet with 20% protein, 20% protein supplemented with caffeine (4 mg/100 g BW) or 6% protein diet (malnourished) or 6% protein supplemented with caffeine (4 mg/100 g BW) from birth to postnatal day 10 were surprising and not what was expected. The caffeine level was considered comparable to consumption of a heavy coffee drinker, defined as 4 cups of coffee containing an average of 100 mg of caffeine and an average body weight of 50 kg (400 mg/50 kg = 8 mg/kg). The results show that malnutrition did not impair mitochondria, and that although it was expected that caffeine exposure would aggravate their Cu status, the results were the opposite of the hypothesis. Caffeine exposure affected Cu status more in the normally nourished animals than in the malnourished animals, an apparent protective effect.

Momoi et al. (2008) further evaluated maternal and embryonic cardiovascular function in CD-1 mice administered 10 mg/kg/day caffeine subcutaneously on p.c.ds. 9.5 to 18.5 of a 21-day pregnancy period (this information appears in error, because mice have an 18-day pregnancy). Blood levels were not reported, so it is not possible to extrapolate to human exposure, although the authors considered the exposure to be equivalent to modest daily maternal exposure. (It should be noted that the caffeine was administered by injection rather than by oral administration in the diet, so it is unlikely that this exposure was comaparable to human caffeine exposures.) No maternal toxicity or increase in embryo resorption was observed. At p.c.d. 18.5, crown-rump length, forelimb length, and wet body weight of caffeine-treated embryos were smaller than the control embryos. The main findings of the study were reported as: (1) modest daily maternal caffeine exposure altered regional developing embryonic arterial blood flow and induced intrauterine growth retardation without impacting maternal CV function or weight gain; (2) caffeine at peak maternal serum concentration transiently reduced embryonic carotid arterial flow to a greater extent than dorsal (and descending) aortic or umbilical arterial flow; (3) maternal adenosine A2A receptor blockade reproduced the embryonic hemodynamic effects of maternal caffeine exposure; and (4) adenosine A2A receptor gene expression in the uterus and developing embryo were down regulated by maternal caffeine exposure. The authors considered the 10 mg/kg dosage of caffeine to be a modest maternal caffeine dosage. They also stated that maternal caffeine effects in a mouse model may not reflect human effects, and concluded that modest daily maternal caffeine exposure may have a negative effect on embryonic CV function and overall embryonic growth, possibly mediated by adenosine A2A receptor blockade.

Another study in near-term fetal sheep (Tomimatsu et al., 2007) was performed to test the hypothesis that maternal caffeine administration does not significantly alter fetal cerebral oxygenation. The authors considered the dosage comparable to one that may be consumed by pregnant women in daily life. The pregnant ewes and their fetuses were instrumented at post conception day 125 ± 3 (term ∼ 145 days). A total of 800 mg of caffeine citrate (400 mg of caffeine, reported as approximately 8 mg/kg, that is, equivalent to 2–3 cups of coffee) into the maternal inferior vena cava over 30 min. Fetal arterial and sagittal sinus blood samples and maternal arterial samples were collected every 10 to 15 min and analyzed for blood gases, hemoglobin concentration, oxyhemoglobin saturation, and calculated O^2^ content. Maternal parameters were unaffected. Fetal arterial blood gas values at 5, 30, and 40 min after the 30-min maternal infusion of caffeine were also not significantly affected. However, sagittal sinus O^2^ content and oxyhemoglobin saturation were significantly decreased in fetuses, although neither fetal heart rate nor mean arterial blood pressure were significantly changed. After 30 min of maternal caffeine infusion, fetal LD-CBF decreased slightly (− 7%). Fetal cortical PO2 decreased, and arterial to sagittal sinus O^2^, content difference, cerebral fractional O^2^ extraction, and CMRO each increased 20 to 30% above baseline. Authors concluded that the results of their study showed findings that would suggest a small compromise in cerebral oxygenation occurred without affecting overall fetal systemic oxygenation. Further studies are needed to determine whether there are any related clinical findings.

### Encephalization

There is some evidence that caffeine accelerates encephalization (development of the cerebral cortex). A publication by Sahir et al. (2000) describes a potential model for studying human holoprosencephaly. These investigators confirmed their previous in vitro work (Marret et al., 1997), describing effects of caffeine on early encephalization. In this work, they evaluated i.p. dosages of caffeine (12.5, 25, or 50 mg/kg) administered on p.c.ds. 8, 9, and 10 and then scored the embryos for encephalization. Increased encephalization was noted on embryonic day 10 at all caffeine dosages, as compared with controls, and on embryonic day 9 at the 25 and 50 mg/kg/caffeine dosages. Normalization of brain anatomy and histology was noted within a few days after caffeine was discontinued, observations in agreement with the plasticity of the developing brain. The dosages tested were high and administered by an inappropriate route (12.5–50 mg/kg/day, i.p.) compared to human consumption (2.7–4 mg/kg/orally over a day). The results do not appear to represent a concern for humans, although the model may be useful for the evaluation of telencephalic vesicle formation. A later publication by this group of investigators (Sahir et al., 2001) used similar methodology, i.p. injection once daily of mice on p.c.ds. 8.5 to 10.5 with 25 mg/kg/day of caffeine or either of one of two inhibitors of cAMP-dependent protein kinase (PKA). The dams were subsequently killed on p.c.d. 10.5, and the embryos were evaluated for histology and various tests for gene expression and sequencing. As cited previously, embryos treated with 25 mg/kg caffeine had significant acceleration of telencephalic vesicle formation, compared with control embryos. The authors concluded that the study showed involvement of PKA activity in caffeine-induced acceleration of encephalization, however, at relatively high exposures.

### Potential Model for the Production of Cataracts

Two publications (Evereklioglu et al., 2003, 2004) reported results from the same set of rats. The Evereklioglu et al. (2003) study was designed to identify whether histopathology could reveal changes in the neonatal rat cornea resulting from caffeine exposure during pregnancy. The Evereklioglu et al. (2004) study focuses on the examination of the crystalline lenses in neonatal rats. Unfortunately, the study methodology was not well reported, and some tabular errors are evident, which preclude appropriate independent interpretation of the results.

Wistar pregnant rats and the i.p. route were used to treat a control and three dosage groups. As the result of the use of a route that is inappropriate for extrapolation to human exposure (i.p. dosages of 25, 50, and 100 mg/kg/day were administered between p.c.ds. 9–21), exposure relevant to human exposure comparisons cannot be made. A fifth group was given caffeine via gavage at a toxic dosage of 50 mg/kg/day. Dams delivered normally (generally on p.c.ds 20–21). Half of the newborn rats per litter were decapitated at postnatal day 1, and the eyes were examined. The remaining litters were raised with their biological mothers and sacrificed and decapitated at postnatal day 30 for eye evaluation. Pups were evaluated on postnatal days 1 or 30, and the eyes enucleated for corneal histopathology. Although the investigators refer to “pup” and “groups” and statistical analysis of these, it is somewhat unclear how this occurred because it appears that only one randomly selected eye (right eye) was evaluated. Thus, it appears that each litter and dosage group is represented by only one pup and one eye at each time interval.

No maternal toxicity was reported; however, 7 pups were reported as “miscarried” by 2 dams in 100 mg/kg/day caffeine Group 4 (high dosage), because rats do not generally abort but resorb their dead conceptuses. These “late fetal deaths” were probably either a sequela of IP injection and/or apparent premature delivery associated with incorrect identification of the mating date. Pup body weights were slightly decreased in all groups in a dose-dependent pattern. It is unclear whether the number of litters evaluated included the aborted litter at birth, or whether these litters were included with those with pups evaluated on postnatal day 30. Table 1 in the Everekiloglu et al. (2003) publication appears to incorrectly report the number of pups per litter as the mean number of pups per litter at birth. The authors concluded dosages of 50 mg/kg/day and higher affected development of the cornea, particularly postnatal at 100 mg/kg/day. Interestingly, macroscopic changes were not observed in any corneas on postnatal day 30.

In the later publication regarding effects in the same rats (Everekilioglu et al., 2004), the ultimate objective was to establish a model for the study of cataract development, specifically, to investigate histologically the influence of maternal caffeine exposure during pregnancy on the development of the crystalline lenses in neonatal rats. In the control and 25 mg/kg/day dosage groups, both slit-lamp biomicroscopic and histopathologic examination of the crystalline lenses revealed normal findings. Histological examination of the 50 and 100 mg/kg/day IP groups and the 50 mg/kg/day PO group had findings suggesting cataractogenesis, including eosinophilic degeneration, lens fiber cell swelling and liquefaction, central lens fibers with retained nuclei, and prominent epithelial cells lining the posterior lens capsule behind the equator. Some lenses in the intraperitoneal 100 mg/kg/day group had immature cataract on slit-lamp biomicroscopic examination at postnatal day 30. The authors concluded that excessive maternal caffeine exposure during pregnancy had cataractogenic effects. As previously reported, no macroscopic ocular abnormalities were observed in control or experimental groups at birth, and the i.p. administration of high doses of caffeine prevents the ability to perform a valid risk assessment in humans.

## EVALUATION, DISCUSSION, AND RISK ANALYSIS

The method of evaluation that has been utilized in past publications (Brent, 1978; Shepard, 1986, 1994; Brent 1986a, b, 1995a, b, 1997, 1999, 2003, 2005; Brent and Beckman, 1990; Christian and Brent, 2001) will be utilized in this publication and is described in Table 1. It consists of evaluating the (1) epidemiological studies, (2) determining whether secular trend analysis is an appropriate technique to utilize, (3) animal studies, (4) evaluating the available pharmacokinetic and toxicokinetic information, (5) Testing the biological plausibility of any reported findings or hypotheses based on (a) MOA (mechanism of action), (b) receptor agonistic and antagonistic effects, (c) enzymatic stimulation or suppression, and (d) basic reproductive and developmental teratology principles.

### Epidemiological Studies

Epidemiological studies are the most important area of research for evaluating human risks from environmental exposures. It is most helpful if the epidemiology study results are in agreement (consistent). We know that cohort studies are the most likely to be accurate with regard to identifying causal associations; however, for rare events you need very large exposed populations to study. They are costly and difficult because the studies need large numbers of cases and controls. Case–control studies can be performed with smaller numbers of cases and controls and are more likely to find associations that are not causal. Consistent findings of increased risks or no increased risks strengthen the believability of the results. None of the new caffeine epidemiological studies of the 21st Century included complete pharmacokinetic data in their research protocol. The studies continued to measure exposure by cups per day, per week, or even per month of caffeine containing beverages. Some investigators hypothesized that slow metabolizers of caffeine may be at greater risk because the mother's serum levels of caffeine and caffeine metabolites would be higher and protracted. The studies of CYP1A2 activity's impact on the risk of SA were inconsistent and in some cases the results were the opposite of what was expected.

In this publication the epidemiological studies pertaining to SA, CMS, and fetal growth retardation were evaluated noting that appropriate animal studies can assist in the risk assessment analysis.

#### SA

Of all the reproductive and developmental events, SA is the most difficult to evaluate in epidemiological studies (Tables 2 and 3). The complexity of performing research involving the evaluation of whether a particular environmental agent is responsible for an increase in the prevalence of SA is discussed in detail in the SA section of this article. Furthermore, it will be obvious that many of the epidemiological studies failed to recognize the impact of the factors that alter the veracity of epidemiological studies dealing with SA, which are summarized in the earlier section. Seventeen caffeine epidemiological studies were reviewed that were concerned with SA. Almost all the studies reported no association in pregnant women consuming three or less cups of coffee per day. Eight of the studies were negative at all exposures that were studied. The epidemiology studies were not consistent in their conclusions. At high exposures it was difficult to eliminate confounding factors such as smoking, alcohol ingestion, decreased nutrition, the Susser effect, and many other confounding factors that may be associated with “excessive” caffeine ingestion. So the conclusion in this review was that caffeine ingestion at the usual or even very high exposures is an unlikely cause of SAs. Ten of the studies did not account for the impact of the “pregnancy signal”. Only one of the studies discussed the multiple etiologies of abortion and the complexity of performing SA research studies. Nor did these epidemiology studies cite the animal studies that examined embryonic and fetal resorptions that refuted the concept that caffeine is an abortifacient at the usual or even high exposures of caffeine in pregnant women.

#### Congenital malformations (CMs)

Each of the 11 recent CM epidemiological studies evaluated only one particular isolated birth defect and none of them focused on a syndrome of abnormalities that is usually associated with a teratogenic effect. Most teratogens do not produce a single isolated defect. Teratogens produce an identifiable syndrome of effects that are caused by the teratogen (Wilson and Brent, 1981; Beckman and Brent, 1986a; Brent, 1986b, 1994, 1995a, b, 1999, 2004, 2008; Shepard, 1986, 1994; Beckman et al., 1997; Cary et al., 2009) (Table 5).

The congenital malformation studies dealing with caffeine focused on the following individual malformations: cleft lip and palate, hypospadius, cardiac malformations, Down syndrome, anorectal atresia, kidney malformations, and cryptochidism. All were negative, except for the Schmidt et al. (2009) study. The Schmidt et al. (2009) study reported an association of caffeine ingestion with isolated NTDs. This study had serious errors and inconsistencies that are detailed in the earlier section. However, the protocols of all these studies did not utilize the basic principles of teratology in selecting the malformations to study or in analyzing the results (Table 5). It is clear from previous epidemiological studies and the animal studies that caffeine does not represent an increased teratogenic risk. Even much higher exposures than 3 to 5 mg/kg/day are not teratogenic. Depending upon the method of administration and the species, animal studies have demonstrated that the developmental NOEL in rodents is approximately 30 mg/kg/day, the teratogenic NOEL is 80 to 100 mg/kg/day, and the reproductive NOEL approximately 80 to 120 mg/kg/day (Knoche and Konig, 1964; Aeschbbacher et al., 1980; Nolen, 1981; Nagasawa and Sakurai, 1986; Pollard et al., 1987; Purves and Sullivan, 1993).

The animal studies that utilized pharmacokinetics estimated the teratogenic plasma NOEL at 60 µg/ml, a level that would rarely, if ever, be reached from caffeine nutritional exposures in pregnant women (Tables 8 and 9). The malformations described in the animal studies at very high doses fit the description of vascular disruptive types of malformations (Nishimura and Nakai, 1960). However, in the epidemiological studies reporting malformations in a caffeine-exposed population, the malformations that were selected for the study were not of the vascular disruptive type and no caffeine teratogenic syndrome has been described (Wilson and Brent, 1981; Brent, 1986, 1994, 1999, 2004, 2008; Christian and Brent, 2001).

#### Fetal weight reduction

The 17 epidemiology studies dealing with the risk of fetal growth retardation from caffeine exposure during pregnancy did not consistently report that growth retardation was present in these studies. Four of the studies reported growth retardation with ingestions above 300 mg/day and eight of the studies did not. In eight of the studies the “pregnancy signal” was not included in the evaluation. The decrease in birth weight was very small and had minimal clinical significance. In some of the positive studies many of the possible confounding factors that could be etiologically related to growth retardation (tobacco, alcohol, nutritional problems, maternal disease states, maternal behavioral, or psychiatric problems) were not evaluated (Smith, 1947). While large doses of caffeine administered parentally or by bolus to rats or mice can result in fetal growth retardation, the studies utilizing caffeine in drinking water or the food supply (Aeschenbacher et al., 1980; Nagasawa and Sakurai, 1986; Pollard et al., 1987) did not result in rat or mouse fetal growth retardation at exposures that are much higher than are likely to occur in the human.

None of the epidemiological studies focused on the difference between reparable and nonreparable growth retardation. Many causes of growth retardation are permanent and the infant never recovers from the in utero growth retardation (chromosome abnormalities, in utero teratogenic and nonteratogenic infections, many teratogenic drugs and chemicals and some forms of nutritional deprivation). Reparable growth retardation following placental insufficiency or from pregnancies whose mothers smoked have much better prognosis than the fetuses that never recover completely from the in utero growth retardation. None of these studies determined whether the subjects in their studies recouped or recovered from their decreased growth in utero.

Inconsistent findings of growth retardation and igonoring the importance of the pregnancy signal diminish the value of the conclusions of the epidemiology studies. Thus, fetal growth retardation is unlikely to be caused by the usual human exposures of caffeine.

### Secular Trend Analysis

When a significant segment of the population is exposed to a drug or chemical, changes in population exposure may be associated with an increase or decrease in the incidence of reproductive or teratogenic effects. This can happen when a very popular drug is introduced or withdrawn from the market. Secular trend analysis cannot be utilized if only a very small segment of the population is exposed. Caffeine exposure is so universal and difficult to monitor that it would be impossible to attribute changes in reproductive or developmental effects to changes in population caffeine exposure.

### Animal Developmental Toxicity Studies (Supplemental Table 1, and Tables 6–9)

When human epidemiological studies or a case series presumptively indicate that a cluster of malformations may be caused by a drug or chemical, an animal model may be developed that mimics the human developmental effect at clinically comparable exposures (Brent et al., 1986). There are over 50 proven human teratogens and for almost every teratogen scientists have been able to produce an animal model at exposues pharmacokinetically comparable to the human exposures (with the exception of infectious teratogens that primarily affect the human species) (Brent, 2004, 2008). Animal studies have demonstrated that the developmental NOEL in rodents is approximately 30 mg/kg/day; the teratogenic NOEL is 80 to 100 mg/kg/day, and the reproductive NOEL approximately 80 to 120 mg/kg/day (Persaud, 1988; Nolen, 1989; Nash and Stavric, 1992; Dlugosz and Bracken, 1992). These NOELs are derived from caffeine administration via bolus administration. The NOELs are much higher when the caffeine is administered in the food or water supply. Only eight (8) of the recent animal studies administered the caffeine in the drinking water or food. In those studies that estimated pregnancy loss, there was not an increased risk of pregnancy loss with exposures much higher than the 30 mg/kg. All the animal studies that evaluated the risk of congenital malformations found no increased risk with the usual or even the highest range of human caffeine exposure (Supplemental Table 1, and Tables 6–9). The smaller percentage of animal studies that utilized the administration of caffeine in the food or drinking water has yielded important information summarized in Table 10. It indicates that the NOEL for teratogenesis necessitates a plasma level of caffeine >60 µg/ml. This is unattainable without pregnant women ingesting very large quantities of caffeine. For example, 10 cups of coffee over a period of 8–10 hr (1,000 mg of caffeine) would never be able to reach a plasma level of 60 µg/ml. This is true for growth retardation and pregnancy loss as well.

### Pharmacokinetics

Some of the animal studies performed before 2000 have provided investigators with pharmacokinetic data that can be utilized for risk analysis (Knoche and Konig, 1964; Aeschbbacher et al., 1980; Nolen, 1981; Nagasawa and Sakurai, 1986; Pollard et al., 1987; Perves and Sullivan, 1993) (Table 10). One of the recommendations of the Christian and Brent (2001) “Teratogen Update” was that any future caffeine epidemiological studies should measure caffeine and caffeine metabolites as an important component of the study. Extensive information regarding the metabolism of caffeine is presented in this publication. The information should be useful for future investigations in the caffeine field, so that any future caffeine toxicology studies will have a significant pharmacokinetic component. The inconsistencies of previous studies are partly the result of not knowing the actual exposures of the participants. In Tables 8 and 9 are the animal and human pharmacokinetic data that are available for human risk assessment. These tables are the most important tables in this publication because they demonstrate that it is unlikely that a pregnant woman could ingest enough caffeine via her diet to result in fetal growth retardation, pregnancy loss, or congenital malformations.

### Biological Plausibility (Biological Common Sense)

#### Case reports and clusters

It is a common knowledge (a truism) that most teratogens have been discovered by an alert physician or scientist from clusters of patients with a group of similar malformations (Brent et al., 1986; Carey et al., 2009). An historical example is Gregg's observation of children in his ophthalmology practice with cataracts and associated malformations whose mothers had contracted Rubella during their pregnancy (Gregg, 1941). Case–control studies verified his observation as being correct. Another teratological truism is that a single case report of a drug exposure during pregnancy that resulted in a malformed child is rarely a causal relationship. Is a single case report ever useful? Bodineau et al. (2003) cited a case report of a newborn “intoxicated” by caffeine because the mother drank 24 cups of coffee/day during pregnancy.

The case report reads as follows (Khanna and Somani, 1984):

A male infant weighing 1,236 g was born to a 23-year-old Gravida 1, Para 0, white married woman at 27 weeks gestation. Amniotic fluid was leaking for 24 hr before delivery. The mother received 10% alcohol i.v. to attempt to stop the labor without success. She also received 16 mg of Dexamethasone i.v. 24 hr before delivery. The infant was spontaneously delivered vaginally. Apgar scores were 9 and 10. The infant developed respiratory distress and was administered 40% oxygen before being referred to a high-risk neonatal center. The gestational age was estimated to be 31 weeks at the neonatal center. He was diagnosed with transient tachypnia of the newborn. Cultures and electrolytes and the metabolic panel were all negative. Apnea of >20 sec was first noted at 4 days of age. On the 5th day, because of the apnea and the history of caffeine ingestion, a blood specimen was obtained for caffeine followed by caffeine administration (10 mg/kg), followed by 5 mg/kg every 12 hr. By the sixth day the apnea was no longer present. The serum caffeine concentration before the administration of caffeine was 40.3 µg/ml. The half-life of caffeine in a premature baby is estimated to be approximately 100 hr. It was estimated that at birth the infant had an estimated serum caffeine level of 80 µg/ml. On the 12th day postpartum, the serum caffeine concentration in the infant was 47.7 µg/ml.

No congenital malformations were detected and the infant's birth weight was normal for gestational age. No further problems were encountered and the baby was discharged at 43 days of age. A serum sample was obtained from the infant at the time of discharge and was 0.7 mg/ml. At the postdischarge follow-up at 6 months later, the child was growing and developing normally.

The history of maternal caffeine intake is interesting. During the pregnancy she was taking as much as 24 cups of coffee per day. About 5 days before delivery, she reduced her coffee at work to 5 to 6 cups of coffee per day. After delivery she was drinking 5 to 6 cups of coffee per day. A maternal serum caffeine level on the 10th postpartum day was 18.4 µg/ml. Unfortunately, we do not have a caffeine level when she was taking 24 cups of coffee/day.

How much information can you obtain from one clinical report? It is apparent that this case report is extremely valuable. When a subject ingests 3 to 5 cups of coffee/day, a 60-kg subject is exposed to 5 to 8 mg/kg, which results in a serum concentration of 8 to 10 µg/ml. These are not absolute figures. For example, Stavric (1988) states that when a human consumes a cup of coffee delivering a 1 to 2 mg/kg dosage of caffeine it results in a blood concentration of 1 to 2 µg/ml, while a 3 to 5 mg/kg intake leads to a 5 µg/ml concentration. The serum measurements in this case-report indicate that the infant may have received a massive caffeine exposure as a fetus. If the infant was exposed to a very high level of caffeine why was the infant not growth retarded or malformed? Most likely, because the caffeine levels did not reach 60 µg/ml and lower levels do not produce congenital malformations or growth retardation.

#### The importance of the “mechanism of action” (MOA)

The evidence that demonstrates that an environmental toxicant can produce reproductive or developmental effects in humans can be determined from the results of five areas of investigation (Table 1). Dose–response relationships in the reviewed epidemiology studies are primarily determined by estimates of exposures and there is meager data pertaining to the pharmacokinetics of caffeine and its metabolites. Since 2000, only four epidemiology studies reviewed in this article considered actual exposures. Even more surprising is the fact that none of the epidemiological studies discussed the mechanism by which caffeine can produce SAs, congenital malformations, stillbirths, prematurity, fetal growth retardation, or fertility problems. The mechanisms by which reproductive toxicants produce their effects are listed in Table 10. Only one of the listed mechanisms in Table 10 have the possibility of providing a mechanism for reproductive toxicity of caffeine and that is agonistic or antagonistic effect on the adenosine, adrenergic, cholinergic GABA, or serotonin receptors. The pharmacokinetic levels of caffeine from low and high exposures are not cytotoxic or mutagenic. Nor is there definite data indicating that it can affect development or reproduction by any of the other mechanisms listed in Table 10.

#### The importance of the “pregnancy signal”

The “pregnancy signal phenomenon” has been discussed in many obstetrical and epidemiology publications (Weigel and Weigel, 1989; Lawson et al., 2004). In the Lawson et al. study, the authors reported that the vast majority of nonsmoking coffee drinkers decreased or quit drinking coffee during the first trimester. In fact 65% reported a unique aversion to coffee. There was a 59% decrease in coffee consumption between the 4th and 6th week of gestation. The authors were of the opinion that a decrease in coffee consumption may be a signal for a healthy pregnancy and therefore can act as a confounder. In many of the epidemiological studies published between 2000 and 2010, including the SA studies, the pregnancy signal was not considered. This omission could invalidate the results and conclusions of these studies.

#### Fecundity and fertility studies

Preconception exposure of sperm or ova (eggs) to mutagenic drugs and chemicals have theoretical risks of producing chromosome abnormalities or point mutations in the developing germ cells. Since caffeine is not a potent mutagen or carcinogen, an increase in the mutagenic risks would appear to be very unlikely (Table 10). There is extensive evidence supporting the conclusion that even potent mutagens at low exposures have a very low risk of having a significant effect on the developing surviving fetuses at term or a mutagenic effect (chromosomal abnormalities and point mutations). At high exposures, mutagenic agents can reduce ova survival and produce severe chromosomal abnormalities that result in very early embryonic death. This scenario is the classic dominant lethal test. However, caffeine is unlikely to increase the risk of birth defects by this mechanism because even potent chemical mutagens and ionizing radiation exposure to animals and humans before conception do not cause a significant increase in the incidence of genetic disease or birth defects in the live offspring (Mulvihill et al., 1987; Ames and Gold, 1990; Neel and Lewis, 1990; Nygaard et al., 1991a, b; Autrup, 1993; Brent, 1994, 1999, 2007; Byrne, 1999; Neel, 1999; Boice et al., 2003; Winther et al., 2004).

## CONCLUDING REMARKS

After reviewing the 2000 to 2010 scientific epidemiology literature concerning the reproductive and developmental toxicology risks of caffeine, we conclude that major advances in the risk estimates have not been made and the confounding phenomena continue to be present in the present caffeine studies. An increase in pharmacokinetic studies has not occurred. We still do not know whether the increased risk estimates for some developmental and reproductive effects at higher exposures are due to caffeine or are due to other confounding factors. It appears that we should evaluate and continue to improve the animal studies to determine whether we can answer the many unanswered questions.

It may not be possible because of cost and invasiveness for epidemiological investigators to initiate pharmacokinetic studies to determine the actual caffeine exposure in the pregnant women exposed to caffeine that are being studied for reproductive and developmental effects. Further studies utilizing “cups” of tea, coffee, and colas will add little more to the understanding of caffeine “toxicity” from the plethora of studies that have been published.

The pharmacokinetics of caffeine and its metabolites was reviewed if only to demonstrate the complexity of evaluating caffeine's toxic effects without knowing the basic science of caffeine metabolism. Caffeine's main effect is on the central nervous system as a stimulant that interacts with the adenosine receptor and can also interact with adrenergic, cholinergic, GABA, or serotonin receptors, the implications of which are unknown.

1. In vivo animal caffeine studies should mimic human exposures, which is oral administration.
2. Second, every epidemiology study that is initiated should include recognition of the “pregnancy signal” as an important factor in determining the extent of reproductive and developmental risks in the population being studied.
3. Third, rarely has an investigator explained the MOA of caffeine. How does caffeine produce growth retardation, birth defects, SA, or premature births? Caffeine is not mutagenic, oncogenic, or cytotoxic at the usual human exposures. Agonism or antagonism of the adenosine receptor is unlikely to be related to developmental or reproductive toxic effects. It is interesting that scores of investigators are interested in the “toxic” effects of caffeine but not the mechanism to explain the toxic effects.
4. Planning and analyzing epidemiological studies by utilizing the principles of teratology would markedly improve the caffeine epidemiology studies (Table 5).

Our conclusion is that the dietary exposures of caffeine are not teratogenic or are directly responsible for an increased risk of SA or fetal growth retardation. Studies that involve very high exposures to caffeine are difficult to evaluate because of the many confounding factors that contribute to the risks that are not adequately evaluated; however, the animal studies indicate that even the highest human exposures in the epidemiological studies are unlikely to have reproductive and developmental effects (Table 10).

## Abbreviations

CDC: Centers for Disease Control
CL: plasma clearance
CMs: congenital malformations (birth defects)
CNS: central nervous system
Fos: binding protein involved in transcription regulation
FDA: Food and Drug Administration
GD: gestation day
HESI: Health Environmental Science Institute
HHS: Human Health Services
i.p.: intraperitoneal
ILSI: International Life Science Institute
IUGR: intrauterine growth retardation
MeHg: methyl mercury
MRT: mean residence time
MOA: mechanism of action
NOAEL: no adverse effect level
OTIS: Organization of Teratology Information Specialists
p.c.d.: post conception day (s)
PIA: phenylisopropyladenosine
s.c.: subcutaneous
SA: spontaneous abortion (miscarriage)
SGA: small for gestational age (less than the 10th percentile for gestational age)
TERIS: website http://apps.medical.washington.edu/teris/teris1a.aspx
Th: theophylline
V: volume distribution
WHO: World Health Organization
