# Supplementary material for: Evaluation of the Reproductive and Developmental Risks of Caffeine
Source: Birth Defects Res B Dev Reprod Toxicol. 2011 Apr;92(2):152–87. doi: 10.1002/bdrb.20288 (PMC3121964; doi:10.1002/bdrb.20288)
Supplement: Supplementary file 1 [file bdrb0092-0152-SD1.doc]

**Supplement Table 1**

Summary of Animal Studies in Included in Caffeine Update Review

| **Publication** | **Purpose** | **Species and Source**  **Study Guidelines** | **Treatment Route**  **and Period** | **Animals/Grp - Dosage**  **Est. Human Equiv.** | **Results/Comments**  **Strengths-Weaknesses** |
| --- | --- | --- | --- | --- | --- |
| Adén U, Herlenius E, Tang LQ, Fredholm BB.  (2000).  Maternal caffeine intake has minor effects on adenosine receptor ontogeny in the rat brain.  *Pediatr Res*. 48(2):177-83. | Examine influence of chronic pre- and postnatal treatment with a dose of caffeine on development of adenosine A1 and A2A receptors and their corresponding mRNA, as well as on benzodiazepine binding sites presenting in the rat pup brain. | Rats  44 Wistar rats and litters  Source not stated  Study conducted in Sweden  Study guidelines: none reported | Beginning gestation day 2 and continuing throughout gestation and postnatal life  Caffeine administered in drinking water | 21 – ordinary tap water  23 – 0.3 g/L in drinking water  water  Exposure period = GD 2 through gestation and postnatal life Note: consumed dosages vary throughout gestation, lactation and individual consumption by dams and pups; dams (0.250 kg) would consume approximately 3-4 mg/kg; pups (0.025 kg) should consume approximately 300 mg/kg/day after lactation day 14)  **Estimated human equivalent:** - Low doses of caffeine were given in drinking water  -Considered to be about what wo-men might drink during pregnancy (up to 3 cups of coffee/day) | **Comments:**  - Low-dose caffeine-exposure during gestation and postnatal life had minor effects on development of adenosine A_1_ and A_21_ receptors and GABA_A_ receptors in rat brain.  - Minimal, if any changes, observed in the adenosine receptor in fetuses or pups or in development of the rat brain.  - The results here conflict with other studies, but this may be a matter of the dose used in this study versus doses used in other studies.  - Study results may be reassuring to pregnant and breast-feeding mothers who drink coffee in moderation.  - Major finding – Administration of caffeine at doses resembling those consumed by humans does not significantly influence the development of receptors known or believed to be affected by caffeine. These results contrast to other publications indicating that caffeine modified adenosine receptors and/or behavior. |
| Albina ML, Colomina MT, Sanchez DJ, Torrente M, Domingo JL. (2002).  Interactions of caffeine and restraint stress during pregnancy in mice.  *Exp Biol Med* 227 (9): 779-85. | Investigate interactions of caffeine and stress by evaluating maternal and developmental toxicity in mice of combined exposure to caffeine and restraint stress.  Remarks – although restraint alone (not noted by author) and 30 mg/kg/day and higher doses of caffeine combined with stress affected maternal and developmental toxicity, the relevance of the restraint method to human exposure is questionable. | Mice  Swiss Mice  Mature male (for mating only) and female mice  Criffa, Barcelona, Spain  **Study guidelines:** none reported | Gestational days 0-18  Daily  Gavage – vol: 0.20 ml/30 g bw  Vehicle – deionized water  Restraint – 2 hours following administration  Restraint was done by placing each mouse in a tube and taping its paws down for two hours post-dosing each day.  A previous study had shown that one oral dose of caffeine (30 mg/kg) and aspirin (250 mg/kg) administered concurrently with 14 hours of restrain stress on GD 9 slightly increased both maternal and developmental toxicity. | Unrestrained  13 - 0 Control  10 - 30 mg/kg -  14 - 60 mg/kg  10 - 120 mg/kg – significant reductions in bw and bw gain, terminal bw, gravid uterine weight; caffeine induced developmental toxicity significantly increased  Restrained for 2 hours  11 - 0 Control – no adverse effects  10 - 30 mg/kg – enhanced adverse effects noted above  13 - 60 mg/kg – enhanced adverse effects noted above  13 - 120 mg/kg – all died following first treatment (number not identified)  Caffeine doses were relevant to cover NOELs (30 mg/kg) to maternally toxic doses (120 mg/kg).  **Estimated human equivalent:**  30 mg/kg equivalent for 60 kg woman is 12 cups of very strong coffee | **Results:**  **Maternal toxicity:** Present both with and without added stress (i.e. at 30 mg/kg and higher doses). Caffeine exposure reduced maternal weight gain, feed consumption and terminal body weight; in some instances, this stress model significantly increased many of these maternal effects. Differences in maternal parameters observed mainly in groups administered 60 mg/kg (unrestrained) and groups given 30 and 60 mg/kg (restrained). Authors stated that no significant differences occurred between groups exposed to 30 and 60 mg/kg/day. (restrained).  **Developmental** (embryo/fetal) toxicity: Overall effect on fetal body weight at 30 mg/kg and higher (not restrained) and early resorptions at 120 mg/kg (not restrained). Restraint at 30 and 60 mg/kg increased late resorptions and reduced fetal body weight, and at 60 mg/kg, increased early resorptions. At 120 mg/kg/day (restrained) all dams died. No external, internal or skeletal malformations observed; only cleft palate in 60 mg/kg with restraint; some bone retardations noted.  Noteworthy: interaction between restraint stress and caffeine on maternal and developmental toxic effects.  Dams subject to stress only appear to have increased resorption and reduced fetal body weights. Confounding factors that make epidemiological analysis difficult (Christian and Brent, 2001.  - No effect on implantation.  - Restraint alone caused maternal and embryo/fetal toxicity (authors did not recognize restraint alone causing any maternal or developmental toxicity).  - Maternal and Embryo/fetal toxicity were increased with restraint and caffeine down to 30 mg/kg (reductions in maternal body weights and increased postimplantation loss).  - Authors suggest women under stress should reduce caffeine levels to 10 mg/kg (4 cups strong coffee or 8 cups weak coffee).  **Issues**:  **Comments:**  It is well known that stress factors may play a part in the relative effects of caffeine. However, caffeine consumers are subject to multiple confounding factors that make epidemiological analysis difficult (Christian and Brent, 2001).  - Does taping of paws for two hours mimic or create the same stress conditions as pregnant women may experience? Not discussed at all in the study. No mention of any of the physiological consequences of stress from tapping down paws. |
| Asadifar M, Yazdani M, Sadeghpour R, Bruno C, Green J, Nakamoto AT, Hosseini P, Fahami F, Gottschalk S, Nakamoto T. (2005).  Combined effects of caffeine and malnutrition on the newborn rat’s myocardium.  *Food Chem Toxicol* 43(3): 451-6. | Study designed to evaluate combined postnatal effects of caffeine intake and malnutrition during lactation and possible effects on Cu deficiency in the growing neonatal-rat heart.  The evaluation is postnatal and not relevant to the current literature search. | Rats  Sprague Dawley Rats  Time mated females  Harlan Company, Indianapolis, IN  **Study guidelines:** none reported | Untreated until birth  8 pups assigned to each dam  Diet – feed provided ad libitum  Dose was 4 mg/100 g bw – called a heavy dose  Day 10 – Dams: milk, blood collected; heart not examined.  Pups: killed, blood collected, heart removed; plasma milk and heart examined for Cu content. | 10 - 20% protein diet Control  10 - 20% protein diet + 4 mg/100 g bw  10 - 6% protein diet  10 - 6% protein diet + 4 mg/100 g bw  Dose of 4 mg/100 bw – called a heavy dose – just over 4 cups a day (dose for a 50 kg woman consuming 4 cups, each containing 100 mg = 80 mg/kg) | **Comments:**  **-** Treatment was postnatal –Study not relevant  - Caffeine diffuses easily into breast milk  - PCM (protein calorie malnutrition) - public health issue crosses all races; seen in poverty, but also in socially chaotic homes, food diets.  - Examination of % heart weight decrease may show that caffeine with malnutrition might exert less effect on heart weight relative to body weight compared to that of the caffeine–supplemented normally nourished group. Results may be related to absence of histopathology in the malnourished caffeine group compared to malnourished non-caffeine group.  **Results:**  - Maternal body weight and pup weights lower (but not significantly) in all groups compared to Control on Day 1 (birth day). Significant decreases in maternal body weight Days 5 and 10 due to nutrition. - Significant decreases in pup weights Day 10 due to nutrition.  - No significant differences in dams’ plasma and milk caffeine levels in groups 2 and 4 (caffeine-treated).  -Pups’ plasma caffeine levels in group 4 were significantly less than that of group 2.  **Surprising result:** Caffeine exposure affected Cu status more in normally nourished rats than in malnourished rats.  **Weaknesses:**  - 20-30% of adults consume 5-6 cups of coffee/day; up to 85% women consume coffee after birth (up to 19 cups per day). Seems unlikely that 19 cups of any liquid is consumed. |
| Bahi N, Nehlig A, Evrard P, Gressens P. (2001)  Caffeine does not affect excitotoxic brain lesions in newborn mice.  *Eur J* *Paediatr Neurol.* 5(4):161-5. | To examine effects of caffeine on neonatal excitotoxic lesions of the periventricular white matter; study mimics several aspects of human periventricular leukomalacia.  First set of experiments (pups treated IP); designed to mimic caffeine exposure of human pre-term, infants in neonatal intensive care units.  Second set of experiments: to test effects of maternally consumed caffeine on neonatal excitotoxic lesions in the offspring. | Mice  Swiss Mice  Source not identified  Study conducted in France  **Study guidelines:** none reported | IP administration not appropriate for comparison with human exposures  Set I of experiments  IP once daily to pups on PND 0  ½ dosage IP once daily PND 1-3  caffeine diluted in  5-ul volume of phosphate buffer saline (PBS)  Brain damage induced by intracerebral administration of ibotenate on PND 2; 5 ug ibotenate diluted in 2 uL PBS-0.02% acetic acid on day 7.  Set II of experiments  IP once daily to pregnant dams on embryonic days 8 to 18. | Set I of experiments  Pups once daily IP  9 - Control PBS  20 - 10 mg/kg caffeine once daily on PND 0; followed by 5 mg/kg PND1-3.  Set II of experiments  Pregnant dams once daily IP; gestation days 8-18.  3 - Control 100 ul PBS  2.5 mg cafffeine ( 5 mg/kg caffeine citrate) (in 100 ul PBS)  11- control pups injected with ibotenate on PND 2  10- caffeine exposed pups injected with ibotenate on  PND 2  Second group of pregnant dams once daily IP; gestation days 8-11.  3 - Control 100 ul PBS  3 - 12.5 mg/kg caffeine (in 100 ul PBS)  12- control pups injected with ibotenate on PND 2  11- caffeine exposed pups injected with ibotenate on  PND 2  **Estimated human equivalent:**  None reported | **Comments:**  - No deaths; tonic and tonic-clonic seizures (epileptic manifestations) seen in most pups during first 12 hours after intracerebral injection with ibotenate.  - Route was IP – not appropriate. The doses were low with no effects occurring.  - Study showed that in mouse model (neonatal exitotoxic lesion of the periventricular white matter mimicking human periventricular leukomalacia) “caffeine did not exacerbate brain lesions despite potential induction of glutamate release induced by the adeonosine receptor blockade”.  - Study supports lack of toxicity of caffeine in mouse model of periventricular leukomalacia.  - Data suggest neonatal caffeine exposure might not affect clastic lesions in pre-term infants. |
| Björklund O, Kahlström J, Salmi P, Ogren SO, Vahter M, Chen JF, Fredholm BB, Daré E. (2007).  The effects of methylmercury on motor activity are sex- and age-dependent, and modulated by genetic deletion of adenosine receptors and caffeine administration.    *Toxicology*. 241(3):119-33. | To determine if adenosine receptor system affects the developmental neurotoxicity caused by methylmercury (MeHg).  Effect of low dose exposure to MeHg on behavioral outcomes when A1 and A2A adenosine receptors were either partially blocked by caffeine treatment or eliminated by genetic modification. | Mice  3 types:  1) A_1_R KO (adenosine A_1_ receptor knock-out)  2) A_2A_ R KO (adenosine A2_A_ receptor knock-out)  3) WT Control mice  Dept of Physiology and Pharmacology, Karolinska Institute, Sweden  **Study guidelines:** none reported | Gestational day 7 through lactation  Chemicals and caffeine administered to dams in drinking water | Chemicals added to drinking water  Group 1 0.2 mg/L MeHg  Group 2 0.3 g/L caffeine  Group 3 0.2 mg/L MeHg + 0.3 g/L caffeine  Group 4 Control – tap water only  Above 30 mg/kg specified for the NOEL for caffeine, but the authors say this is about four cups a day.  Number of animals per group not specified  **Estimated human equivalent:** none reported | **Comments:**  -Consequences of MeHg toxicity during gestation and lactation can be reduced by adenosine A1 and A2A receptor inactivation, either by genetic deletion or by treatment with caffeine, their antagonist.  - Amounts of mercury in brains analyzed.  - Behavioral tests conducted.  - Data shows exposure to mercury or caffeine during brain development causes changes in dopaminergic functions.  - Exposure to MeHg affected male mice more.  - Behavioral changes from caffeine were independent of sex.  - Important role of A1 and A2A receptors for neurotoxic effects of MeHg and action of caffeine.  - Essentially a study of Methlymercury with caffeine being used as a tool agent to block the effects of methlymercury on adenosine receptor I in the mouse.  - Caffeine did block some of the effects of methlymercury, indicating an effect on the adenosine receptor.  - The authors suggest that the caffeine exposure prenatally may have implications on a tendency for substance abuse later in the life. |
| Bodineau L, Cayetanot F, Sådani-Makki F, Bach V, Gros F, Lebleu A, Collin T, Frugière A. (2003).  Consequences of in utero caffeine exposure on respiratory output in normoxic and hypoxic conditions and related changes of Fos expression: a study on brainstem-spinal cord preparations isolated from newborn rats.  *Pediatr Res*. 53(2):266-73. | To evaluate influence of caffeine exposure in utero on the respiratory control in the newborn rat. | Rats  Pregnant Sprague-Dawley rats  Source – not identified  Study conducted in France  **Study guidelines:** ECC Council Directive (86/609/EEC) – regard animal care and use | 0.02% caffeine provided in drinking water which was changed every 2 days.  Treated water provided throughout gestation.  **Estimated human equivalent:**  70 mg/kg/day ingested by pregnant rats was considered equivalent to 30 mg/kg/day in humans. The authors considered the 49 mg/kg/day of caffeine dose used in study to represent “moderate” intake for humans.  - Author cites case of newborn intoxicated by caffeine; mother drank 24 cups of coffee/day during pregnancy. Newborn experienced apnea episodes attributed to methylxanthine withdrawal. Maternal consumption of this amount of caffeine is questionable | 8 dams Control  8 dams 0.02% caffeine in drinking water  Experiments conducted on brainstem-spinal cord preparation from newborn rats (1-3 days old)  37 pups Control  35 pups caffeine-treated  Dose in the drinking water to the dams was reported as approximately 49 mg/kg | **Comments:**  In vivo exposure followed by an in vitro study of isolated brain stem and spinal cord from newborn rats.  - Analysis of central respiratory drive (estimated by recording C4 ventral root) activity correlated to Fos pontomedullary expression.  - Caffeine-treated group showed higher respiratory frequency than observed in control group.  - Under normoxic conditions changes in respiratory rhythm may be due to decrease in neuronal activity of medullary structures.  - Under hypoxic conditions, typical hypoxic respiratory depression associated with changes in medullary Fos expression was observed.  - Hypoxic respiratory depression increased after in utero exposure to caffeine and coincides with increased Fos expression in area postrema and nucleus raphe obscurus.  - Results support idea that exposure in utero to caffeine could affect central respiratory control.  - There was an increase in the respiratory depression encountered during hypoxic episodes consecutive to apnea in human newborns. However, other factors, such as the influence of the peripheral and suprapontine areas of the nervous system may produce different results in vivo.  **Weakness;** Observations are based on in vitro evaluations of isolated brainstem-spinal cord preparations of newborn rats.  **Strengths:**  - Author notes differences in metabolism of caffeine in rats and humans has been established; half-life much shorter in rats. |
| Boyer M, Rees S, Quinn J, Grattan-Miscio K, McCallum M, Saari MJ. (2003).  Caffeine as a performance-enhancing drug in rats: sex, dose, housing, and task considerations.  *Percept Mot Skills*. 97(1):259-70. | To test the performance enhancing effects of caffeine in a modified forced swim task and dominance task using male and female rats.  - Housing of pups considered important environmental factor | Rats  Offspring of Charles River Wistar female rats  - 5 rats bred at Nipissing University, Ontario, Canada  - 30 male and 30 female pups cross fostered and allocated to each of 4 litters (approx. 15 pups/litter)  - pups housed in enriched or isolated environments  **Study guidelines:** none reported | Administered IP in 0.9% saline to pups  Housing factor  15 male pups housed together  15 female pups housed together  15 male pups housed separately  15 female pups housed separately  IP route is not appropriate for comparison with human exposures | 20 – Control – saline only  20 – 10 mg/kg caffeine  20 – 20 mg/kg caffeine  Three injections administered per rat pup, one each 15 minutes prior to the open-field test, the dominance test and the endurance task; grip test was evaluated within 20 minutes of injection following open-field test  IP doses of 10 and 20 mg/kg are not possible to appropriately extrapolate to human exposure.  **Estimated human equivalent:** Paper deals entirely with rats; no human discussion. | **Comments: This was a postnatal evaluation – not a study of developmental toxicity.**  **-** Rats respond to caffeine as an interactive function of sex, housing, dose and task characteristics.  - Performance enhancing properties of stimulant drugs may be result of complex interplay of variables (including environment).  - Simple generalizations are questionable.  - Results underline importance of housing, sex and task (open field, grip test, dominance task, forced-swim task) in evaluating behavioral elements of caffeine-induced stimulant effects.  - Males housed in isolated environments appeared to prefer stimulants.  - Grip test: 10 mg/kg dose increased hang time.  - Study demonstrated the different outcomes of various behavioral tests and the influence of housing conditions and sex on these studies.  -Paper deals entirely with rats; no human discussion. |
| Burdan F. (2004).  Developmental effects of propyphenazone in analgesic and antipyretic combination with caffeine or paracetamol.  *Hum Exp Toxicol*. 23(5):235-44. | To determine influence of OTC mixture of propyphenazone with caffeine or paracetamol on prenatal development. | Rats  Wistar albino rats from commercial breeder in Warsaw-Rembertow, Poland  Bred at Testing Facility  **Study guidelines**: local guidelines noted but not identified. | Gestation days 8-14  Mixture prepared daily with 3:1 proportion or 3:5 proportion in Tween 80 (Sigma) and diluted with distilled water  Administered by oral gavage | 16 - Control  Propyphenazone:caffeine 3:1 ratio  12 - 2.1:0.7 mg/kg bw  12 - 21:7.0 mg/kg bw  14 - 210:70.0 mg/kg bw  Propyphenazone:  paracetamol 3:5 ratio  13 - 2.1:3.5 mg/kg bw  17 - 21:35.0 mg/kg bw  12 - 210:350 mg/kg bw  **Estimated human equivalent:** none reported. | **Comments:**  - Dose dependent effects on fetal body weight/length and placenta weight in mid and high dose groups.  - No increase in external or internal congenital anomalies found in any of exposed groups.  - No maternal deaths or behavioral changes observed.  - Additional studies needed to be able to evaluate human risk.  - Authors reported maternal and embryo-fetal effects at 7.0 mg/kg dose of caffeine in combination with propyphenazone, but authors note that the “current results also support experimental clinical and epidemiological data regarding prenatal safety of low doses of caffeine extensively discussed by Christian and Brent (1991)”.  - Low number of dams, short period of dosing and species differences indicate more confirmatory studies needed to conclude possible consequences in humans.  - Only animal results reported; no specific references to humans made.  - Human maternal illness (including fever, pain and others) during pregnancy |
| Burdan, F. (2002).  Effects of prenatal exposure to combination of acetaminophen, isopropylantipyrine and caffeine on intrauterine development in rats.  *Hum Exp Toxicol.* 21(1):25-31. | To examine the effects of acetaminophen (paracetamol), isopropylantipyrine (propyphenazone) and caffeine on the fetal development of rats. | Rats  Wistar albino rats from commercial breeder in Warsaw-Rembertow, Poland  Bred at Testing Facility  **Study guidelines**: international and local guidelines cited and referenced in publications but not specifically identified. | Gestation days 8-14  Mixture prepared daily with 5:3:1 proportion or 3:5 proportion in Tween 80 (Sigma) and diluted with distilled water  Administered by oral gavage | 29 - Control  Doses of caffeine wee 0.7 mg/kg, 7.0 mg/kg or 70 mg/kg given in combination with other drugs.  Acetaminophen:isopropy-lantipyrine: caffeine 5:3:1 ratio (A:I:C)  19 - 3.5:2.1:0.7 mg/kg bw A:I:C  16 - 35.0:21.0:7.0 mg/kg bw A:I:C  15 - 350.0:210.0:7.0 mg/kg bw A:I:C  **Estimated human equivalent:**  Mixture used in study commonly marketed OTC in Europe, Central and South America. | **Comments:**  - No maternal deaths or changes in behavior noted.  - No significant differences in number of fetuses, resorptions, preimplantation and postimplantation loss.  -No differences in feed consumption  - Decrease in maternal body weights in high dose group on GD 14, and in mid and high dose groups at termination (GD 21).  - 294 control group skeletons examined: 517 treated group skeletons examined.  - No fetal deaths reported.  - Significant decrease in fetal body weight and length in high dose group.  - Low insignificant increase in skeletal anomalies in all exposed groups.  - 5:3:1 (A:I:C) mixture given during GDs 8-14, not teratogenic to offspring.  - Mixture embryo-toxic only in highest dose group.  - Effects only at the highest dose level – some maternal and developmental toxicity  - Caffeine frequently added to drugs to improve patient’s mood and feeling.  - Author cites differences in animal metabolic pathways and reactions to xenobiotics as factor making it difficult to extrapolate results to humans. |
| Burdan F, Madej B, Wójtowicz Z, Maciejewski R, Radzikowska E.  (2000).  The effects of short-time caffeine administration on skeleton development in Wistar rats.  *Folia Morphol* (Warsz). 59(2):91-5 | To evaluate the influence of caffeine on skeletal ossification.  - Aim of study to find potential teratogenic effect of low-doses of caffeine | Rats  Wistar rats originally obtained from commercial breeders (Rembertow, Poland) and grown in Medical Univ. School, Lubin, Poland.  Rats mated at Testing Facility  **Study guidelines:** none reported. | Gestation days 8-14.  Administered once daily – oral bolus (gavage)  Many other studies administer caffeine in drinking water, not bolus.  Caffeine grounded with Tween 80 and diluted in distilled water. | 10 - Control – Tween 80  19 - Control – Untreated  7 - 0.7 mg/kg bw  9 - 7.0 mg/kg bw  7 - 70.0 mg/kg bw  **Estimated human equivalent**:  None specified. Author notes “limitations” in extrapolating data to humans. | **Comments:**  - Treated females: no change in feed and water consumption and body weight compared to controls.  -520 skeletons from control and treated groups examined  - No external malformations noted.  - Insignificant number of skeletal malformations, external hematomas and internal malformations noted.  - Caffeine administered once daily up to 70.0 mg/kg bw (GDs 8-14) did not cause any teratogenic effect.  - Maternal and fetal caffeine blood levels high for short period of time after bolus treatment.  - Fast metabolism of caffeine protected fetus from constant caffeine exposure as in studies done with caffeine in drinking water exposure.  - Lack of effects noted may be due to method of administration (bolus in this study compared to continuously in drinking water in other studies).  - Authors concluded that caffeine administered once daily during the whole second trimester in doses up to 70 mg/kg did not cause any teratogenic effect.  - Author notes “limitations” in extrapolating data to humans.  - Only animal results discussed.  - Author “suggests” caffeine has “ influence” on humans. |
| Burdan F, Siezieniewska Z, Urbanowicz Z. (2001).  Combined effects of acetaminophen, isopropylantipyrine and caffeine on pregnant and nonpregnant liver.  *Hum Exp Toxicol.* 20(11):569-75. | To evaluate combined effects of acetaminophen (APA) and isopropylanipyrine (IPA) and caffeine on pregnant and non-pregnant liver. | Rats  Outbred albino Wistar rats from Animal Breeding Station, Warsaw, Poland  !52 animals divided into pregnant and non-pregnant groups (15 per group but varies)  **Study guidelines**: none reported. | Gestational days 8 to 14.  Gavage administration  Mixtures prepared in 5:3:1 ratio (acetaminophen, isopropylantipryine and caffeine)  Level of caffeine was so low that no effects observed in this study could be attributed to caffeine. | Combined mixture of APA:IPA:caffeine  Non-pregnant  15 - Control Tween 80 + water  15 - 3.5:2.14:0.7 mg/kg bw  15 - 35:21.4:7.0 mg/kg bw  15 - 350:214:70.0 mg/kg bw  Pregnant  10 - Control Tween 80 + water  16 - 3.5:2.14:0.7 mg/kg bw  10 - 35:21.4:7.0 mg/kg bw  14 - 350:214:70.0 mg/kg bw  **Estimated human equivalent:**  None given. Author notes “limitations” in extrapolating data to human intake. | **Comments:**  - No maternal deaths or changes in behavior observed.  - Liver toxicity reported in previous studies using doses greater than in this study and for longer periods of time.  - Administration of APA:IPA:caffeine to non-pregnant rats only slightly impaired liver function at highest dose.  - During second week of administration, administration of mixture resulted in hepatotoxic effects in pregnant rats, increasing with higher dose.  - Pregnant rat’s liver more sensitive to tested xenobiotics than non-pregnant rat’s liver.  - The pregnant maternal liver was more susceptible to injury than the non-pregnant liver.  - Caffeine used in combination with other drugs for “fever and pain”. |
| Burdan F. (2003)  Intrauterine growth retardation and lack of teratogenic effects of prenatal exposure to the combination of paracetamol and caffeine in Wistar rats.  *Reprod Toxicol* 17 (1): 51-8. | To determine the effect of paracetamol and caffeine administered together. | Albino rats  Wistar - sexually mature males and females  Warszawa, Poland  **Study guidelines:** FDA GLP CFR Mar 21, 1994:56FR1200; also international and local guidelines cited. | Paracetamol:caffeine prepared 5:1  Gestation days 8-14 (sperm = day 1)  Only combination groups – oral dosing – from 0.7 mg/kg of caffeine to 70 mg/kg of caffeine | 16 - 0 Control  15 - 3.5 mg/kg paracetamol and 0.7 mg/kg caffeine  15 - 35 mg/kg paracetamol and 7 mg/kg caffeine  15 - 350 mg/kg paracetamol and 70 mg/kg caffeine  **Estimated human equivalent**: low dose equivalent to drugs available OTC in Europe and other countries. | - Paracetamol is an analgesic marketed OTC in Europe and elsewhere and generally considered safe during gestation; it is commonly mixed with stimulants including caffeine.  **Comments:**  - Maternal effects on body weight at all doses.  - Fetal weight effects only at highest dose.  - Supports all previous work that at 70 mg/kg it is not unexpected to see decreased fetal weights in the presence of maternal toxicity.  - No fetal malformations.  **-** Effects of caffeine given in combination with other chemicals is not entirely understood.  - Treatment with paracetamol alone is preferable to treatment with caffeine  - Second generation studies recommended. |
| Buttar HS, Jones KL. (2003)  What Do We Know about the Reproductive and Developmental Risks of Herbal and Alternate Remedies?  Birth Defects Research Part B -  68 (6), pp. 492-493. | To consider the reproductive and developmental risks of herbal remedies and alternative therapies. | NOT APPLICABLE | NOT APPLICABLE | NOT APPLICABLE | **Symposium (not a study)**  **Comments:**  - Supports the need for testing but gives no information on caffeine.  - Description of Symposium regarding issues surrounding herbal and alternate remedies  - Notes false sense of security promoted by media regarding “natural” remedies  - Possible toxicities and herbal-drug interactions noted  - Symposium sponsored by and presented by Teratology Society.  - Danger to public for considering herbal and alternate remedies safe because they are “natural”.  - Herb-drug interactions can occur if patients take more than one drug at a time.  - System examination of efficacy, safety and quality missing regarding “traditional” herbals. |
| Chorostowska-Wynimko J, Skopińska-Rózewska E, Sommer E, Rogala E, Skopiński P, Wojtasik E. (2004).  Multiple effects of theobromine on fetus development and postnatal status of the immune system.  *Int J Tissue React*. 26(1/2):53-60. | To investigate the effects of theobromine (active derivative) of caffeine on fetal development and postnatal status of the immune system. | Mice  Inbred female Balb/c mice (2 months old).  Source not identified.  Study conducted in Warsaw, Poland  Pregnancy terminated day 18 for some mice.  Some mice allowed to deliver; offspring raised to 4 or 6 weeks.  **Study guidelines**: local guidelines noted regarding animal care and use | Mice “fed” theobromine (Sigma Aldrich), a metabolite of caffeine, and normal laboratory chow during pregnancy and lactation.  Route of administration not specifically identified; diet assumed. | Control – N = 8  2 mg/day theobromine – Not cited  6 mg/day theobromine – N = 8  Number of mice not specified for 2 mg/kg group.  **Estimated human equivalent**:  None reported. Humans get caffeine through coffee, tea, soft drinks, cocoa and chocolate. | **Comments:**  **Cites “moderate intake – up to 400 mg/day of caffeine) is not associated with noteworthy adverse effects**  - Study showed no effect on number of corpora lutea on size of litters of dams.  - Theobromine feeding resulted in decreased embryo growth assessed by weight and decreased angiogenic activity in tissue. However, the number of embryos per litter was only 10 (8 litters per group), and the difference in mean embryo weight was 0.87 and 0.65 g, values that were identified as significant but based on only a Student’s t-test. The same applies to the angiogenic activity of the embryonic tissue homogenate (control = 18.5; theobromine = 16.7).  Postnatally, the 4-werk old progeny had shorter limbs, forefeet and hind legs and an increased spleen weight. The authors suggested that theobromine may affect the embryo[‘s growth process, which would not be unexpected, in consideration that such has been shown many times previously for caffeine. High breast milk/serum concentrations were achieved (0.829±0.038) [providing theobrommine delivery to the suckling neonates.  - Possible abnormalities in immune system status in suckling mice noted in both cellular and humoral response  - Study advised diverse balanced diet and restraint on coffee and soft drinks rich in caffeine.  - High dose caused decreased fetal weight and offspring had smaller bones – questionable if any effects on any immune end points.  - No potential adverse effects on human health observed in moderate intake of up to 400 mg/day caffeine. |
| Clyman RI, Roman C. (2007).  The effects of caffeine on the preterm sheep ductus arteriosus.  *Pediatr Res*.  62(2):167-9. | To determine effects of caffeine on preterm sheep ductus arteriosus.  - Recent study showed infants randomly assigned to caffeine treatment had less need for pharmaco-logic and/or surgical closure of patent ductus arteriosus (PDA); an unexpected finding. | Sheep  Source not identified.  Study conducted at Cardiovascular Research Institute and Department of Pediatrics, Un. Calfornia, San Francisco, CA  **Study Guidelines**: none reported. | In vitro study – looking at fetal lamb ductus ateriosus | Examined 24 lamb fetuses at 105±4 days (147 days = term) to determine direct effects of caffeine on isometric tension of ductus arteriosus.  dose was 0.003 to 0.3 mM  **Estimated human equivalent**: none reported | **Comments:**  - In vitro study of isolated rings of the fetal lamb ductus arteriosus found no evidence of a direct effect of therapeutic caffeine concentrations on ductus contractility or the ductus’ contractile response to grade increases in oxygen , inhibition of endogenous prostaglandin and nitric oxide production. The differences between their in vitro findings and the reported in vivo findings were hypothesized as potentially related to exogenous adenosine, in vivo, the ductus is exposed to shear stresses that were not considered in the in vitro model; caffeine might interact with shear-related signaling or at some site in the ductus, altering the production of circulating substances that might affect ductus contractility. |
| Colomina, MT, Albina ML, Sanchez DJ, Domingo JL. (2001)  Interactions in developmental toxicology: Combined action of restraint stress, caffeine, and aspirin in pregnant mice.  *Teratology* 63:144-151. | **Purpose:** To assess the effect of maternal stress on mice when exposed concurrently to caffeine and aspirin. | Swiss Mice  Mature male and female mice  Interfauna, Ibérica, Barcelona, Spain  **Study guidelines:** none reported. | Single dose on gestational day 9 (vaginal plug = day 0 of gestation)  Caffeine and aspirin dissolved and administered in deionized water (volume not specified)  Gavage  Restraint – 14 hours  Animals in a tube with paws taped down. | Unrestrained  13 - 0 Control  11 - 30 mg/kg caffeine + 0 ASA  12 - 0 mg/kg caffeine + 250 mg/kg ASA  7 - 30 mg/kg caffeine + 250 mg/kg ASA  Restrained for 14 hours  11 - 0 Control  10 - 30 caffeine mg/kg + 0 ASA  8 - 0 Caffeine + 250 mg/kg ASA  13 - 30 mg/kg caffeine + 250 mg/kg ASA  **Estimated human equivalent:** not reported.  Reference that  Caffeine consumed by humans in coffee, tea, soft drinks and cocoa products. Heavy consumption of caffeine (>300 mg/day) associated with premature birth and low birth-weight infants. | **Comments:**  - Interactions of restraint and caffeine do indicate slightly more maternal (greater body weight loss) and fetal toxicity (increased resorption but this appears to be from one mouse since the SD was 30.5 verses a range of 4.84 to 17.0 for the other groups) and delay in ossification of skull, sternum and vertebral bones.  - Single dose of caffeine or aspirin to pregnant mice at doses of 30 and 250 mg/kg did not cause significant maternal or developmental toxicity.  **Issues:**  - Does taping of paws for 14 hours create the same stress as a pregnancy women may go through? Not discussed at all in the study.  - No mention of the any of the physiological consequences of stress from tapping down paws.  - Said this was appropriate stress as demonstrated in previous work – which appears to be same type of **study on restraint but with methylmercury.**  **- Clinical relevance:** Model-oral administration of aspirin with caffeine to mice on GD 9 is not analogous to pregnant women under stress who drink coffee and aspirin.  - More studies needed to evaluate combination of aspirin and caffeine and impact of restraint (stress). |
| da Silva RS, Richetti SK, da Silveira VG, Battastini AM, Bogo MR, Lara DR, Bonan CD. (2008).  Maternal caffeine intake affects acetylcholinesterase in hippocampus of neonate rats.  *Int J Dev Neurosci.* 26(3-4):339-343. | To evaluate rat maternal caffeine intake (1 g/l) on acetylcholine degradation and acetylcholinesterase expression from hippocampus of 7-, 14-, 21-day old neonates in caffeine-treated and control groups. | Rats  Pregnant Wistar rats  Source and strain not reported.  Study conducted in Brazil  **Study guidelines**:  Procedures in accordance with regulations of National Institutes of Health Guide for Care and Use of Laboratory Animals. And Institutional Ethics Committee (CEP06/02980) of the Pontifica Universidade Católica do rio Grande do Sul, Brazil) | 1 g/L caffeine in drinking water  Although not defined, appears to include mating, gestation and portions of lactation, as noted below.  During lactation  Control - none  Group 1 – up to 7 days  Group 2 – up to 21 days  MK-801 (0.25 mg/kg, IP)  Evaluations were made of total homogenates of the hippocampus and included enzyme assays for AChE activity and a semi-quantitative RT-PCT analysis of gene expression for acetylchlinesterase expression | 1 g/L caffeine in drinking water  Consumed mL/kg doses were high – 166.21 for caffeine; 168.09 for controls). It is unclear how these amounts were calculated.  Doses of caffeine reported were very high – over 160 mL/kg/day during entire gestation period, although the authors considered them non-toxic.  Dose can not be calculated.  During lactation  Control (n=not reported) – none regular tap water  Group 1 (n=6) – up to 7 days (washout group)  Group 2 (n=9) – up to 21 days  **Estimated human equivalent:**  None reported. | **Comments:**  In rats, acetylcholine reaches mature levels around 8 weeks after birth and the rate of acetylcholine degradation, i.e., as the result of acetylcholinesterase, increase in a time-dependent manner after birth, attaining stability at 21 days of neonatal life.  Concluded caffeine maternal treatment increased hippocampal AChE activity in 21-day old pups without affecting mRNA expressions, which suggests caffeine exerts direct effects on AChE.  - Animal data only analyzed; no comparisons to human intake/effects.  Relevance to humans - time period of treatment considered to correspond with entire gestational time plus approximately the first three years of life.  Study relevant to postnatal developmental considerations, but not malformation, growth retardation or embryo-fetal death.  Authors claim the time period of treatment tested corresponds to the entire gestation time plus the first three years of human life. |
| da Silva RS, Hoffman A, de Souza DO, Lara DR, Bonan CD. (2005).  Maternal caffeine intake impairs MK-801-induced hyperlocomotion in young rats.  *Eur J Pharmacol.* 509(2-3):155-9. | To determine the effects of maternal caffeine intake (1 g/L) on MK-801-induced hyperlocomotion in rat pups. | Pregnant Wistar Rats  Source not identified  Study conducted in Brazil  **Study guidelines**: Procedures in accordance with regulations of Colégio Brasileiro de Experimentação Animal, based on NRC guide for care of animals | 1.0 g/L caffeine in drinking water  Caffeine intake = 206.51 ± 23.14 mg/kg/day =; washout group had 223.90 ± 63.67 mg/kg/day. Tap water consumption for controls was 209.45 ± 26.83 mL/day/kg. Not possible to accurately identify exposure.  Number not specified, but three groups. Tap water group (vehicle) and caffeine solution (1 g/L) provided fro gestational day 1 (presumed that mating detection = gestation day 1) for entire gestation and lactation period. After birth, one caffeine-treated group was given caffeine up to 21 days of age, and a washout group (N = 6) was given caffeine up to 7 days. The animals were tested for locomotor activity at 21 days of age (8 rats per group) | Maternal doses were quite high (206.51 mg/kg/day – although this would vary over the period of administration)  **Estimated human equivalent:**  None reported. | **Comments:**  MK-801 promoted hyperlocomotion in control rats; effect was significantly reduced in caffeine-treated and washout groups.  - Permanent effect after caffeine withdrawal suggests changes during neurodevelopment, mainly on adenosine receptors or neurotransmitter systems modulated by adenosine, such as the glutamatergic system.  - Results reinforce influence of adenosine during mammalian neurodevelopment, with implications for the adult behavioral in response to NMDA receptor antagonists, particularly in locomotor activity.  - Study suggests caffeine during gestation and lactation impairs hyperlocomotor response to NMDA receptor antagonist, MK-801, without affecting normal locomotion. Suggests permanent and adaptive changes occur in brain exposed to caffeine during neurodevelopment.  - Maternal caffeine intake can induce changes in immature central nervous system that persist even after caffeine withdrawal.  -Study is relevant to functional development of the brain, but not to the effects of in utero exposure on morphology, body weight or viability.  -No information reported on maternal animals regarding effects of caffeine, although the dose reported, 206.51 mg/kg/day, as consumed during gestation and lactation would be expected to be toxic. |
| Desfrere L, Olivier P, Schwendimann L, Verney C, Gressens P. (2007).  Transient inhibition of astrocytogenesis in developing mouse brain following postnatal caffeine exposure.  *Pediatr Res.*  62(5):604-9. | To evaluate the full impact of caffeine on the developing brain post natal | Mice  Strain and source not reported  Study conducted in France  **Study guidelines:**  Procedures in accordance with regulations of National Institutes of Health Guide for Care and Use of Laboratory Animals. | Pups  Study uses IP injections into pups at high doses including loading doses. (unclear whether all doses are IP, but such is presumed).  Reported methodology is unclear. Appears to include a loading dose of 10 mg/kg caffeine followed by subsequent doses of 2.5 mg/kg once daily from P4 to P10  PNDs 3-10 - loading dose – 5u/L IP or 10 mg/kg caffeine followed by 2.5 mg/kg once daily PND 4-10.  Pups killed PND 7, 10, 15, 20 (10/group) and PND 40 (8/group)  Dose-response study, one of the 4 loading doses administered on PND 3 (5, 10, 20 40 mg/kg) followed by subsequent doses of 25% of the loading dose from P4 to P10 (10 mice/group) | PND 3-10 loading dose – 5u/L IP or 10 mg/kg caffeine followed by 2.5 mg/kg once daily PND 4-10.  **Estimated human equivalent:**  - Methylxanthines (especially caffeine) used in premature infants to treat episodes of apnea.  - Infant plasma exposure 5-15 mg/L for up to 8 wks.  - Model used: doses to mouse pups PND 3-10 mimics developmental stage of human brain GD 24-38. | **Comments:**  **-** Previous animal studies suggest caffeine exposure make have neurotoxic side effects.  - Following caffeine treatment, some disturbances of astrogliogenesis are visualized by immunohistochemistry in developing mouse brain probably mediated via A2aR.  - Results underline need for assessment of long-term neurodevelopmental consequences of caffeine in human neonates to clarify the benefit/risk of this treatment.  - No effects actually produced.  **Author’s discussion:**  - Possibility of cognitive impairment, attention deficit and behavioral problems in preterm infants treated with caffeine described.  - Short term benefits of caffeine weighed against long term effects.  - More clinical studies needed to evaluate benefit/risk of caffeine treatment in preterm infants.  Study at best shows that following caffeine treatment of neonatal mice, some disturbances of astrogliogenesis are evident on the basis of immunohistochemistry in the developing mouse brain and that the effect is probably mediated by A2aR.  Investigators report that they examined influence of low doses of caffeine on developing mouse brain at ages corresponding to neurodevelopmental stage of human infants who are given caffeine in neonatal intensive care units. Questionable whether this comparison is appropriate.  All tests and treatment postnatal – not relevant to non-clinical in utero exposure, although comparable period of brain development may have been evaluated as those used in clinic.  Study conducted in postnatal period; not relevant to in utero exposure. No effects were produced. Assumed exposure of preterm newborns and P3-P10 mouse pups probably mimic developmental stage of human brain between 24 to 38 weeks of gestation |
| Evereklioglu C, Sari I, Alasehirli B, Güldü, E, Cengiz B, Balat O, Bagci C. (2003)  High dose of caffeine administered to pregnant rats causes histopathological changes in the cornea of newborn pups.  *Med Sci Monit* 9 (5): BR168-73. | Study investigated histopathological effects of caffeine on neonatal rat cornea.  High doses of caffeine administered by IP route; not relevant to human exposure. | Wistar-Albino rats  Mated at the Test Facility  Source not identified  Study conducted in Turkey  **Study guidelines**: conformed to Principles and Guidelines for the Use of Animals in Research, New York Academy of Science’s Committee on Animal Research. | Gestation days 9-21  Caffeine was administered IP – not a relevant route – and PO – relevant and toxic to the pups  .  Groups 2-4 exposed to 25, 50 and 100 mg/kg caffeine daily by IP.  Group 5 exposed daily 50 mg/kg in distilled water by gavage | 10 - 0 mg/kg/day (IP)  10 - 25 mg/kg/day (IP)  10 - 50 mg/kg/day (IP)  10 - 100 mg/kg/day (IP)  10 50 mg/kg/day (gavage)  **Estimated human equivalent:** none reported. | **Comments:**  - High dose of caffeine administered to pregnant rats causes histopathological changes in the cornea of newborn pups.  - No maternal toxicity reported, however, 7 pups miscarried by 2 dams in 100 mg/kg/day caffeine Group 4 (high dose). Because miscarriage is highly unlikely in rats, this was probably a sequelae of IP injection or an incorrectly identified gestation day 1 (the method used was not identified .  .  - No resorbed or stillborn fetuses.  - Group 4 fetuses smaller than other groups.  - Impaired corneal maturation with striking histopathological changes observed along with dose dependent incidence in Group 4 (100 mg/kg/day)  **Human relevance:**  - Author suggests restraint in extrapolating data to humans.  -Women should follow FDA limit of 300 mg/day (2-3 cups of coffee).  -Advises nursing mothers to follow same limit.  - Human neonates have low levels of enzymes needed to metabolize caffeine.  - Elimination of caffeine from blood is slowed during pregnancy.  -Author notes link between caffeine consumption and teratogenesis, fetal resorption and low fetal birth weight in animal studies. |
| Evereklioglu C, Güldür E, Alasehirli B, Cengiz B, Sari I, Pirbudak L.(2004)Excessive maternal caffeine exposure during pregnancy is cataractogenic for neonatal crystalline lenses in rats: a biomicroscopic and histopathologic study. *Acta Ophthalmol Scand* 82(5):552-56. | The ultimate objective was to establish a model for the study of cataract development in rats. This specific study was designed to investigate histologically the influence of maternal caffeine exposure during pregnancy in vivo on development of the crystalline lenses in neonatal rats.  Considered to be a possible model for studying cataract formation in rats, if the appropriate dose of caffeine can be identified. | Wistar-albino rats  Medical Sciences Experimental Research Unit of Erciyes University, Kayseri, Turkey  **Study guidelines:** Ethics committee of Gaziantep University and performed in conformance with the Principles and Guidelines for Use of Animals in Research, Testing, and Education issued by the Committee on Educational Programmes in Laboratory Animal Science, 1991, National Research Council. | Gestation days 9-20  Caffeine was administered IP – not a relevant route  Groups 1-3 exposed to caffeine IP  Group 4 exposed by gavage  Group 5 Control given saline solution IP  Gestation day 1 not defined, but cohabitation of two females per male for two days, with pregnancy defined by sperm in a vaginal smear.  Dams delivered normally (generally on gestation days 20-21, which indicates that sperm probably = gestation day 1). Half of the newborn rats per litter were decapitated at postnatal day 1 and the eyes examined. The remaining litters were raised with their biological mothers and sacrificed and decapitated at postnatal day 30 for eye evaluation.  It is somewhat unclear, but it appears that only one randomly selected eye (right eye) was evaluated., so it appears that each litter and dose group is represented by only one pup at each time interval. | 10 - 25 mg/kg/day caffeine  10 - 50 mg/kg/day caffeine  10 - 100 mg/kg/day caffeine  10 - 50 mg/kg/day caffeine in distilled water (gavage)  10 - Control – saline solution  **Estimated human equivalent**: none reported. Possible human risk noted by author in discussion section of paper. | **Comments:**  - No maternal toxicity observed in any dose group, but two dams in the 100 mg/kg IP group “miscarried a total of 7 pups” – without better reporting of data, these are probably mistimed pregnancies or early deliveries associated with the route of administration..  - Low birth weight and crown-rump length decreased in dose dependent manner.  - Excessive maternal caffeine exposure during pregnancy is cataractogenic for neonatal crystalline lenses in rats: a histpathologic study.  - Increased histopathologic lens changes in high dose (100 mg/kg/day caffeine) observed.  - Author cites FDA 1980 warning for use of caffeine may be associated with increased risk of fetal loss.  - Advice may hold true for nursing mothers too.  - More study needed. |
| Gaytan SP, Saadani-Makki F, Bodineau L, Frugière A, Larnicol N, Pásaro R. (2006).  Effect of postnatal exposure to caffeine on the pattern of adenosine A_1_ receptor distribution in respiration-related nuclei of the rat brainstem.  *Auton Neurosci.*  126-127:339-46. | To examine the ontogeny of the adenosine A1 receptor system in the brainstem of newborn rats after postnatal treatment with caffeine, to mimic the therapeutic administration of caffeine to premature human infants. | Rats  Pregnant Sprague Dawley rats  Source unknown.  Study conducted in Spain at the University of Seville.  **Study guidelines:** European Communities Council Directive (86/609/EEC) regarding animal care and use. | Dams  Pups  Gavage PND 2-6 (birth day = PND 0) | Dams  4 - Control  5 - caffeine-treated  Note: no indication of how or if dams were treated; just divided into 2 groups. Presumably all treatment was postnatal  Pups – Birth = postnatal day 0. Two administrations of caffeine appear to have been given. Pups were given caffeine plus glucose or water plus glucose on days 2-6 postnatal at a volume of 0.05ml/10gBW. Then to maintain serum levels of caffeine at 5-15 mg/L (approximately 0.26-0.77 mM range) over 24 hours of each day, they were given caffeine at 20 mg/kg on day 2 and 15 mg/kg on days 3 to 6.  **Estimated human equivalent:** none reported. | **Comments:**  - Main difference between treated and non-treated rats (PND 6) was in number of immunopositive neurons in two brainstem areas associated with respiratory control.  - Study results indicate interaction of caffeine and respiratory drive during postnatal period.  - Authors suggest using caffeine with care in treating human neonates because it appears to alter the development of the adenosine receptor by causing them to develop earlier in the brain. This may be the mechanism by which caffeine helps in preterm babies that have respiratory problems.  **Human relevance:**  - Caffeine is used to treat human preterm neonates for apnea.  - Positive effect could be related to up-regulation of adenosine A1 receptor expression.  - Long term effects need to be evaluated, since many diseases and illnesses have roots in early fetal life.  - Study shows caffeine affects rat brain development and should be used cautiously in human infants. |
| Gilbert-Barness E. (2000).  Maternal caffeine and its effect on the fetus.  *Am J Med Genet.* 93(3):253. | Review article – not relevant | NOT APPLICABLE | NOT APPLICABLE | NOT APPLICABLE | **Letter to editor:**  - Notes effects in animals at doses of about seven cups of coffee a day.  - Cites others works – all of which have been reviewed.  - Disputes recent publication (Klebanoff, et al., 1999) that equivalent of 6 cups of coffee are associated with spontaneous abortion.  - Risk of low birth weight and spontaneous abortion and neurobehavioral changes at lower consumptions reported by Eskenazi and Nehling.  - Urges the FDA to reassert their advice to pregnant women from 1981 to avoid caffeine or consume it sparingly . |
| Hongu N, Sachan DS. (2000).  Caffeine, carnitine and choline supplementation of rats decreases body fat and serum leptin concentration as does exercise.  *J Nutr.* 130(2):152-7. | **Purpose:** To determine effect of combined caffeine, carnitine and choline with or without exercise on changes in body weight, fat pad mass, serum leptin concentration and metabolic indices in 7-wk old male rats treated for 28 days.  Not relevant to this review. | Rats  Sprague Dawley  7-wk old male rats  Harlan Sprague-Dawley, Indianapolis, IN  **Study guidelines:** none reported. | Caffeine, carnitine and choline combined with diet; provided for 4 weeks  Male rats were fed a diet including caffeine – 100 mg/kg of diet  Rats weighed about 300 g and consumed about 20 g of feed per day. – 6.6 mg/kg bw of caffeine – very low dose | Free access to nonpurified diet (22% protein, 5% fat, 3.83% fiber); endogenous concentrations of choline and carnitine in commercial diet 2.1 g and 30 mg/kg diet.  Diet fortified with caffeine, carnitine and choline (ccc)  10 - Control  10 - supplemented diet  0.1 g/kg ccc  5 g/kg ccc  11.5 g/k ccc  ½ of each group was exercised  ½ of each group was not  **Estimated human equivalent:** Authors note that results may or may not be applicable to humans. | **Comments:**  - No change in feed intake, but body weight was significantly reduced by exercise in both dietary groups.  - Fat pad weights, total lipids of epididymal, inguinal and perirenal regions were significantly reduced by supplements as well as by exercise.  - Authors conclude that indices of body fat loss due to dietary supplements were similar to those due to mild exercise; no interactive effects of the two variables.  - Authors reported no adverse effects.  - Further research needed. |
| Keller BB, Liu LJ, Tinney JP, Tobita K. (2007).  Cardiovascular developmental insights from embryos.  *Ann N Y Acad Sci*. 1101:377-88. | - Overview of insights from investigating cardiovascular (CV) developmental mechanisms in embryos with respect to molecular genetic, environmental and biomechanical explanations for congenital CV malformations (in humans). | NOT APPLICABLE | NOT APPLICABLE | NOT APPLICABLE | **Overview (information provided; manuscript was not a study)**  **Comments:**  - Approximately 30,000 children born annually with congenital heart anomalies.  - Some advances in medicine noted..  - Notes maternal exposure to bioactive chemicals (such as caffeine) can impact embryo/fetal cardiovascular function, growth and outcome.  - No specific discussion of caffeine. |
| León D, Albasanz JL, Ruíz MA, Fernández M, Martín M. (2002).  Adenosine A_1_ receptor down-regulation in mothers and fetal brain after caffeine and theophylline treatments to pregnant rats.  *J Neurochem.* 82(3):625-34. | To determine the effect of caffeine or theophylline on mothers and full-term fetuses when treated with caffeine or theophylline during entire gestation period | Rats  Pregnant Wistar rats  Source not identified.  Study conducted in Spain  **Study guidelines:** none reported. | Treated from gestation day 2 through entire gestation period.  Provided in drinking water (1 g/L) | Control – drug-free tap water  Dose of caffeine 1g/L in drinking water  83.2 mg/kg/day caffeine consumed daily in drinking water  83.8 mg/kg/day theophylline consumed daily in drinking water  **Note: very high doses of caffeine.**  Number of rats per group not specified  **Estimated human equivalent:**  - Assumes 80-180 mg caffeine in a cup of coffee.  - Dose equivalent to one cup of coffee.  - Due to different rates of metabolism 10 mg/kg in rat represents about 250 mg of caffeine in human (about 70 kg) corresponding to 2-3 cups of coffee.  - Caffeine consumed by rats in this study corresponds to 28 mg/kg/day in humans. | **Comments:**  - Chronic caffeine or theophylline intake caused a down-regulation of adenosine A1 receptors in both maternal and fetal brain.  - This could reflect increase in stimulatory activities exhibited by caffeine and theophylline.  - Could increase vulnerability of brain and other tissues to harmful effects of both substances on fetuses.  - Decreased adenosine receptors in maternal and fetal brain. – decreased at these increased doses of caffeine.  - Maternal consumption of caffeine during pregnancy is associated with potentially harmful effects of caffeine and theophylline on developing fetal brain. |
| León, D, Albasanz, JL, Ruíz, MA, Iglesias I, Martin M. (2005a).  Chronic caffeine or theophylline intake during pregnancy inhibits A1 receptor function in the rat brain.  *NeuroScience*  131:481-489. | To study whether caffeine or theophylline chronically consumed during pregnancy affect inhibitory adenylyl cyclase pathway mediated by adenosine, in rat brain of both mothers and full-term fetuses. | Wistar pregnant rats  Source not identified  Study conducted in Spain  **Study guidelines:**  EEC Directive 24 Nov 1986 (86/609) regarding animal care and use and Ethics Committee of the Castilla-La Mancha University. | Gestational day 2 through end of gestation  Dose was in water  1 g/L of caffeine or theophylline.  Rats permitted to deliver naturally.  Maternal and fetal brains removed, frozen and stored for experiments | Control- drug free tap water  Dose of caffeine or theophylline 1 g/L in drinking water  -Average daily caffeine consumption: 83.2 mg/kg.  -Average daily theophylline consumption: 83.8 mg/kg | **Comments:**  -There were no significant differences detected in fetal brain between control and treated animals.  -Caffeine or theophylline chronic intake during pregnancy differently modulates inhibitory adenylyl cyclase pathway causing a loss of system responsiveness only in maternal brain.  -Caffeine and theophylline modulates AC inhibitory pathway mediated by adenosine in both mothers’ and fetuses’ brains.  -Harmful effects of methylxanthines would be specially important and apparent when fetuses reach a complete developed status.  -Attention should be paid to high caffeine or theophylline consumption during pregnancy. |
| León, D, Albasanz, JL, Ruíz, MA, Iglesias I, Martin M. (2005b).  Effect of chronic gestational treatment with caffeine or theophylline  on Group I metabotropic glutamate receptors in maternal and fetal brain.  *J Neurochem* 94(2): 440-51. | To study effect of treatment on pregnant rats during gestation with caffeine or theophylline. Effects on metabotropic glutamate receptor (mgluRs) signal transduction pathway was studied in maternal and fetal brain. | Wistar pregnant rats  Source not identified  Study conducted in Spain  **Study guidelines:** Declaration of Helsinki | Gestational day 2 through end of gestation  Dose was in water – 83.2 mg/kg of caffeine  Rats killed GD 23; fetuses delivered surgically.  Maternal and fetal brains removed, frozen and stored for experiments | 7 - Control – drug free tap water  7 - 1 g/caffeine in drinking water  7 - 1 g/L theophylline in drinking water  Dose was in water – 83.2 mg/kg of caffeine.  **Estimated human equivalent:**  None reported. | **Comments:**  - Effects may occur in brain receptors, actually only in maternal brain, but at this dose it is not surprising.  - Results confirm cross-talk between transduction pathways mediated by adenosine and glutamate.  - Suggests consumption of caffeine and theophylline during gestation should be restricted. |
| López FJ, Alvariño JM. (2000).  Effects of added caffeine on results following artificial insemination with fresh and refrigerated rabbit semen.  *Anim Reprod Sci*.  58(1-2):147-54. | To study effect of addition of caffeine on rabbit semen (stored for up to 96 hours) when 1335 lactating rabbits were artificially inseminated. | Rabbit semen  California x New Zealand (NZW) rabbits  El Senorio de Molina Farm, Spain  **Study guidelines:** None reported. | In vitro exposure of rabbit semen – not directly relevant. | 1335 lactating rabbits artificially inseminated  **Estimated human equivalent:**  not applicable. | **Comments:**  - Concentration of 0.2 mM/L increased spermatozoa motility, higher concentrations adversely affected reproductive parameters.  - Overall, caffeine did not enhance fertility or prolificacy regardless of ability to increase sperm motility.  **Human Relevance:**  - Study was conducted to evaluate rabbit semen treated with caffeine for artificial insemination purposes.  - No human relevance reported**.** However, it may indicate that addition of caffeine to human semen would increase sperm motility. No data are available as to whether it would affect fertility. |
| Lutz J, Beck SL. (2000).  Caffeine decreases the occurrence of cadmium-induced forelimb ectrodactyly in C57BL/6J mice.  *Teratology*. 62(5):325-31. | To study the interaction between caffeine and Cd sulfate at a non-teratogenic dose. | Mice  C57BL/6JBK mice from Jackson Laboratory, maintained at DePaul University, Chicago, IL (for over 15 generations)  **Study guidelines:** none reported | Cadmium sulfate administered IP  Caffeine administered subcutaneously  Vol = 10 mL/kg  On gestation day 9 | 8 - Control 50mg/kg caffeine + saline  9 - 1 mg/kg Cd  10 - 2.50 mg/kg Cd  10 - 5.00 mg/kg Cd  Subcutaneous injection of 0 or 50 mg/kg of caffeine followed Cd injection on gestation day 9  50 mg/kg doses of caffeine, which had no effect, were used to change the frequency of cadmium- induced ectrodactyly.  10 - 1 mg/kg Cd + 50 mg/kg caffeine  10 - 2.50 mg/kg Cd + 50 mg/kg caffeine  10 - 5.00 mg/kg Cd + 50 mg/kg caffeine  **Estimated human equivalent**: none reported | **Comments:**  - Cadmium is well known animal teratogen.  - Studies of interactions between teratogens and non-teratogens (like caffeine) being conducted to perhaps understand mechanisms of teratogenicity and possibly prevent teratogenic effects.  - Statistically significant reduction in Cd-induced abnormalities (eye, abdominal, other skeletal effects) not observed with the addition of caffeine; although there was a downward trend in caffeine supplemented groups.  - Litter size, fetal weight, fetal mortality and dam weight not affected by co-treatment with caffeine.  - Evidence that subteratogenic dose of caffeine can ameliorate Cd-induced forelimb ectrodactyly in this Cd-sensitive mouse strain. |
| Momoi N, Tinney JP, Liu LJ, Elshershari H, Hoffmann PJ, Ralphe JC, Keller BB, Tobita K. (2008).  Modest maternal caffeine exposure affects developing embryonic cardiovascular function and growth.  *Am J Physiol Heart Circ Physiol.*  294(5):H2248-56. | To test the hypothesis that modest maternal caffeine exposure affects in utero developing embryonic cardiovascular (CV) function.  - High resolution echocardiography used to assess maternal and embryonic CV function. | Mice  CD-1 male (for mating 2:1) and female rats  Source not identified.  Study conducted at University of Pittsburgh (PA)  **Study guidelines**: none reported | Treated during embryonic days (9.5 to 18.5) of the 21-day gestational period (note – mice have an 18.5-day gestation period; the 21-day period is probably an error, although it is the gestation period for rats..  Administration by subcutaneous injection.  Caffeine dissolved in sterile saline. | 24 - Sham group-Saline  25 - 10 mg/kg/day caffeine  **Estimated human equivalent**:  none reported. | **Comments:**  - Fetal toxicity in humans appears to be low.  - Moderate to high caffeine consumption may increase risk of spontaneous abortion or low birth weight.  - Study suggests modest maternal caffeine exposure had adverse effects on developing embryonic CV function and growth.  - Transient changes in maternal and fetal blood flow were observed.  - Data suggests further investigation is needed.  - How maternal caffeine intake affects mouse may not reflect human effects. |
| Momozawa K, Fukuda Y. (2003).  Caffeine in fertilization medium is not essential  for bovine IVF by fully capacitated spermatozoa.  *J Reprod Dev.*  49(6):507-12. | - To study if caffeine in fertilization medium for bovine IVF is essential.  - Determine if minimum concentration of caffeine has adverse effect on motility of preincubated spermatozoa. | Bovine in vitro fertilization | In vitro study | 0 mM  5 mM caffeine in fertilization medium  Estimated human equivalent  N/A  **Estimated human equivalent:**  not applicable. | **Comments**:  - Medium without caffeine showed sperm motility significantly higher than in medium with more than 2 mM caffeine.  - Caffeine not essential for bovine in vitro fertilization.  - Caffeine content greater than 2 mM has adverse effect on preincubated (capacitated) sperm motility.  - Medium without caffeine showed sperm motility significantly higher than in medium with more than 2 mM caffeine.  - Caffeine not essential for bovine in vitro fertilization.  - Caffeine content greater than 2 mM has adverse effect on preincubated (capacitated) sperm motility.  **Human Relevance:**  - Study concerns bovine spermatozoa; not applicable to humans. However, should caffeine appear in human sperm, it is probable that it would increase motility. |
| Nomura K, Saito S, Ide K, Kamino Y, Sasahara H, Nakamoto T, Abiko Y. (2004).  Caffeine suppresses the expression of the Bcl-2 mRNA in BeWo cell culture and rat placenta.  *J Nutr Biochem.* 15(6):342-9. | To determine whether caffeine alters gene expressions in human cytotrophoblast-like cell line, BeWo using cDNA microarray technology. | Rats  Pregnant rats  Source and strain not identified.  Study conducted in Japan  Cell: culture  BeWo cell line (CCL-98, American Type Culture Collection, Rockville, MD) cultured with and without caffeine  **Study guidelines:**  Guidelines for experimental animals of Nihon University School of Dentistry at Matsudo, Japan. | In vitro study  Diet  Treated Day 1 (confirmed fertilization) – Day 20. | Group 1 - Control – 20% protein diet  Group 2 – 20% protein diet + 2 mg/100 g bw.  Number of animals in group not specified.  **Estimated human equivalent:** Dosage equivalent to approximately 2 cups of coffee consumed daily. | **Comments:**  - Found expression of (Bcl-2) gene in BeWo cells was down-regulated by caffeine.  - Suggests chronic exposure during gestation could affect embryogenesis.  - Study found significantly decreased level of Bcl-2 mRNA expression, indicates influence of caffeine on placental function.  **Human relevance:**  - Possibility that IUGR and LBW are associated with increased risk of hypertension and coronary heart disease development in men and women adults.  - Suggestion that diseases may be initiated by inadequate supply of nutrition or oxygen in utero.  - Authors express concern for effects of in utero caffeine exposure in later life. |
| Pollard I, Locquet O, Solvar A., Magre S.  (2001).  Effects of caffeine and its reactive metabolites theophylline and theobromine on the differentiating testis.  *Reprod Fertil Dev* 13 (5-6): 435-41. | To compare the outcome of experimental methods of in vivo and in vitro studies and consider the need for confirmation by other test methods**.** | Wistar CF rats  CNRS Colony of R. Janvier, 53680 Le Genest, France  **Study guidelines**: none reported. |  | Organotypic cultures-13-day-old fetal testes primordia  13 day old fetal testes were cultured for 4 days in vitro in the presence of graded doses of caffeine, theophylline or theobromine.  **Estimated human equivalent:** none reported. | **Comments:**  - Caffeine had no effect in vitro indicating that earlier in vivo effects at 30 mg/kg/day on in utero testis development were due to the metabolite theophylline.  **Human relevance:**  - None discussed specifically.  - Authors urge continued testing in vivo as well as in vitro. |
| Saadani-Makki F, Frugière A, Gros F, Gaytan S, Bodineau L. (2004).  Involvement of adenosinergic A1 systems in the occurrence of respiratory perturbations encountered in newborns following an in utero caffeine exposure. A study on brainstem-spinal cord preparations isolated from newborn rats.  *Neuroscience.* 127(2):505-18. | To investigate possible involvement of adenosinergic A_1_ systems in the respiratory perturbations in newborns.  - Study performed on brainstem-spinal cord preparations isolated from newborn rats. | Rats  Pregnant Sprague-Dawley rats  Source not specified.  Study conducted in France.  **Study guidelines**: EEC Directive 24 Nov 1986 (86/609/EEC) regarding animal care and use. | 0.02% caffeine provided in drinking water; changed every 2 days.  Caffeinated water replaced with plain tap water upon parturition.  Beginning of treatment period not specified | 19 Control – drug-free tap water  16 caffeine-administered - 0.02% caffeine in drinking water to pregnant rats – gestation days not identified, but caffeine was removed immediately upon parturition.  Caffeine consumption was estimated at 49.8 mg/kg/day.  **Estimated human equivalent:**  - 70 mg/kg/day ingested by rats equivalent to 30 mg/kg/day for humans.  - Dose considered appropriate level for human model of light caffeine intoxication  - 49.8 mg/kg/day ingested by rats corresponds to moderate human consumption | **Comments:**  - No change in body weight gain in treated dams and non-treated dams. Pup tissue from same dams as produced and evaluated by Bodineau et al. (2003).  **-** In utero exposure caused increase in birth weight of offspring but did not affect litter size significantly.  - Evidence for involvement of adenosinergic A_1_ systems in the occurrence of the respiratory perturbations in newborns following in utero exposure.  - Study shows the importance of the rostral pons in adenosineric A_1_ modulation of the respiratory control.  - Changes in adenosingeric A_1_ system at doses of 49.8 mg/kg in the newborn rat.  **Human relevance**:   - Discussion of animal data only; no specific comparisons to humans made.   Reference use of caffeine for regulation of premature infant respiration. |
| Sahir N, Bahi N, Evrard P, Gressens P. (2000).  Caffeine induces in vivo premature appearance of telencephalic vesicles.  *Dev Brain Res.* 121(2):213-7. | To confirm the effect of caffeine on early encephalization and to determine the consequences of caffeine exposure on subsequent development of the brain and the potential reversibility of any effects.  Provides a model for identifying genes and transduction pathways involved in telencephalic vesicle formation. | Mice  Timed pregnant Swiss mice  Source not identified.  Study conducted in France  **Study guidelines**: none reported. | Gestational days 8-10  IP injection    200 uL phosphate buffered saline (PBS) alone or PBS containing caffeine  Number of encephalized embryos evaluated on Days 9, 10, 13 and 17. | Dams sacrificed on GD9  3 Control – PBS alone  4 – 12.5 mg/kg caffeine + PBS  6 – 25 mg/kg caffeine + PBS  3 – 50 mg/kg caffeine + PBS  Dams sacrificed on GD10  4 Control – PBS alone  3 12.5 mg/kg caffeine + PBS  5 25 mg/kg caffeine + PBS  6 50 mg/kg caffeine + PBS  Dams sacrificed on GD13  2 Control – PBS alone  2 – 50 mg/kg caffeine + PBS  Dams sacrificed on GD17  1 Control – PBS alone  3 – 50 mg/kg caffeine + PBS  **Estimated human equivalent:** compares with high doses of caffeine – 10 to 35 mg/kg/day, consumed orally). | **Comments:**  - Study confirms 2 previous studies that caffeine accelerates the encephalization process.  - Early treatment of embryos may produce the reverse of holoprosencephaly (a recognized of Smith-Lemli-Opitz syndrome, a human disorder due to deficient cholesterol synthesis).  - Caffeine administered to pregnant mice (GDs 8-10) dramatically accelerated primitive neuroepithelium evagination into telencephalic vesicles. |
| Sahir N, Mas C, Bourgeois F, Simonneau M, Evrard P, Gressens P. (2001).  Caffeine-induced telencephalic vesicle evagination in early post-implantation mouse embryos involves cAMP-dependent protein kinase (PKA) inhibition.  *Cereb Cortex.*  11(4):343-9. | To examine the effect of caffeine on gene modulation in post-implantation mouse embryos. | Mice  Pregnant Swiss mice  Source not identified.  Study conducted in France  **Study guidelines:** none reported. | Gestational days 8.5-10.5  IP injection once daily  200 uL phosphate buffered saline (PBS) alone or PBS containing caffeine | 25 mg/kg caffeine + PBS  PBS + PKI(14-22)  5 ug  50 ug  75 ug  PBS + H89  5 ug  50 ug  75 ug  PKI(14-22) and H89 are 2 inhibitors of cAMP-dependent PKA  Number of animals not specified  **Estimated human equivalent:**  none reported. | **Comments:**  - Data suggest that caffeine modulates gene expression of the RIα subunit of PKA and that caffeine-induced inhibition of PKA activity plays a role in early elencephalic evagination.  - Study shows involvement of PKA activity in caffeine-induced acceleration of encephalization.  - Suggests further studies are needed to understand precise mechanisms by which caffeine modulates expression of the RIα subunit of PKA, as well as other potential role of other, unidentified genes differentially expressed in caffeine-exposed embryos. |
| Tatham BG, Feehan, T, Pashen, R. (2003).  Buffalo and cattle hybrid embryo development is decreased by caffeine treatment during in vitro fertilization.  Theriogenology 59 (3-4): 709-717. | - Investigate potential for treating buffalo spermatozoa to overcome problems with buffalo male infertility.  - Determine if buffalo and cattle hybrid embryo development is decreased if treated with caffeine during in vitro fertilization. | Buffalo and cattle hybrid embryo  Buffalo ovaries from local abattoir  Location not identified  Study conducted Australia  **Study guidelines**: none reported. | **In vitro study**  Treated during in vitro process with heparin and caffeine. Heparin and caffeine used in combination for IVF in cattle decreased the time required for oolemma penetration, and in buffalo, these combined agents tended to increase fertilization and embryo development.  Heparin – 2.5 or 5.0 μg/mL heparin and 1 or 2 x 10^6^ spermatozoa/mL. For caffeine 2.5, 5.0, 7.5 or 10 μg/mL | Treated with caffeine  **Estimated human equivalent:**  not applicable | **Comments:**  - Studies indicate that problems with reproduction in the buffalo are specific to individual bulls. The common treatments of heparin and caffeine to induce capcitation for IVF had different results on fertilization and embryo development. Caffeine treatment of spermatozoa during fertilization increased binding of the zona pellucida and penetration of the oocyte, which tended to decrease embryo development.  - Further problems associated with infertility likely to be related to female buffalo hormonal and nutritional status and oocyte viability. |
| Tomimatsu T, Lee SJ, Peña JP, Ross JM, Lang JA, Longo LD. (2007).  Maternal caffeine administration and cerebral oxygenation in near-term fetal sheep.  *Reprod Sci* 14(6):588-94 | Test hypothesis that maternal caffeine administration does not significantly alter fetal cerebral oxygenation. Ultimately wanted to evaluated whether the effects of caffeine on fetal behavior, sleep-awake cycle and other adenosine-mediated physiological functions might last longer than predicted on the basis of non-pregnant adult data. | Sheep  Pregnant Western ewes from Nebeker Ranch, Lancaster, CA  **Study guidelines**:  Procedures in accordance with regulations of National Institutes of Health Guide for Care and Use of Laboratory Animals. | Ewes administered caffeine by iv.  7 pregnant ewes and their fetuses were instrumented at 125 ± 3 days gestation (term ~ 145 days)  Surgical procedure for placement of catheters in fetal heads described. | Infused 400 mg of caffeine about 8 mg/kg over 30 minutes – 2 to 3 cups of coffee containing 100 to 200 mg caffeine.  In near term fetal sheep  **Estimated human equivalent:** 400 mg of caffeine about 8 mg/kg over 30 minutes – 2 to 3 cups of coffee containing 100 to 200 mg caffeine. (Different route) | **Comments:**  - There was some compromise of fetal cerebral oxygenation.  - No overall affect on fetal oxygen supply.  - What this means is not clear.  - Authors suggest pregnant women should limit caffeine intake, especially in instances where fetal oxygenation may be compromise.  - More studies needed to determine long-term neurological and behavioral effects on the developing fetus of caffeine intake during pregnancy. |
